# Polar Order in a Fluid Like Ferroelectric with a Tilted Lamellar Structure – Observation of a Polar Smectic C (SmC_P_) Phase

**DOI:** 10.1002/anie.202416545

**Published:** 2024-11-14

**Authors:** Jordan Hobbs, Calum J. Gibb, Damian Pociecha, Jadwiga Szydłowska, Ewa Górecka, Richard J. Mandle

**Affiliations:** ^1^ School of Physics and Astronomy University of Leeds Leeds UK LS2 9JT; ^2^ School of Chemistry University of Leeds Leeds UK LS2 9JT; ^3^ Faculty of Chemistry University of Warsaw ul. Zwirki i Wigury 101 02-089 Warsaw Poland

**Keywords:** Materials science, Liquid crystals, ferroelectric materials, smectic phases, X-ray scattering

## Abstract

The discovery of fluid states of matter with spontaneous bulk polar order is appreciated as a major discovery in the fields of soft matter and liquid crystals. Typically, this manifests as polar order superimposed atop conventional phase structures and is thus far limited to orthogonal phase types. Here we report a family of materials which exhibit a previously unseen state of matter which we conclude is a polar smectic C phase, and so we term it SmC_P_. The spontaneous polarisation of the SmC_P_ phase is over two orders of magnitude larger than that found in conventional ferroelectric SmC phase of chiral materials used in some LCD devices. Fully atomistic molecular dynamics simulations faithfully and spontaneously reproduce the proposed structure and associated bulk properties; comparison of experimental and simulated X‐ray scattering patterns shows excellent agreement. The materials disclosed here have significantly smaller dipole moments than typical polar liquid crystals such as RM734 which suggests the role of molecular electrical polarity in generating polar order is perhaps overstated, a view supported by consideration of other molecular systems.

## Graphical abstract

Chiral smectic C liquid crystals (SmC*) require a chiral centre in the molecular structure and result in a spontaneous polarisation significantly lower than solid state ferroelectrics. Here we present the discovery of a non‐chiral SmC liquid crystal which forms a longitudinally ferroelectric phase with polarisation two orders of magnitude larger than “classic” ferroelectric SmC* materials.

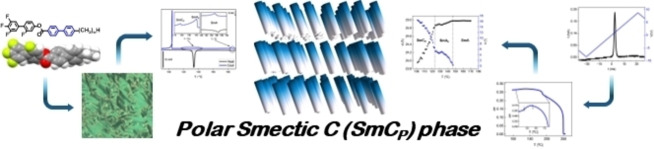

## Introduction

**Figure 1:**
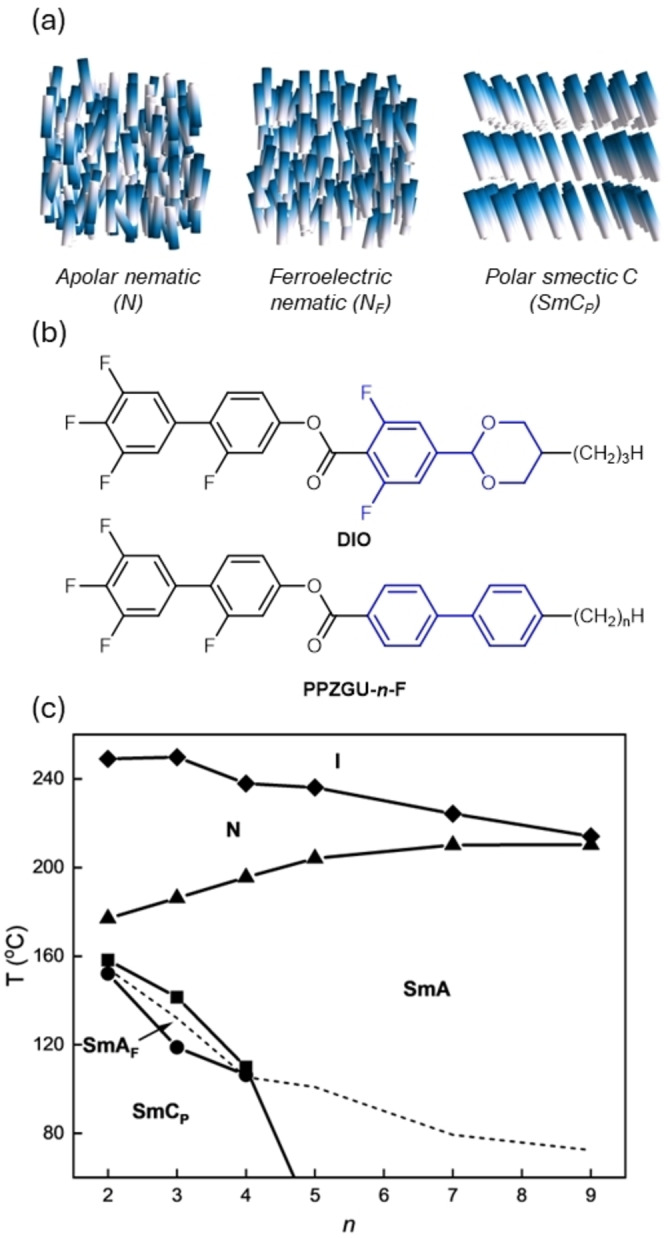
Schematic representations of the **(a)** conventional N, N_F_ and SmC_P_ mesophases; **(b)** the chemical structure of the archetypal N_F_ materials **DIO** ^[3]^ and the general structure of the **PPZGU‐*n*‐F** materials reported in this work. The difference in molecular structure between **DIO** and **PPZGU‐n‐F** are highlighted in blue; **(c)** phase diagram showing the evolution of phase behavior on increasing alkyl chain length (***n***) for the **PPZGU‐*n*‐F** materials. Melting points are denoted by the dotted line.

Since its discovery in 2017 [^1^, ^2^, ^3^] and subsequent confirmation of discovery in 2020,[^4^, ^5^, ^6^, ^7^] the ferroelectric nematic phase (N_F_) has been shown to have a rich variety of properties such as non‐linear optical properties,^[8]^ extreme interfacial instabilities,^[9]^ bulk photovoltaic effect,^[10]^ extreme polarisation screening,^[11]^ and piezoelectric properties ^[12]^ to name a few, where all these properties have their origins in the large longitudinal spontaneous polarisation (> 4 μC cm^‐2^) caused by the lack of inversion symmetry of the N_F_ phase (Figure 1a) .[^13^, ^14^]

Longitudinal polarisation of liquid crystalline materials is not isolated to the nematic phase and more exotic phase structures have been discovered ^[14]^ such as the ferroelectric smectic A (SmA_F_) phase [^15^, ^16^, ^17^] where the polarisation vector is parallel to the layer normal. An antiferroelectric smectic A (SmA_AF_) phase has also recently been discovered [^18^, ^19^] although the exact nature of the anti‐ferroelectric ordering within those systems is not yet clear. Perhaps even more interesting is the recent discovery of longitudinally polar LC phases which spontaneously break chiral symmetry, presumably due to the high spontaneous polarisation, such as the so‐called twist bend ferroelectric nematic phase (N_TBF_) [^20^, ^21^] and the heliconical polar smectic C phase (SmC^H^_P_) .^[18]^ It has recently been suggested that since these LC phases with longitudinal polarity are proper ferroelectrics (i.e. caused by dipolar interactions rather than steric interactions) polar order could effectively be superimposed over the orientation and positional order of the underlying LC phase .^[19]^ A logical progression of this idea would suggest the existence of longitudinally polar variants of many of the known non‐polar LC phases.

In apolar liquid crystals, it is widely understood that fluorination of the rigid core unit generally suppresses smectic phase formation [^22^, ^23^] and it was shown that removal of adjunct fluorine atoms from the archetypal ferroelectric nematogen **DIO** ^[3]^ leads to the formation of a SmA_F_ phase .^[16]^ The dioxane moiety found in **DIO** is also known to supress smectic phase formation .^[24]^ Most studies into novel ferroelectric nematogens and smectogens have focused on increasing molecular fluorination ,^[25]^ here we show that exchange of the dioxane ring for a phenyl moiety coupled with the removal of all adjunct fluorine atoms (the **PPZGU‐*n*‐F** series, Figure 1b **)** further promotes the formation of smectic phases with bulk polar order, leading to the observation of a both a SmA_F_ phase and a polar, titled smectic phase (Figure 1a [right]).

## Results and Discussion

Transition temperatures of the **PPZGU‐*n*‐F** materials are given in Figure 1c (tabulated data in the ESI; Table S1) and were determined by differential scanning calorimetry (DSC), with phase assignments made by polarised optical microscopy (POM), assisted by X‐ray scattering and current response studies (*vide infra*). For the sake of brevity, we will focus our discussion on the homologue with *n*= 3 as a representative example of the phase behaviour of the **PPZGU‐*n*‐F** series with supplementary results for the other materials found in the ESI.

**Figure 2:**
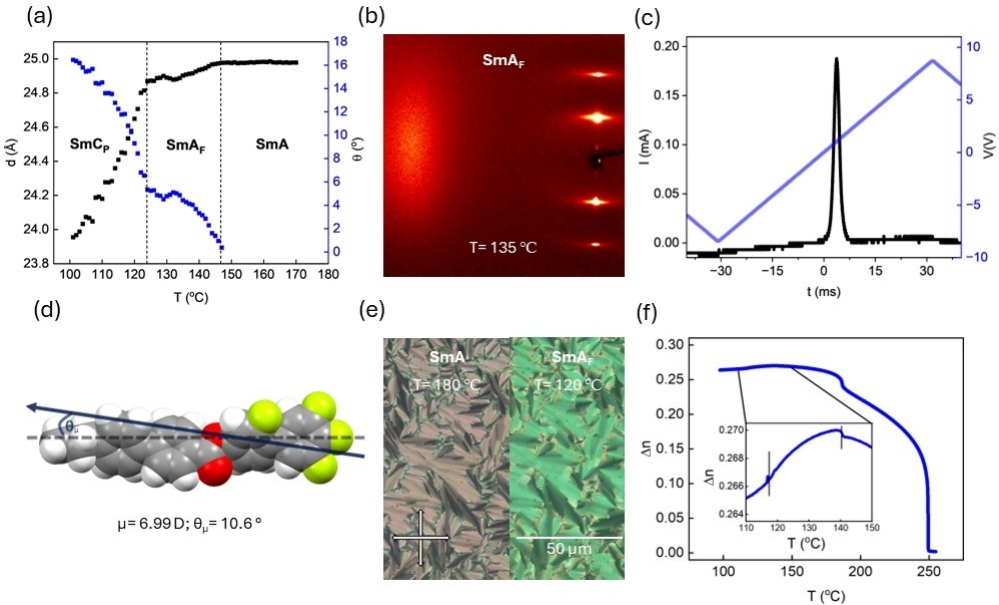
(a) Temperature dependence of the layer spacing for **PPZGU‐3‐F** in the SmA, SmA_F_ and SmC_P_ phases, Tilt angle for the SmA_F_ phase is the angle between layer normal and long molecular axis assuming a conical De Vries like molecular distribution as described in the text while for the SmC_P_ phase it is a general bulk uniform tilt angle. (b) 2D x‐ray diffraction pattern for **PPZGU‐3‐F** in the SmA_F_ phase (T= 135 °C), (c) current response trace measured at 8 Hz in the SmA_F_ phase (T = 136 °C), (d) the energy minimised geometry of **PPZGU‐3‐F** calculated at the B3LYP‐GD3BJ/cc‐pVTZ level of DFT. The long molecular axis is marked by the hashed line with the direction of the longitudinal molecular dipole moment (inclined at an angle relative to the long molecular axis) marked by the arrow (μ= 6.99 D; θμ= 10.6 °), (e) POM micrographs of the SmA (T= 180 °C) [left] and SmA_F_ [right] (T= 120 °C) phases. **PPZGU‐3‐F** samples are confined in thin cells treated for a homeotropic anchoring condition, and (f) the temperature dependence of the optical birefringence, T(*Δn*), for **PPZGU‐3‐F** measured using green light, λ= 532 nm in a 1.7 μm cell treated for planar alignment.

**PPZGU‐3‐F** shows apolar nematic (N) and smectic A (SmA) phases (as do all other homologues), identified using POM and X‐ray scattering, as well as two further LC phases observed on further cooling. Confinement between untreated glass slides typically resulted in homeotropic alignment for the N and SmA phases while for the two lower temperature phases, uncharacteristic textures were observed. However, confinement in cells treated for homeotropic alignment resulted instead in degenerate planar alignment (Figure S1). In X‐ray scattering experiments, both the N and SmA phases show diffuse scattering at wide‐angles, indicating liquid‐like order in direction perpendicular to the director. The lamellar structure of the SmA phase leads to the Bragg scattering of X‐rays at small angles (whereas the nematic has only diffuse scattering), yielding a layer spacing (*d*) of 25.0 Å (Figure 2a) which is largely temperature independent across the entire phase range. To obtain the layer spacing (*d*) as a ratio of molecular length (*L*) we performed molecular geometry optimisation at the B3LYP‐GD3BJ/cc‐pVTZ level; using this value affords a ratio of ~ 1.07, indicating a near monolayer arrangement of the molecules within the smectic layers. Current response measurements demonstrate that the N and SmA phases are apolar, with only small ionic and dielectric contributions being detected. Notably however, below 150 °C (still in the SmA phase), a single broad peak centred around polarity reversal is observed in the SmA phase (Figure S4), presumably the result of pre‐transitional field induced polarisation.

For the three shortest homologues studied (*n*=2‐4), two further phases are observed upon cooling the apolar SmA phase. X‐ray scattering shows that both phases have a liquid‐like smectic structure, with diffuse scattering at wide angles and sharp Bragg scattering in the small angle region (Figure 2b). Subsequent experiments, detailed below, identify these phases as SmA_F_ and a newly discovered tilted phase with bulk polar order. At the transition from the SmA into the SmA_F_ phase (i.e. the onset of polar order signified by a single peak in the current response, Figure 2c) there is a slight contraction of layer spacing while the low‐angle peak remains a single lobe. From quantum chemical calculations at the B3LYP‐GD3BJ/cc‐pVTZ level of DFT **PPZGU‐3‐F** is shown to have an angle of 10.7 ° between the dipole vector and the long molecular axis (Figure 2d, Table S2). A SmA_F_ phase comprised of such molecules could result in a layered structure where the dipoles are aligned normal to the layer planes with the long molecular axes inclined at an angle relative to the layer normal. For this phase to be uniaxial, and be SmA type rather than SmC, the inclined molecules would need to be distributed around a cone and such a structure would be analogous to a de Vries SmA structure.^[25]^ Such a structure would explain the reduction in layer spacing (Figure 2a) in the SmA_F_ phase. While the proposed tilt angle here is less than the 10.7 ° suggested by the DFT calculations this is perhaps not so surprising as the DFT calculations are of a single gas phase molecule and are not completely representative of a molecule in the condensed LC phases. There would also be further contributions to the tilt angle from the polar and orientational order of the phase. We also note for the Bragg scattering associated with the lamellar structure up to 6^th^ order harmonic can be observed, although this limit is due to the Q limits of the detector rather than because we could not observe the 7^th^ order fringe (Figure S3). Generally, for a SmA phase the layers are diffuse and more of a density modulation rather than strict layers. However, the observation here of several harmonics of the diffraction signal due to layered structure means that the density modulation along the layer normal is strongly non‐sinusoidal giving rather sharp boundaries between the high‐ and low‐density regions. The long‐range correlation of the layers may be due to a lack of layer undulations as these would lead to areas of bound charge due to splay of the polarisation in those regions.^[26]^

For the SmA_F_ phase the current response shows that the pretransition peak observed for the SmA phase moves to higher voltage, indicating the increased threshold for switching (Figure 2c). This increase is possibly due to the increased cost of rotating the smectic layers along with the polarisation where the maximum value of polarisation in the SmA_F_ phase is ~3.0 μC cm^‐2^ (Figure S5). The non‐centrosymmetric structure of the SmA_F_ phase is further affirmed by second harmonic generation (SHG) microscopy (Figure S6), where the presence of an SHG signal without biasing electric field confirms the polar ground state of the SmA_F_ phase.

Optically, the SmA_F_ phase retains the fan texture observed in the proceeding apolar SmA phase, however the texture now contains regions of rapidly changing retardance (Figure S1). These defects generally form out of fan domain boundaries of the SmA phase, indicative of the rotation of the vertical director at regions where the sign of the polarization switches without forming a line defect (Figure 2e). Such observations are suggested to be characteristic of the SmA_F_ phase .[^18^, ^19^] We propose that the texture observed in these materials is somewhat paramorphotic, as it has been suggested that polar ordering prohibits the formation of Dupin cyclides responsible for the fan and focal conic textures in the apolar SmA phase.^[16]^ In anti‐parallel rubbed planar cells, a clear transition can only be observed when comparing the dark states of both phases via comparison of the defects which form around the spacer beads (Figure S7). In general, when anti‐parallel rubbed planar cells are used to confine the N_F_ phase, π‐twist domains can form, due to polar surface interactions ^[28]^ and to minimize the bulk polarisation of the system .^[29]^ Such domains are not observed within the SmA_F_ phases studied here, consistent for when the phase is formed from a preceding N_F_ phase .^[15]^ Optical birefringence (Δ*n*) was measured by cooling throughout the whole LC phase range (Figure 2f). In the N phase a power law temperature dependence was observed, with a small jump in Δ*n* on transition to the SmA phase. Another slight step (~0.01) was detected for the SmA‐SmA_F_ transition before a slight decrease is observed throughout the SmA_F_ phase. Although this could be due to a slight reduction in alignment quality, POM images of the dark state of these materials does not imply any change in the quality of alignment and we instead suggest that this is further evidence for the adoption of a de Vries like molecular orientation.

**Figure 3:**
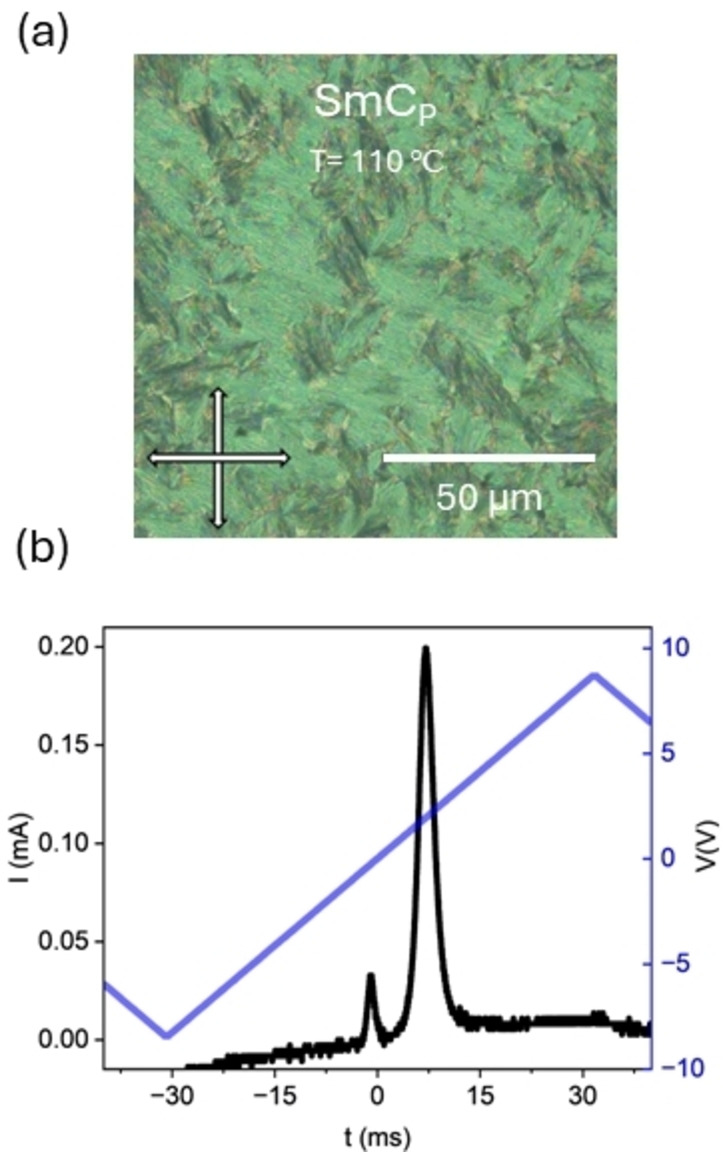
**(a)** POM micrographs of the broken fan texture of the SmC_P_ (T= 110 °C) phase observed for **PPZGU‐3‐F**. The image is taken of a sample confined within a thin cell treated for homeotropic anchoring; **(b)** current response trace measured at 8 Hz in the SmC_P_ phase (T = 104 °C) showing the emergence of a second smaller peak at longer time scales.

Further cooling of the SmA_F_ phase for homologues with *n* < 5 sees a transition to a final liquid‐like smectic phase. Here, the Bragg scattering of the SmA_F_ is retained though a continuous decrease in the layer spacing, indicating a tilting of the molecules away from the layer normal (i.e. a SmC‐like phase), is now observed. For all the homologues studied, the tilt does not saturate at the point of crystallisation (~ 16 ° at 100 °C for **PPZGU‐3‐F**; Figure 2a). Within a cell treated for homeotropic anchoring, the fan‐like texture of the preceding SmA_F_ splits into small domains, forming a broken fan‐like texture (Figure 3a), where each domain corresponds to different tilt direction. The formation of these domains disrupts the alignment of the sample when confined within cells treated for planar anchoring, resulting in the complete loss of any extinction condition upon rotating the cell with respect to the polarisers’ direction (Figure S8d). The lack of alignment in planar cells prohibits the reliable measurement of birefringence via the technique used here. Measurements of the current response reveal that the polarity of the SmA_F_ phase is retained (Figure 3b), with the position of the main peak observed in the preceding phase moving backwards towards lower voltage indicating a decreased elastic cost of bulk polarisation reversal, with the P_S_ saturating at a value of 3.3 μC cm^‐2^ a value two orders of magnitude larger than for the conventional chiral smectic C (SmC*) phase .^[30]^ However, a second peak of a smaller magnitude to the main signal, emerges in the current response upon entering the new phase just before applied voltage polarity reversal. Such a current response would not result in a truly “ferroelectric” hysteresis loop, owing to the observation of multiple peaks. This peak is possibly due to some ionic effect induced by the emergence of tilt domains, or it could be associated with tilt removal, analogous to a mechanism suggested for the recently discovered helical polar SmC phase ,^[18]^ though we note no evidence for helix formation is observed for the materials presented here. The polar nature of the lowest temperature smectic phase is further confirmed by SHG microscopy (Figure S6) though whether the phase is “ferroelectric” or some other type of polar ordering is not yet clear from performed measurements and thus we term this phase the polar smectic C phase (SmC_P_).

We sought to validate the structure proposed above using fully atomistic molecular dynamics simulations, during which (and under certain conditions) the SmC_P_ phase forms spontaneously. Our simulations initially begin from either an isotropic or polar nematic configuration, which evolve into apolar and polar phases, respectively. Gratifyingly, both apolar and polar initial configurations yield the correct phase sequences, with bulk properties and transition temperatures in reasonable agreement with experiment. Simulations commencing from an isotropic state remain isotropic at and above 252 °C, while the simulation at 227 °C evolves into a nematic phase (<*P*2> = 0.61 +/‐ 0.04) (Figure S8). At and below 202 °C, the simulations evolve into an SmA phase with layer spacing of 2.4 +/‐ 0.1 nm (Figure S10). At no point does the apolar simulation evolve into a tilted smectic phase.

**Figure 4:**
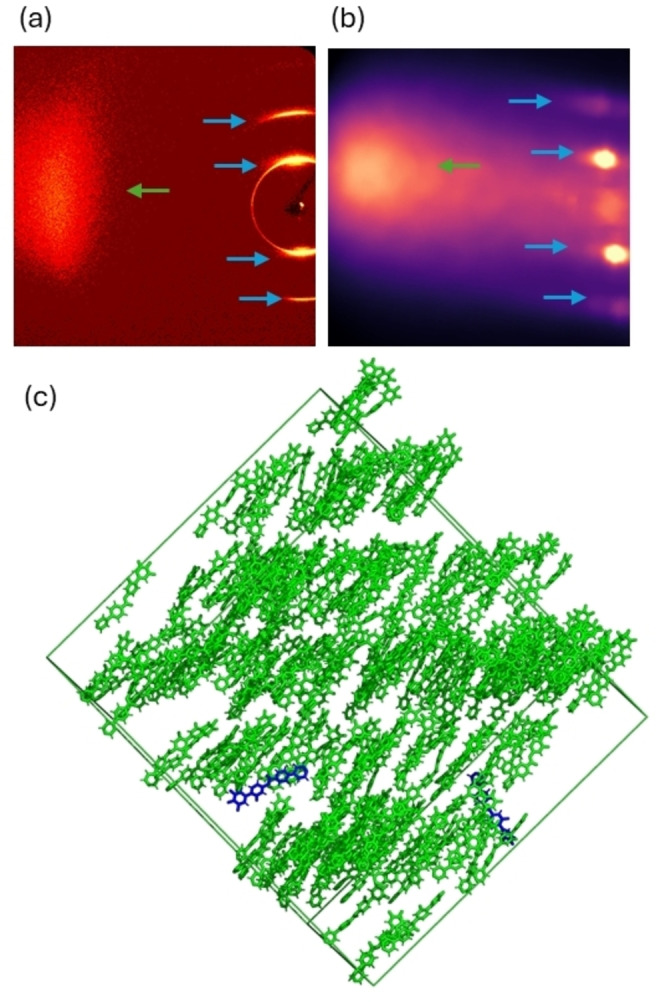
**(a)** an experimental 2D x‐ray diffraction pattern for **PPZGU‐3‐F** in the SmC_P_ phase (T= 115 °C); **(b)** simulated 2D SAXS pattern of the SmC_P_ phase of **PPZGU‐3‐F** at 127 °C based on the MD trajectory showing good agreement with the data obtained by experiment; and **(c)** Instantaneous configuration of the *polar* smectic C phase for the ***n*** **= 3** homologue formed spontaneously from the initial polar configuration at the same temperature (<*P*2> = 0.78 +/‐ 0.06). The snapshot is color‐coded green/blue to show molecules whose dipole is parallel or antiparallel with the director, respectively (<*P*1> = 0.90 +/‐ 0.04). The hydrocarbon chains are omitted from the rendering to aid visualisation of the layer structure (layer spacing = 2.3 +/‐ 0.1 nm; tilt angle = 25 +/‐ 3 °). The calculated spontaneous polarisation (P_s_) is 3.2 +/‐ 0.01 μC.cm‐^2^. Only 400 molecules (out of 1000) are shown, to aid visualisation.

Simulations commencing from a polar nematic configuration evolve into an isotropic state (at and above 252 °C) and slowly transform to a non‐polar N phase on cooling (227 °C). The orientational order parameter is retained, while the polar order parameter tends to zero over the first 140 ns (Figure S9). At and below 202 °C, a polar SmA phase spontaneously forms with <*P*1> = 0.94 +/‐ 0.01 and <*P*2> = 0.78 +/‐ 0.02. The simulated SmA phase has a layer spacing of 2.3 (+/‐ 0.1) nm and spontaneous polarisation value of 2.5 (+/‐ 0.02) μC cm^‐2^, both of which are in harmony with the values obtained by experiment. Crucially, at and below 202 °C, the molecules spontaneously tilt away from the layer normal, resulting in the formation of a tilted smectic phase with bulk polar order, analogous to the SmC_P_ phase proposed based on experimental observations (Figure 4c). The *in‐silico* SmC_P_ phase has a layer spacing of 2.2 +/‐ 0.1 nm and a tilt angle of 25 +/‐ 3 °, which are in reasonable agreement with experimentally obtained values (2.4 nm and 16 °, respectively). The SmC_P_ phase has a near saturated polar order parameter (<*P*1> = 0.90 +/‐ 0.04) and orientational order consistent with a fluid smectic phase (<*P*2> = 0.78 +/‐ 0.06).

We used the MD trajectory to simulate a 2D SAXS pattern of the SmC_P_ phase by mapping the atomic electron density onto a 3D grid and transforming into the structure factor via Fourier transform (see ESI). The simulated 2D SAXS pattern closely matches that obtained experimentally (Figure 4a and 4b), with diffuse wide‐angle scattering and multiple high intensity low‐angle peaks. Periodic noise in the simulated SAXS background arises from the Fourier Transform procedure. Additionally, the system size (1000 molecules) and limited simulation duration (500 ns) conspire to introduce further artifacts, such as finite‐size effects and incomplete sampling of long‐range correlations. The qualitative agreement between experimental/simulated 2D SAXS patterns, along with the ability of the MD simulation to accurately recreate experimentally measured properties, gives a high degree of confidence in our model of the SmC_P_ phase.

**Figure 5:**
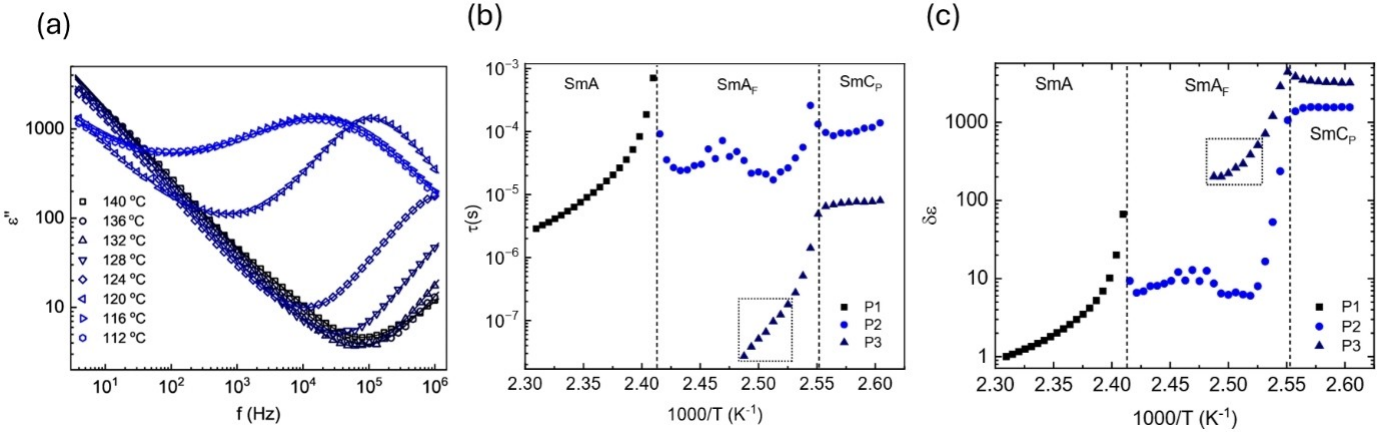
(a) Representative dielectric loss spectra for **PPZGU‐3‐F** in the SmA_F_ and SmC_P_ phases. Line through the data corresponds to the fits obtained; (b) Arrhenius plot of relaxation times; and (c) dielectric strength parameter obtained. The dashed box area in each plot corresponds to areas where P3 was at the edge and outside the fitting window reducing the fit quality in that region.

Although the interpretation of the dielectrics of longitudinally polar liquid crystal phases is not yet fully understood,[^31^, ^32^, ^33^, ^34^, ^35^] we elected to probe the dynamic properties of **PPZGU‐3‐F** by dielectric spectroscopy in the SmA, SmA_F_ and SmC_P_ phases. Complex permittivity data was obtained for samples in 10 μm thick cells with untreated gold electrodes (**Figure S12a, S12b**), with the caveat that the dielectric strengths and relaxation times of any relaxation processes will be dependent on the sample thickness, orientation, and anchoring behaviour. Figure 5a contains representative dielectric loss data for **PPZGU‐3‐F** for the SmA_F_ and SmC_P_ phases (details of fitting given in ESI with example of the deconvoluted fits, and Figures 5b and 5c show the relaxation times and dielectric strength for the processes observed through the SmA, SmA_F_ and SmC_P_ phases (α fitting parameter is shown in Figure S12c). In the SmA phase, only a single dielectric process was observed (P1). Power laws were used to account for a low frequency conductivity flank and for any processes observed at timescales faster than the frequency window. P1 shows a super‐Arrhenius, Vogel‐Fulcher‐Tammann (VFT) dependence of its relaxation times typical of cooperative processes with asymptotic behaviour at the transition to the SmA_F_ phase. We suggest that this process is related to rotations around the short molecular axis which become frozen out at the SmA‐SmA_F_ transitions due to the emergence of polar order. Due to the formation of small ferroelectric clusters in the SmA phase, indicated by the strong pre‐transitional induced polarisation (Figure S5), the dielectric strength of P1 also shows significant growth approaching the transition to the polar phase (~100 at the SmA‐SmA_F_ transition), behaviour analogous to the N_F_ phase .^[36]^

In the SmA_F_ phase two processes are observed. A weak mode (P2) appears with a timescale of roughly 10^‐5^ s and very large distribution parameter. The timescales and dielectric strength of this process are essentially temperature independent in the SmA_F_ phase, only consistently moving to longer timescales and steeply increasing in strength (circa 2 orders of magnitude) upon approach to the SmC_P_ phase. The main relaxation mode observed in the SmA_F_ phase (P3) is moving into the frequency window from shorter timescales. As the SmC_P_ phase is approached, P3 steeply increases in strength and slows down (Figure 5b, c). In the SmC_P_ phase, the strong P3 dominates dielectric response, it is only weakly temperature dependent for both its timescale and dielectric strength (in fitting procedure the much weaker P2 mode was also used to ensure the continuous evolution of the modes). We suggest that P3 that starts to develop in SmA_F_ phase is related to tilt fluctuations, which increase in strength as the molecular motions become more cooperative near phase transition to tilted SmC_P_ phase. In SmC_P_ phase the relaxation process P3 is attributed to collective rotation of molecules, and thus polarization, on the tilt cone.

**Figure 6:**
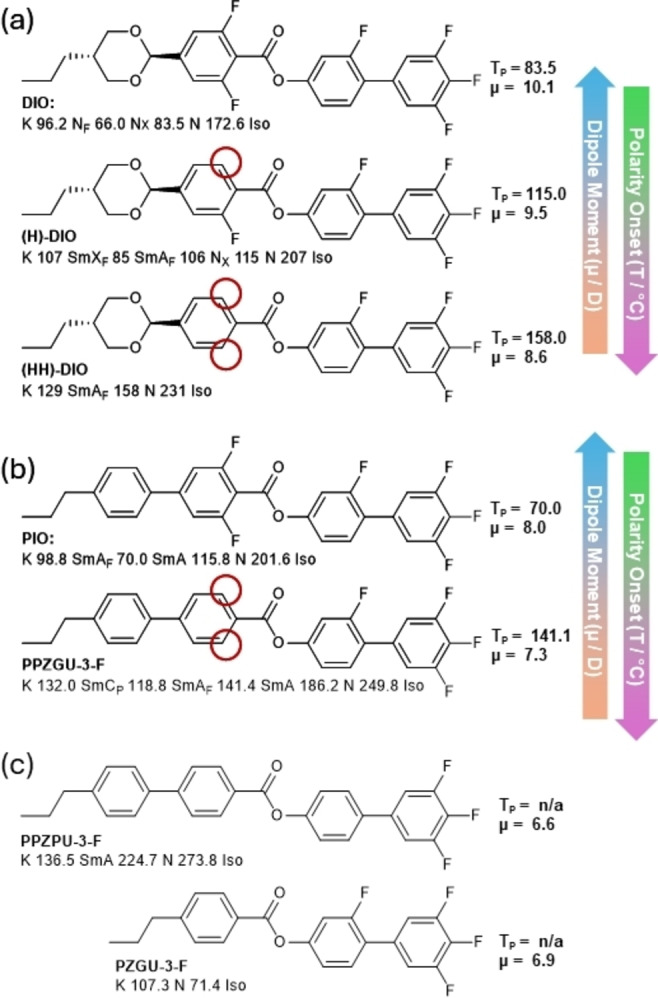
Molecular structures, transition temperatures (°C) and dipole moments (μ; at the B3LYP‐GD3BJ/cc‐pVTZ level of DFT) of **(a) DIO** ^[3]^ and similar selected analogues;^[16]^ **(b) PIO** ^[19]^ and **PPZGU‐3‐F**, the later from this work; **(c)** the non‐polar derivatives of **PPZGU‐3‐F** and **PIO**, **PPZPU‐3‐F** (of this work) and **PZGU‐3‐F**,^[19]^ respectively.

Ultimately the generation of polar ordering in real‐world experimental conditions is principally a product of molecular structure, and while a full structure‐property relationship is beyond the scope of this initial communication, some striking trends are immediately apparent. Firstly, considering **DIO** ^[3]^ as a starting point, we see that the removing of fluorine atoms from the molecular structure gives a progressively higher onset temperature for polar ordering (either to N_F_, N_X_, or SmA_F_; Figure 6a).^[16]^ Similarly, replacement of the dioxane unit of **DIO** with phenyl ring ‐ material **PIO** ^[19]^ ‐ increases the propensity for smectic phase formation (Figure 6b). Further reduction of the molecular polarity, achieved by removing fluorine substituents from **PIO**, yields the **PPZGU‐*n*‐F** materials reported here (Figure 6b) which dramatically increases the onset temperature of polar ordering. We also note that replacement of the dioxane moiety with a phenyl ring only decreases polar‐apolar transition temperature (TP) by 10 °C despite the significant reduction (1‐2 D) in dipole moment. These two observations run counter to the view that molecular polarity alone generates bulk polar ordering, evidenced by calculated molecular dipole moments which are given in Figure 6b, and we note that the molecules presented here result in one of the lowest reported molecule dipoles to form an LC phase .^[37]^ Even further reduction in polarity by removing subsequent fluorine atoms gives non‐polar phase types (**PPZPU‐3‐F**; this work, see ESI). We also note the importance of molecular length in arresting head‐to‐tail flipping, as evidenced by **PZGU‐3‐F** .^[19]^ **(**Figure 6c), which is non‐polar. Generally, as the degree of fluorination decreases the propensity for smectic phase formation increases, which has led us to the discovery of the SmC_P_ phase.

An important observation regarding the materials presented here is that polar order is only seen in shorter homologues (***n*** < 5) which is likely a consequence of the increased physical separation mandated by long terminal chains disrupting the head‐to‐tail packing of molecules. Empirically, this reinforces the rather general observation of the importance of head‐to‐tail correlations between molecules in the formation of polar LC phase .[^1^, ^4^, ^21^, ^38^, ^39^, ^40^]

## Conclusion

We have presented evidence for a new class of polar ordered materials with a titled lamellar structure which we conclude to be an analogue of the traditional SmC phase, which we term the SmC_P_ phase. The SmC_P_ phase presents as a conventional tilted smectic phase with superimposed polar order with the direction of polarisation being along the director and tilted away from the layer normal. This polar SmC phase occurs without helix formation as was reported recently ,^[18]^ and similar phase sequence has been seen recently reported by others .[^41^, ^42^] In the same materials we also observe a polar orthogonal smectic phase (SmA_F_) but with an unusual heliconical distribution of molecules. The observation of polar ordered phases in the structures presented here is surprising given their rather modest polarity, with dipole moments comparable to 5CB. Both the SmA_F_ and the SmC_P_ phase emerge spontaneously in MD simulations, further highlighting the power of this simulation technique as a computational microscope for the investigation of polar liquid crystalline systems.

## Supporting Information

The data that support the findings of this article are available in the Supporting Information of this article with the raw data associated with this paper openly available from the University of Leeds Data Repository at https://doi.org/10.5518/1573.

## Author contributions

J.H. and C.J.G contributed equally to this work; C.J.G. and R.J.M; performed chemical synthesis; J.H. and C.J.G. performed microscopy and birefringence studies; J.H. performed applied field studies and DSC analysis; D.P performed X‐ray scattering experiments, assisted by J. H and C.J.G; D.P. performed dielectric spectroscopy which was analysed by J.H; J.S. performed SHG microscopy measurements; J.H. and R.J.M. performed electronic structure calculations; R.J.M performed molecular dynamics simulations; R.J.M. and D.P secured funding. The initial manuscript was written jointly by J.H, C.J.G, and R.J.M before being subsequently reviewed with contributions from all authors.

## Conflict of Interests

The authors declare no conflict of interest.

## Supporting information

As a service to our authors and readers, this journal provides supporting information supplied by the authors. Such materials are peer reviewed and may be re‐organized for online delivery, but are not copy‐edited or typeset. Technical support issues arising from supporting information (other than missing files) should be addressed to the authors.

Supporting Information

## References

[1] R. J. Mandle , S. J. Cowling , J. W. Goodby , Chem. Eur. J. 2017, 23, 14554. 28850751 10.1002/chem.201702742 PMC5656819

[2] R. J. Mandle , S. J. Cowling , J. W. Goodby , Phys. Chem. Chem. Phys. 2017, 19, 11429. 28422219 10.1039/c7cp00456g

[3] H. Nishikawa , K. Shiroshita , H. Higuchi , Y. Okumura , Y. Haseba , S. I. Yamamoto , K. Sago , H. Kikuchi , Adv. Mater. 2017, 29, 1702354. 10.1002/adma.201702354 29023971

[4] O. D. Lavrentovich , Proc. Natl. Acad. Sci. U.S.A. 2020, 117, 14629. 32541021 10.1073/pnas.2008947117 PMC7334533

[5] N. Sebastián , L. Cmok , R. J. Mandle , M. R. De La Fuente , I. Drevenšek Olenik , M. Čopič , A. Mertelj , Phys. Rev. Lett. 2020, 124, 037801. 32031856 10.1103/PhysRevLett.124.037801

[6] R. J. Mandle , N. Sebastián , J. Martinez-Perdiguero , A. Mertelj , Nat. Commun. 2021, 12, 4962. 34400645 10.1038/s41467-021-25231-0 PMC8367997

[7] X. Chen et al., Proc. Natl. Acad. Sci. U.S.A. 2020, 117, 14021.32522878

[8] V. Sultanov , A. Kavčič , E. Kokkinakis , N. Sebastián , M. V. Chekhova , M. Humar , Nature 2024, 631, 294. 38867054 10.1038/s41586-024-07543-5 PMC11236711

[9] R. Barboza , S. Marni , F. Ciciulla , A. Mir , G. Nava , F. Caimi , A. Zaltron , N. A. Clark , T. Bellini , L. Lucchetti , Proc. Natl. Acad. Sci. U.S.A. 2022, 119, e2207858119. 35914148 10.1073/pnas.2207858119 PMC9371712

[10] F. Hassan et al., Opt. Lett. 2024, 49, 4662. 39146129 10.1364/OL.527568

[11] F. Caimi , G. Nava , S. Fuschetto , L. Lucchetti , P. Paiè , R. Osellame , X. Chen , N. A. Clark , M. A. Glaser , T. Bellini , Nat. Phys. 2023, 19, 1658.

[12] M. T. Máthé , M. S. H. Himel , A. Adaka , J. T. Gleeson , S. Sprunt , P. Salamon , A. Jákli , Adv. Funct. Mater. 2024, 34, 2314158.

[13] N. Sebastián , M. Čopič , A. Mertelj , Phys. Rev. E 2022, 106, 021001. 36109969 10.1103/PhysRevE.106.021001

[14] Y. Song , S. Aya , M. Huang , Giant 2024, 19, 100318.

[15] X. Chen et al., Proc. Natl. Acad. Sci. U.S.A. 2022, 119, e2210062119.36375062

[16] H. Kikuchi , H. Matsukizono , K. Iwamatsu , S. Endo , S. Anan , Y. Okumura , Adv. Sci. 2022, 9, 2202048. 10.1002/advs.202202048 PMC9475520 35869031

[17] Y. Song , M. Deng , Z. Wang , J. Li , H. Lei , Z. Wan , R. Xia , S. Aya , M. Huang , J. Phys. Chem. Lett. 2022, 13, 9983. 36263973 10.1021/acs.jpclett.2c02846

[18] C. J. Gibb , J. Hobbs , D. I. Nikolova , T. Raistrick , S. R. Berrow , A. Mertelj , N. Osterman , N. Sebastián , H. F. Gleeson , R. J. Mandle , Nat. Commun. 2024, 15, 5845. 38992039 10.1038/s41467-024-50230-2 PMC11239904

[19] J. Hobbs , C. J. Gibb , R. J. Mandle , Small Sci. 2024, 4, 2400189. 10.1002/smsc.202400189 PMC11935032 40212248

[20] J. Karcz , J. Herman , N. Rychłowicz , P. Kula , E. Górecka , J. Szydlowska , P. W. Majewski , D. Pociecha , Science 2024, 384, 1096. 38843325 10.1126/science.adn6812

[21] H. Nishikawa , D. Okada , D. Kwaria , A. Nihonyanagi , M. Kuwayama , M. Hoshino , F. Araoka , Adv. Sci. 2024, 2405718. 10.1002/advs.202405718. PMC11633337 39099380

[22] M. Hird , Chem. Soc. Rev. 2007, 36, 2070. 17982522 10.1039/b610738a

[23] G. W. Gray , M. Hird , K. J. Toyne , Mol. Cryst. Liq. Cryst. 1991, 195, 221.

[24] R. J. Mandle , C. J. Gibb , J. L. Hobbs , Liq. Cryst. 2024, 51, 1384.

[25] R. J. Mandle , Soft Matter 2022, 18, 5014. 35776092 10.1039/d2sm00543c

[26] S. T. Lagerwall , P. Rudquist , F. Giesselmann , Mol. Cryst. Liq. Cryst. 2009, 510, 1282.

[27] L. Paik , J. V. Selinger , arXiv [Cond-Mat.Soft] 2024. 10.48550/arXiv.2408.10347.

[28] X. Chen , E. Korblova , M. A. Glaser , J. E. Maclennan , D. M. Walba , N. A. Clark , PNAS 2021, 118, e2104092118. 34050028 10.1073/pnas.2104092118 PMC8179187

[29] P. Kumari , B. Basnet , M. O. Lavrentovich , O. D. Lavrentovich , Science 2024, 383, 1364. 38513040 10.1126/science.adl0834

[30] S. T. Lagerwall , Handbook of Liquid Crystals 2014, pp. 1–258.

[31] N. A. Clark , X. Chen , J. E. MacLennan , M. A. Glaser , Phys. Rev. Res. 2024, 6, 13195.

[32] A. Adaka , M. Rajabi , N. Haputhantrige , S. Sprunt , O. D. Lavrentovich , A. Jákli , Phys. Rev. Lett. 2024, 133, 38101. 10.1103/PhysRevLett.133.038101 39094127

[33] N. Vaupotič , D. Pociecha , P. Rybak , J. Matraszek , M. Čepič , J. M. Wolska , E. Gorecka , Liq. Cryst. 2023, 50, 584.

[34] V. Matko , E. Gorecka , D. Pociecha , J. Matraszek , N. Vaupotič , arXiv [Cond-Mat.Soft] 2024. 10.48550/arXiv.2401.16084.

[35] A. Erkoreka , J. Martinez-Perdiguero , Phys. Rev. E 2024, 110, L022701. 39294943 10.1103/PhysRevE.110.L022701

[36] S. Brown , E. Cruickshank , J. M. D. Storey , C. T. Imrie , D. Pociecha , M. Majewska , A. Makal , E. Gorecka , ChemPhysChem 2021, 22, 2506. 34623724 10.1002/cphc.202100644

[37] H. Matsukizono , Y. Sakamoto , Y. Okumura , H. Kikuchi , J. Phys. Chem. Lett. 2024, 15, 4212. 38599584 10.1021/acs.jpclett.3c03492 PMC11033931

[38] C. J. Gibb , R. J. Mandle , J. Mater. Chem. C Mater. 2023, 11, 16982.

[39] J. Li , Z. Wang , M. Deng , Y. Zhu , X. Zhang , R. Xia , Y. Song , Y. Hisai , S. Aya , M. Huang , Giant 2022, 11, 100109.

[40] E. Cruickshank et al., ACS Omega 2023, 8, 36562. 37810647 10.1021/acsomega.3c05884 PMC10552116

[41] G. J. Strachan , E. Górecka , J. Szydłowska , A. Makal , D. Pociecha , arXiv [Cond-Mat.Soft] 2024. 10.48550/arXiv.2408.07381.

[42] H. Kikuchi , H. Nishikawa , H. Matsukizono , S. Iino , T. Sugiyama , T. Ishioka , Y. Okumura , arXiv [Cond-Mat.Soft] 2024. 10.48550/arXiv.2408.09520. PMC11615755 39439242

