# Supplementary material for: Polar Order in a Fluid Like Ferroelectric with a Tilted Lamellar Structure – Observation of a Polar Smectic C (SmCP) Phase
Source: Angew Chem Int Ed Engl. 2024 Nov 14;64(4):e202416545. doi: 10.1002/anie.202416545 (PMC11753598; doi:10.1002/anie.202416545)
Supplement: Supplementary file 1 — Supporting Information [file ANIE-64-e202416545-s001.pdf]

Angewandte Chemie
Eine Zeitschrift der Gesellschaft Deutscher Chemiker

Supporting Information

Polar Order in a Fluid Like Ferroelectric with a Tilted Lamellar Structure – Observation of a Polar Smectic C (SmC_P) Phase

J. Hobbs, C. J. Gibb, D. Pociecha, J. Szydłowska, E. Górecka, R. J. Mandle*

Supplementary information

Polar order in a fluid like ferroelectric with a titled lamellar structure – observation of a polar smectic C (SmC_P) phase

Jordan Hobbs*[1], Calum J. Gibb[2], Damian Pociecha[3], Jadwiga Szydłowska[3], Ewa Gorecka[3], and Richard. J. Mandle[1,2]

[1] School of Physics and Astronomy, University of Leeds, Leeds, UK, LS2 9JT

[2] School of Chemistry, University of Leeds, Leeds, UK, LS2 9JT

[3] University of Warsaw, Faculty of Chemistry, ul. Zwirki i Wigury 101,02-089, Warsaw, Poland

*Author for correspondence e-mail: j.l.hobbs@leeds.ac.uk

Contents

1 Supplementary methods

2 Supplementary results

3 Organic synthesis

4 Supplementary References

1 Supplementary Methods

1.1. Chemical Synthesis

Chemicals were purchased from commercial suppliers (Fluorochem, Merck, ChemScene, Ambeed) and used as received. Solvents were purchased from Merck and used without further purification. Reactions were performed in standard laboratory glassware at ambient temperature and atmosphere and were monitored by TLC with an appropriate eluent and visualised with 254 nm light. Chromatographic purification was performed using a Combiflash NextGen 300+ System (Teledyne Isco) with a silica gel stationary phase and a hexane/ethyl acetate gradient as the mobile phase, with detection made in the 200-800 nm range. Chromatographed materials were filtered through 200 nm PTFE frits and then subjected to re-crystallisation from an appropriate solvent system. Yields refer to chromatographically and spectroscopically homogenous material.

1.2. Chemical Characterisation

Chemical materials were characterised by NMR spectroscopy using either a Bruker Avance III HDNMR spectrometer operating at 400 MHz, 100.5 MHz or 376.4 MHz (^{1}H, $^{13}C\{^{1}H\}$ and ^{19}F, respectively) or a Bruker AV4 NEO 11.75T spectrometer operating at 500 MHz, 125.5 MHz or 470.5 MHz (^{1}H, $^{13}C\{^{1}H\}$). High resolution mass spectrometry data (HRMS) was collected using a Bruker MaXis Impact spectrometer with a negative ESI source (VIP-HESI); the sample was introduced via direct infusion as solution in EtOH:DMSO (10:1). HPLC analysis was performed using an Agilent 1290 Infinity II system fitted with a poroshell 120 ec-c18 column running a water:MeCN gradient.

1.3. Phase characterisation

Phase transition temperatures and associated enthalpies of transition were determined by differential scanning calorimetry (DSC) using a TA instruments Q2000 heat flux calorimeter with a liquid nitrogen cooling system for temperature control. Samples were measured with 10 ℃ min^{-1} heating and cooling rates. The transition temperatures and enthalpy values reported are averages obtained for duplicate runs. Phase transition temperatures were measured on cooling cycles for consistency between monotropic and enantiotropic phase transitions, while

crystal melts were obtained on heating. Phase identification by polarised optical microscopy (POM) was performed using a Leica DM 2700 P polarised optical microscope equipped with a Linkam TMS 92 heating stage. Samples were studied sandwiched between two untreated glass coverslips.

1.4. Measurement of Spontaneous Polarization (P_S)

Spontaneous polarisation measurements are undertaken using the current reversal technique [44,45]. Triangular waveform AC voltages are applied to the sample cells with an Agilent 33220A signal generator (Keysight Technologies), and the resulting current outflow is passed through a current-to-voltage amplifier and recorded on a RIGOL DHO4204 high-resolution oscilloscope (Telonic Instruments Ltd, UK). Heating and cooling of the samples during these measurements is achieved with an Instec HCS402 hot stage controlled to 10 mK stability by an Instec mK1000 temperature controller. The LC samples are held in 4µm thick cells with no alignment layer, supplied by Instec. The measurements consist of cooling the sample at a rate of 1 K min^{-1} and applying a triangular voltage wave at a frequency of 8 Hz using 10 V_{RMS}.

There are three contributions to the measured current trace: accumulation of charge in the cell (I_c), ion flow (I_i), and the current flow due to polarisation reversal (I_p). To obtain a P_S value, we extract the latter, which manifests as one or multiple peaks in the current flow, and integrate as:

$$P_S = \int \frac{I_p}{2A} dt \qquad \textbf{(2)}$$

where A is the active electrode area of the sample cell.

1.5. X-ray measurements

X-ray diffraction measurements of samples in liquid crystalline phases were carried out using a Bruker D8 GADDS system, equipped with micro-focus-type X-ray source with Cu anode and dedicated optics and VANTEC2000 area detector. Small angle diffraction experiments were performed on a Bruker Nanostar system (IµS microfocus source with copper target, MRI heating stage, Vantec 2000 area detector).

1.6. Optical Birefringence

Optical birefringence was measured with a setup based on a photoelastic modulator (PEM-90, Hinds) working at a modulation frequency f = 50 kHz; as a light source a halogen lamp (Hamamatsu LC8) equipped with narrow bandpass filters was used. The transmitted light intensity was monitored with a photodiode (FLC Electronics PIN20) and the signal was deconvoluted with a lock-in amplifier (EG&G 7265) into 1f and 2f components to yield a retardation induced by the sample. Knowing the sample thickness, the retardation was recalculated into optical birefringence. Samples were prepared in 1.6-µm-thick cells with planar anchoring. The alignment quality was checked prior to measurement by inspection by polarised-light optical microscopy.

1.7 Dielectric studies

The complex dielectric permittivity, ε*, was measured using a Solartron 1260 impedance analyser, in the 1 Hz −10 MHz frequency range, and a probe voltage of 50 mV. The material was placed in a 10-µm-thick glass cell with gold electrodes. Cells without polymer aligning layers were used, as the presence of the thin (~10 nm) polyimide layers at the cell surfaces acts as an additional high capacitance capacitor in a series circuit with the capacitor filled with the LC sample, though this can also occur even without aligning layers, which for materials with very high values of permittivity, may strongly affect the measured permittivity of the LC phases [31]. Lack of a surfactant layer resulted in a random configuration of the director in the LC phases.

Dielectric loss data was fitted using the sum of a power law function for conductivity in the SmA phase, a Cole-Cole function [46] for the dielectric processes observed and power laws to account for any contributions out of range of the frequency window either at the short or long frequency ends of the spectrum. In practice the dielectric response in the SmA phase was fitted using:

$$\varepsilon^* = -\frac{\sigma_{DC}}{\varepsilon_0 \omega} i + \frac{\delta\varepsilon_1}{(1 + (i\omega\tau_1)^{\alpha_1})} + A\omega^n i$$

where σ_{DC} is the DC-conductivity, δε the dielectric strength parameter, τ the characteristic relaxation timescale, α the symmetric broadening parameter (value allowed to vary between 0 and 1), A the amplitude of the long high frequency powerlaw and n a fitting parameter set between 0 and 1.

In the SmA_F and SmC_P phases the data was fitted using:

$$\varepsilon^* = -A\omega^{-n} i + \frac{\delta\varepsilon_2}{(1 + (i\omega\tau_2)^{\alpha_2})} + \frac{\delta\varepsilon_3}{(1 + (i\omega\tau_3)^{\alpha_3})}$$

where the DC conductivity term as being replaced with a general power law to account for the mixture of conductivity and any peak contribution at low frequencies out of the frequency window. Initially, a Havrilliak-Negami function [47] was used to fit the relaxation processes however from the initial fit this was shown to be unnecessary as the β parameter of the Havrilliak-Negami function was shown to be equal to ~1 at all temperatures for all processes and so the Cole-Cole function was used instead. Representative fits are shown in Fig. S13

1.8 SHG Microscopy

The SHG response was investigated using a microscopic setup based on a solid-state laser EKSPLA NL202. Laser pulses (9 ns) at a 10 Hz repetition rate and max. 2 mJ pulse energy at λ= 1064 nm were applied. The pulse energy was adjusted for each sample to avoid its decomposition. The infra-red beam was incident onto a LC homogenous cell of thickness 5 µm. An IR pass filter was placed at the entrance to the sample and a green pass filter at the exit of the sample.

1.9 Molecular modelling

1.9.1 Electronic Structure Calculations

All electronic structure calculations were performed using the Gaussian G16 software package, revision c01 [48]. Geometry optimisation was performed using the B3LYP hybrid functional [49,50], the cc-pVTZ basis set [51] and GD3BJ dispersion correction [52]. Following geometry optimisation, a frequency calculation at the same level was used to confirm the geometry to be at a minimum.

Molecular length and width are calculated by projecting the atoms into a single plane along a specific molecular axis. The length and width is then calculated by finding the pair of atoms furthest apart in that projected plane. Dipole angle is calculated by finding the angle between the dipole vector and the long molecular axis through the rigid core.

1.9.2 Molecular Dynamics

The initial geometry for **PPZGU-3-F** (n=3) was obtained by optimisation (at the B3LYP-GD3BJ/cc-pVTZ level of DFT, as above). RESP charges were calculated at the HF/6-31G* level [53] using Gaussian G16.c01 with the route section “iop(6/33=2) iop(6/42=6) pop(chelpg,regular)“. Using Antechamber (part of AmberTools24/Amber 24 software packages [54]) the Gaussian output file was converted to .mol2 format; finally, this was parsed

into Gromacs readable .itp format using the ACPYPE script [55]. Simulations were performed in Gromacs 2022.2 using GPU acceleration (Nvidia V100s) *via* CUDA 12.2.1; bonded, non-bonded, PME and update tasks were offloaded to the GPU during the production run.

Initial coordinates were generated *pseudo* randomly using the Gromacs tool *gmx insert-molecules*; we construct a low-density (~ 0.1 g cm^3) initial configuration consisting of 1000 molecules with random positional and orientational order. To this we then perform energy minimisation *via* steepest descent, followed by equilibration in the NVE and NVT regimes (the later at T=600K). A brief (20 ns) compression simulation with an isotropic barostat (*P* = 100 Bar) at T=600K affords a liquid-like density isotropic state, used as a starting configuration for subsequent simulations. In the case of polar simulations, a static electric field was applied across the x-axis of the simulation for 50 ns (at T=600K) to give a near saturated polar order parameter (<*P*1> > 0.95); this was used as a starting configuration for polar simulations. In all cases the production run was >300 ns total duration.

Simulations employed periodic boundary conditions in xyz. Bonds lengths were constrained to their equilibrium values with the LINCS algorithm [56]. Compressabilities in xyz dimensions were set to 4.5e-5, with the off-diagonal compressibilities were set to zero to ensure the simulation box remained rectangular. Long-range electrostatic interactions were calculated using the Particle Mesh Ewald method with a cut-off value of 1.2 nm. A van der Waals cut-off of 1.2 nm was used.

Analysis was conducted on each trajectory frame, and presented values are given as the average over all frames in a given time window (typically the entire production run), with uncertainties given as one standard deviation from the mean.

A simulation was deemed liquid crystalline if the second-rank orientational order parameter (<*P*2>), calculated *via* the Q-tensor approach using MdTraj v1.9.8 [57], was greater than 0.3 (and judged to be isotropic if below this value). Phase type was confirmed by visual inspection of the trajectory. The polar order parameter (<*P*1>) is found as the quotient of the total simulation dipole moment and the maximum possible dipole moment of the simulation. The spontaneous polarization was calculated as the ratio of the net dipole moment of the system to the simulation box volume.

In some simulations, (smectic) layer order spontaneously emerges. We measure the layer spacing and associated tilt by principal component analysis (PCA). We represent each molecule as its centre-of-mass (COM) and in doing so provide a consistent 3D coordinate for each analysis. PCA was then applied to the COM data directly, allowing the principal axes of variation in the molecular positions to be identified. By projecting the first principal component (PC1) onto a single axis, the layers are identifiable as clusters of points along said axis; thus

the layer spacing is the difference between adjacent clusters. The angle between the PC1 vector (normal to the layer planes) and the director affords the tilt angle. The method detailed here has the advantage of being insensitive to the position of specific atoms and/or conformational fluctuations during the simulation.

Simulated 2D X-ray scattering patterns were computed from the simulation trajectory by modelling the electron density of each atom in the simulation as a Gaussian sphere (using atomic radii from Bondi; [58]. For all atoms in a given frame, the atomic electron density is mapped onto a 3D grid; Fourier transform of this density grid gives the 3D structure factor. This process loops over all frames and the structure factor of the trajectory is the sum of all frames. Finally, summation of the structure factor along a given direction gives a 2D scattering pattern (e.g. summation along the [1] index corresponds to the Y-axis, thus yields the SAXS pattern in the XZ plane), following correction for the Ewald sphere. The code used for simulation of 2D SAXS patterns is available from GitHub (https://www.github.com/RichardMandle/simusaxs).

2 Supplementary Results

Table S1 Transition temperatures (℃) and associated enthalpy changes (KJ mol^{-1}) for the **PPZGU-*n*-F** series.[ ] brackets indicate monotropic transitions

n	**m.p.**		**SmC$_P$-SmA$_F$**		**SmA$_F$-SmA**		**SmA-N**		**N-I**	
	T / ℃	ΔH / KJ mol^{-1}	T / ℃	ΔH / KJ mol^{-1}	T / ℃	ΔH / KJ mol^{-1}	T / ℃	ΔH / KJ mol^{-1}	T / ℃	ΔH / KJ mol^{-1}
**2**	154.3	23.8	[152.1]	0.03	[158.2]	0.3	177.0	0.5	249.1	0.5
**3**	132.0	23.8	[118.8]	0.06	141.4	0.9	186.2	0.5	249.8	0.7
**4**	105.4	21.4	[106.3]	0.04	[109.9]	0.2	195.6	0.9	237.9	0.5
**5**	100.9	20.4	-	-	-	-	204.1	1.1	236.1	0.7
**7**	79.3	18.4	-	-	-	-	210.2	1.4	224.3	0.6
**9**	72.4	27.0	-	-	-	-	210.3	1.8	214.1	0.6

Table S2 Calculated DFT parameters; dipole angle refers to the angle between the dipole vector and the molecular long axis.

n	Dipole moment (μ) / D	Dipole Angle (θ_μ) / °	Molecular length (L) / Å	Molecular Width (W) / Å	Molecular Aspect Ratio (Ar)
**2**	6.95	11.3	22.28	5.13	4.34
**3**	6.99	10.7	23.26	5.17	4.50
**4**	7.09	11.8	24.57	5.14	4.78
**5**	7.07	11.8	25.58	5.15	4.96
**7**	7.10	13.5	27.95	5.30	5.27
**9**	7.12	15.3	30.36	5.97	5.07

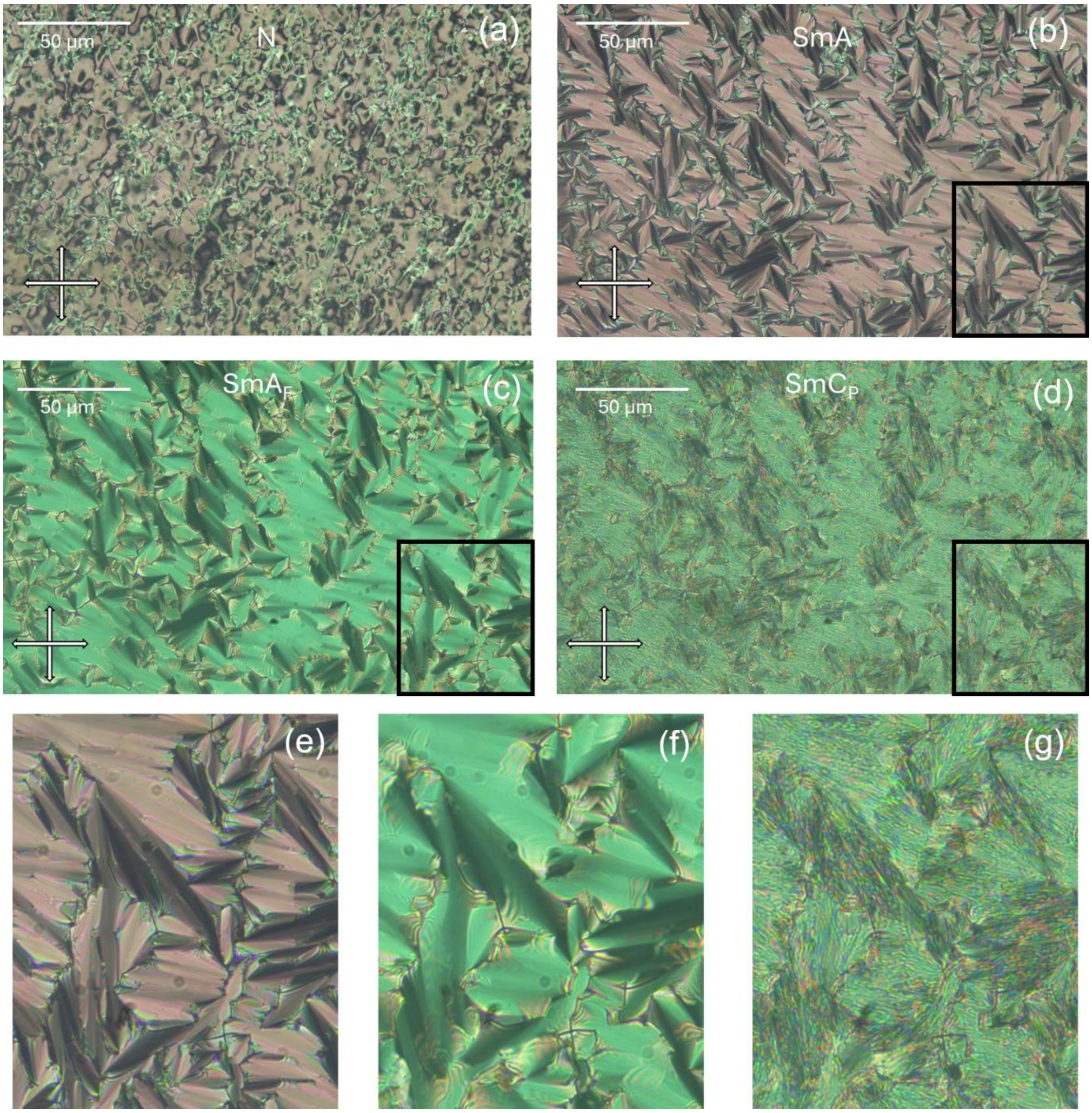

Fig. S1. POM micrographs of the (a) N (190 ºC), (b) SmA, (175 ºC) (c) SmA_F (130 ºC) and (d) SmC_P (110 ºC) phases observed for **PPZGU-3-F** within a thin cell treated for homeotropic anchoring. The bottom row show the same black boxed region of the SmA phase in (a) for the (e) SmA, (f) SmA_F and (g) SmC_P phases

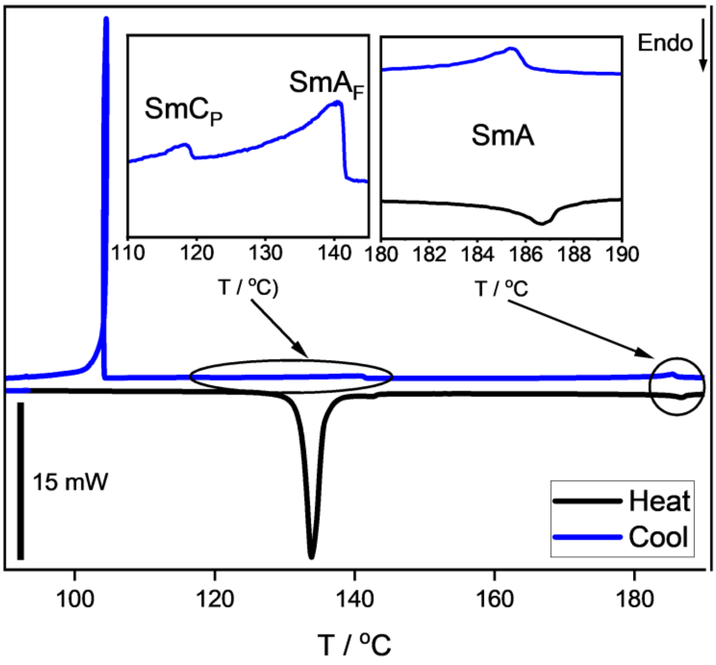

Fig. S2. Example DSC thermogram for **PPZGU-3-F** showing first cooling cycle and second heating cycle. Samples were measured with 10 K min^{-1} cooling rate. The N-Iso transition has been omitted for clarity.

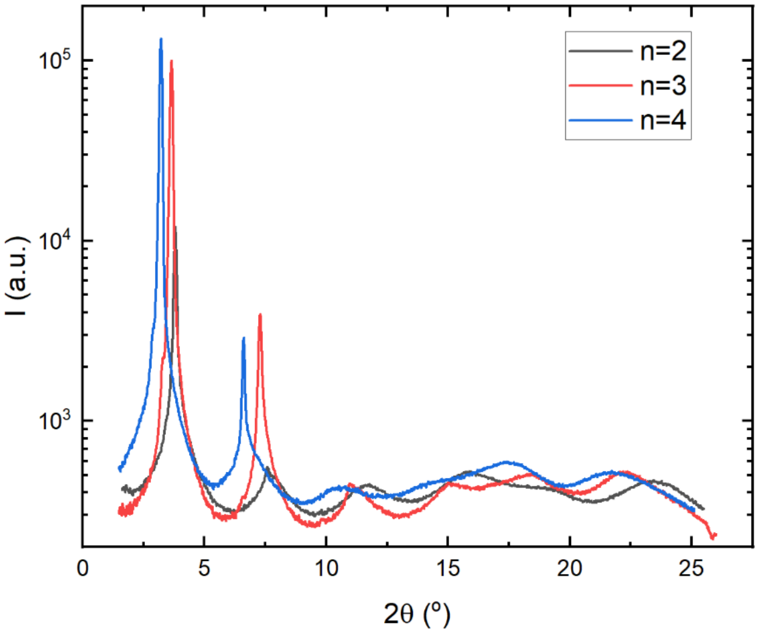

Fig. S3. X-ray scattering data for the polar materials showing the series of commensurate signals corresponding to layer spacing. Radial integration was

done in a radial sector around layer normal direction, that exclude any wide-angle scattering.

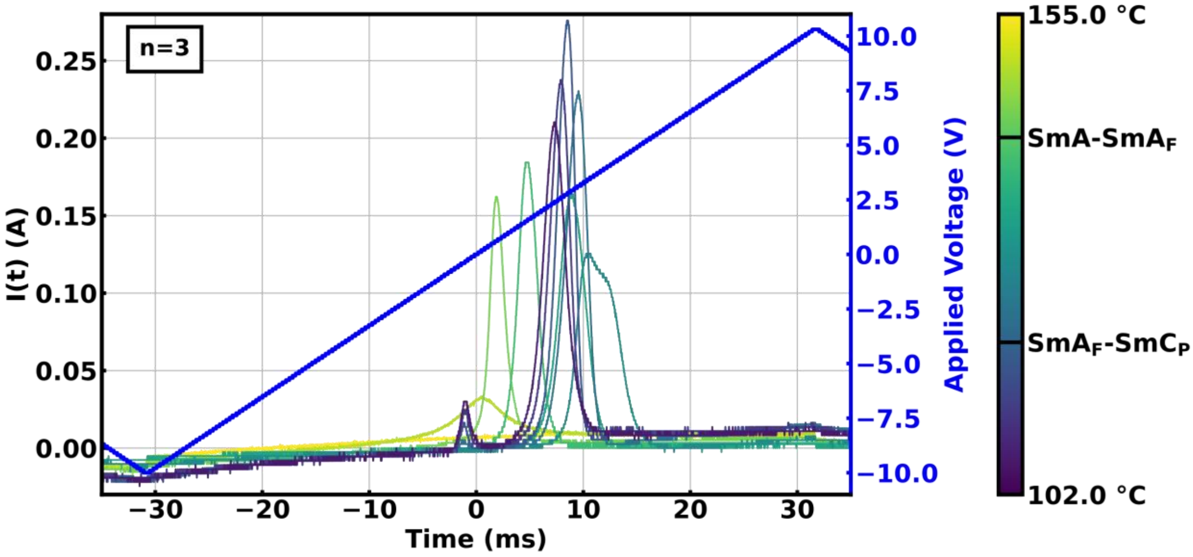

Fig. S4. Current response traces, measured at 8 Hz, as function of temperature for **PPZGU-3-F.**

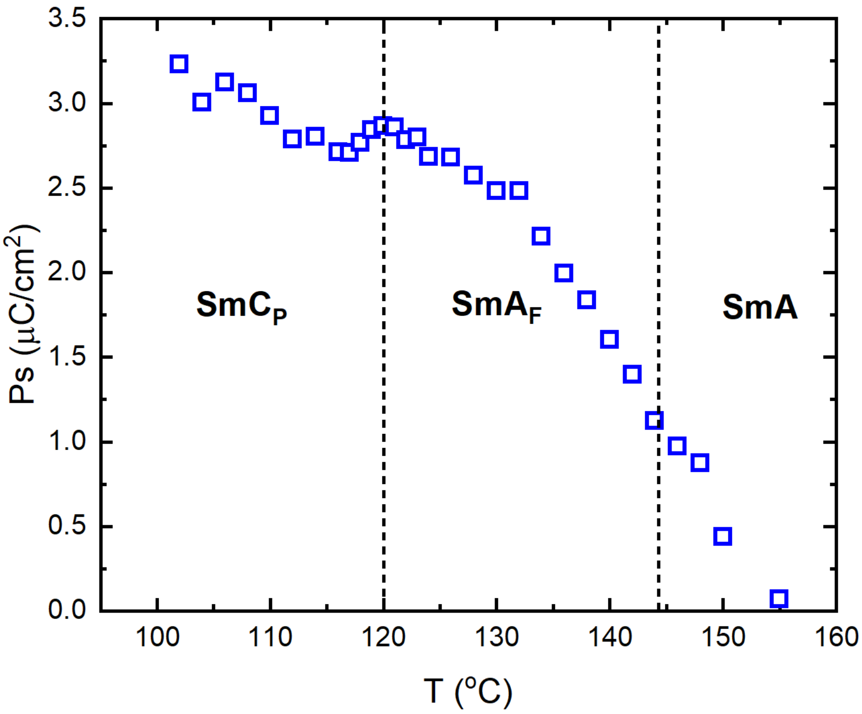

Fig. S5 Temperature dependence of the spontaneous polarization (Ps) measured at 8 Hz in the SmA, SmA$_F$ and SmC$_P$ phases for **PPZGU-3-F**; the pre-transitional P_S measured in the SmA is field-induced polarisation.

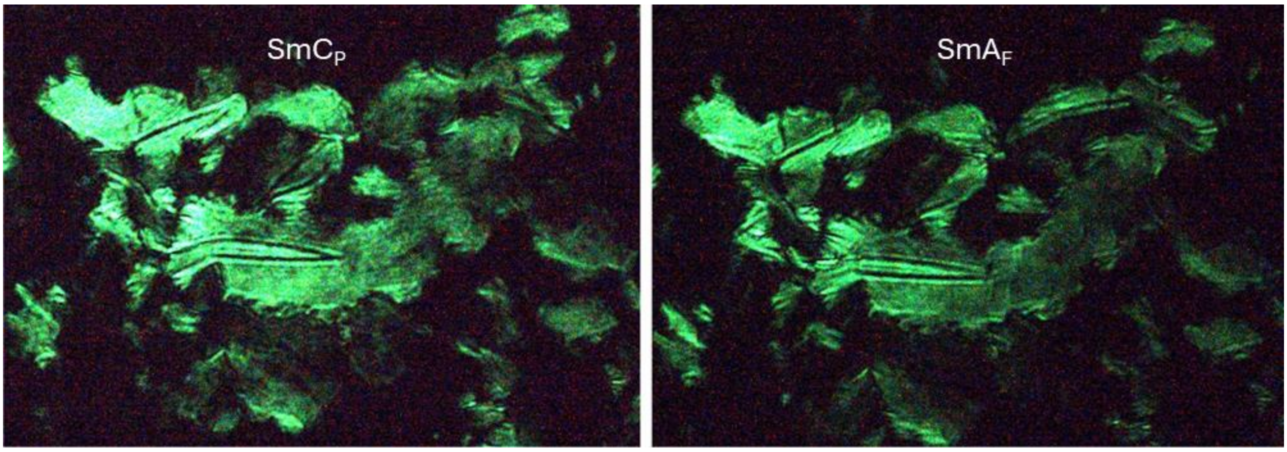

Fig. S6. SGH microscopy images of the SmC$_P$ [left] and SmA$_F$ [right] phases observed for **PPZGU-3-F**. The images are taken without an applied electrical field. The bright domains are those with polarization perpendicular the light propagation direction.

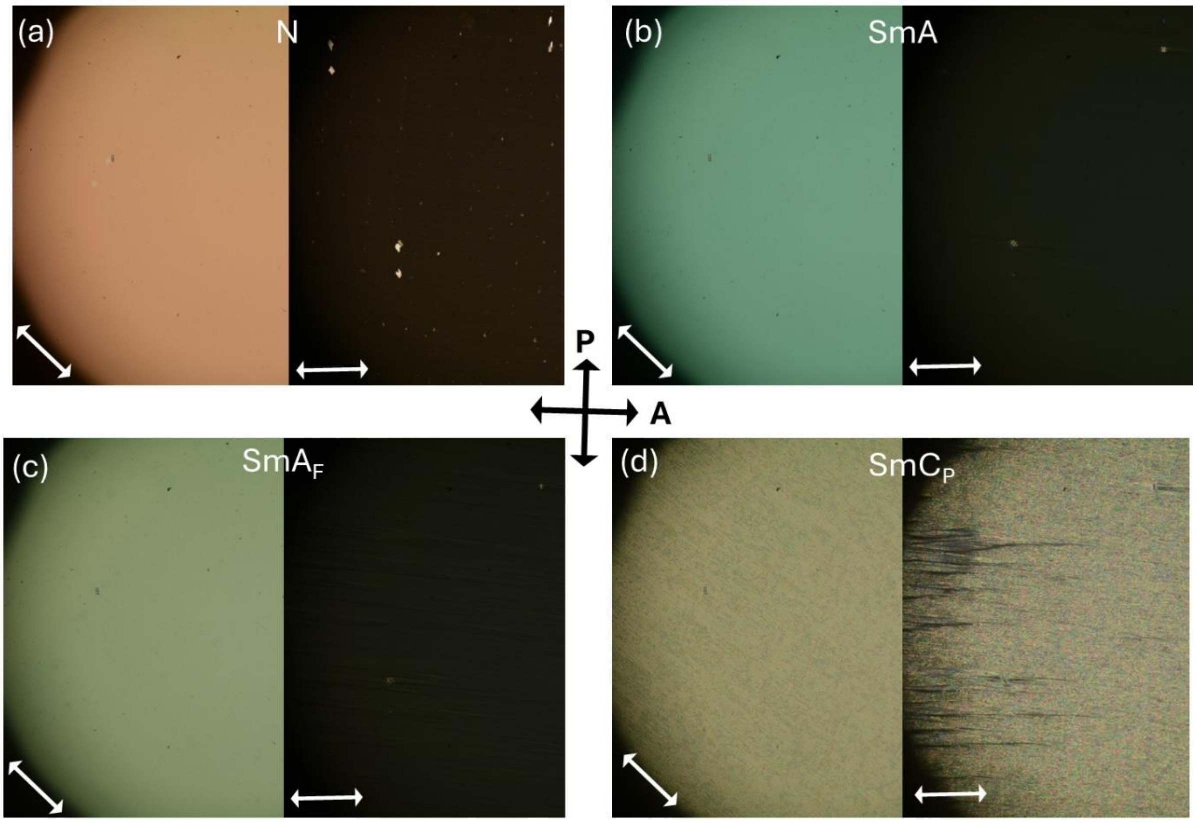

Fig. S7. POM micrographs comparing the light and dark states of the (a) N, (b) SmA, (c) SmA$_F$, and (d) SmC$_P$ phases. **PPZGU-3-F** samples are confined in thin cells treated for a planar anchoring condition with anti-parallel rubbing. “P” and “A”

arrows indicate polariser and analyser directions respectively. White arrow on POM images indicates the rubbing direction.

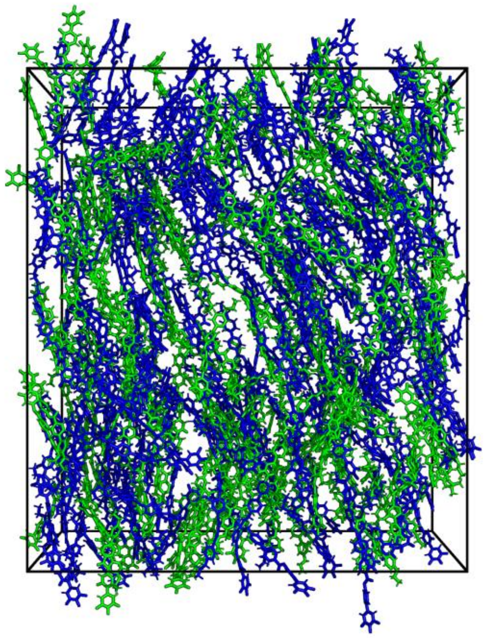

Fig. S8. Instantaneous configurations of: (a) the *apolar* nematic phase of **n=3** that forms spontaneously from the isotropic liquid at T 475 K at 78 ns (of a production run of >300 ns with average <*P*2> = 0.61 +/- 0.04). The snapshot is color-coded green/blue to show molecules whose dipole is parallel or antiparallel with the director, respectively (<*P*1> = 0.03 +/- 0.01). Only 400 molecules (out of 1000) are shown, to aid visualisation.

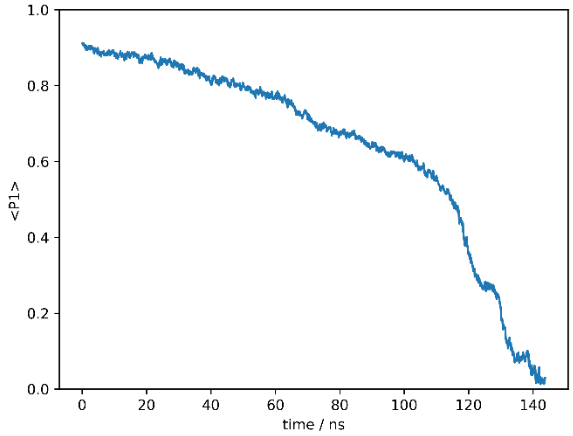

Fig. S9. The decay of the polar order parameter (<P1>) of PPZGU-3-F (n=3) when beginning from a field induced polar nematic configuration at T = 500 K. After

removal of the field, <P1> decays to zero by t = 140 ns, at which point the simulation is in a non-polar nematic configuration (<P2> = 0.50.

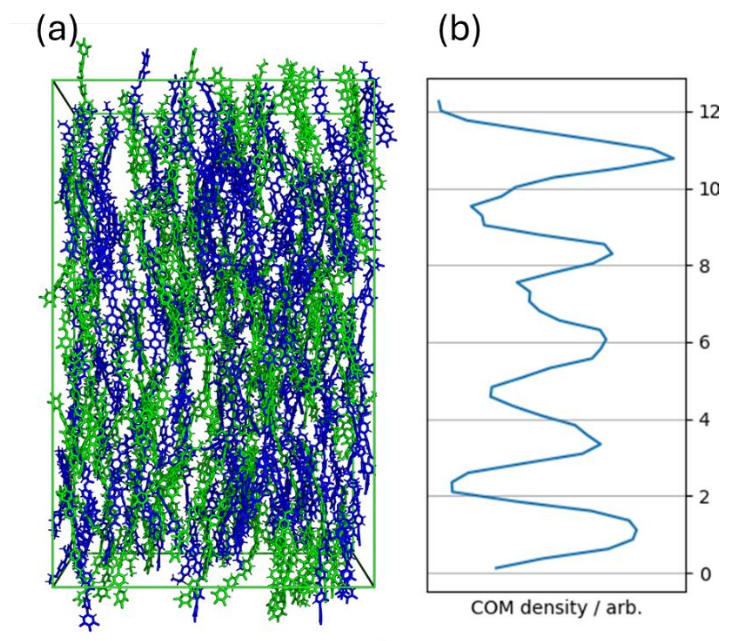

Fig. S10. (a) Instantaneous configuration of the *apolar* smectic A phase of PPZGU-3-F (n=3) formed spontaneously from the isotropic liquid at T 425 K at 350 ns (of a production run of >500 ns, <*P*2> = 0.81 +/- 0.08). The snapshot is color-coded green/blue to show molecules whose dipole is parallel or antiparallel with the director, respectively (<*P*1> = 0.04 +/- 0.02). The layer spacing is 2.4 +/- 0.1 nm. Only 400 molecules (out of 1000) are shown, to aid visualisation. (b) Plot of the density of molecular centres-of-mass along a vector normal to the layer plane shows the diffuse layer structure.

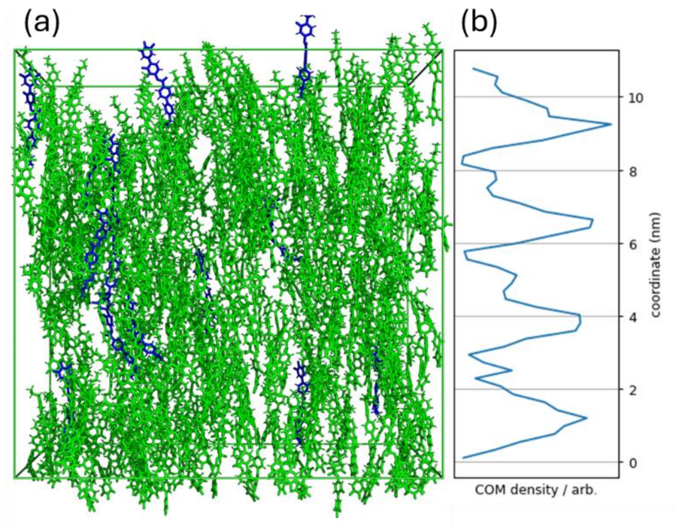

Fig S11. (a) Instantaneous configuration of the *polar* smectic A phase of PPZGU-3-F (n=3) formed spontaneously from the polar initial configuration at T 475 K at 259 ns (of a production run of >500 ns, <*P*2> = 0.72 +/- 0.03). The snapshot is color-coded green/blue to show molecules whose dipole is parallel or antiparallel with the director, respectively (<*P*1> = 0.88 +/- 0.03). The hydrocarbon chains are omitted from the rendering to aid visualisation of the layer structure (layer spacing = 2.3 +/- 0.1 nm; The calculated spontaneous polarisation (P_S) is 2.5 +/- 0.02 µC.m^2. Only 400 molecules (out of 1000) are shown, to aid visualisation. (b) Plot of the density of molecular centres-of-mass along the vector normal to the layer plane shows the diffuse layer structure

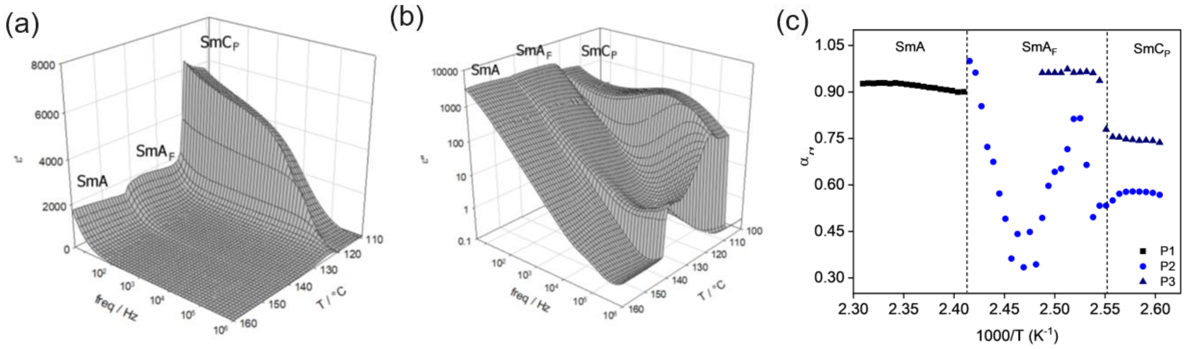

Fig S12. (a) Real and (b) imaginary permittivity data obtained for PPZGU-3-F in a 10 µm cell with untreated gold electrodes. (c) The α parameter obtained from fitting the dielectric loss data.

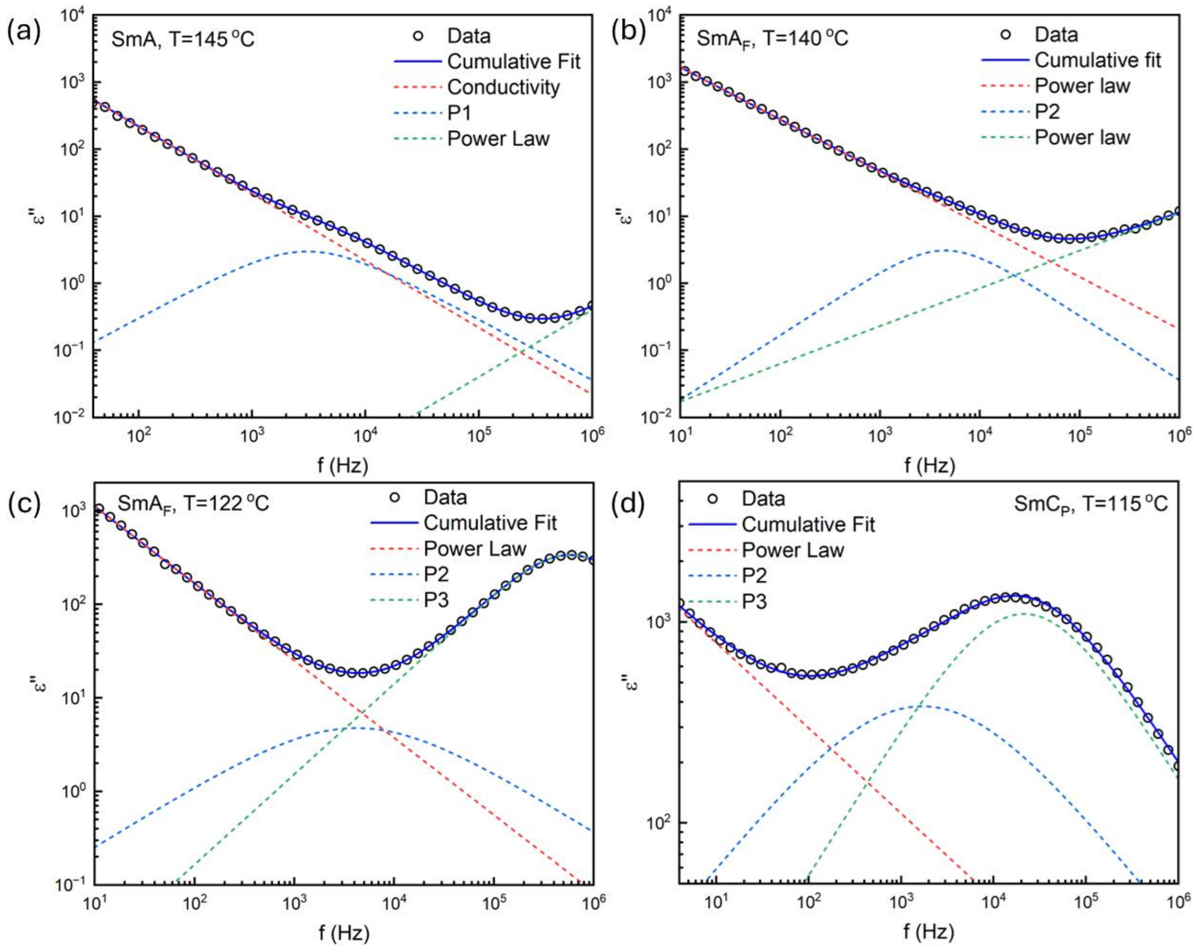

Fig S13. Example fits of the dielectric relaxation data obtained for PPZGU-3-F at (a) 145 °C, (b) 140 °C, (c) 122 °C and (d) 115 °C.

3 Organic synthesis

3.1 General Esterification Protocol

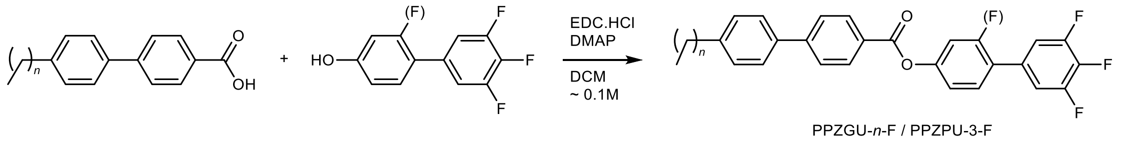

Scheme 1. General esterification protocol used in the preparation of the **PPZGU-*n*-F** and **PPZPU-3-F** materials.

The carboxylic acid (1.5 mmol), phenol (4-hydroxy-2,3',4',5'-tetrafluorobiphenyl; 1 mmol), EDC.HCl (1.5 mmol), DMAP (5 mg) were weighed into a reaction vial or round bottom flask. DCM was added (~ 10 ml, final concentration w.r.t. phenol ~ 0.1 M), and the resulting suspension stirred until complete consumption of the starting phenol as judged by TLC. The crude reaction mixture was purified by flash chromatography using a 12 g SiO_2 cartridge as the stationary phase and a gradient of hexane/EtOAc (0% EtOAc - 25% EtOAc) as the mobile phase, with detection made in the range 200-800 nm. The chromatographed material was filtered through a 200 nm PTFE syringe filter, concentrated to dryness, and finally recrystalised from ethanol.

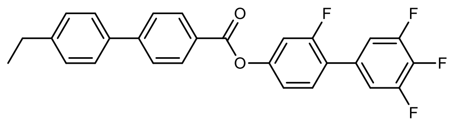

1 (**PPZGU-2-F**): 3-fluoro-4-(3,4,5-trifluorophenyl)phenyl 4-(4-ethylphenyl)benzoate

Yield: (white crystals) 360 mg, 80%

R_F (DCM): 0.76

^{1}H NMR (400 MHz, $CDCl_3$) (δ): 8.26 (d (apparent), J = 8.4 Hz, 2H, Ar-**H**), 7.75 (d (apparent), J = 8.5 Hz, 2H, Ar-**H**), 7.60 (d (apparent), J = 8.2 Hz, 2H, Ar-**H**), 7.44 (t, J = 8.6 Hz, 1H, Ar-**H**), 7.34 (d (apparent), J = 8.1 Hz, 2H, Ar-**H**), 7.24 – 7.11 (m, 4H, Ar-**H**), 2.74 (q, J = 7.6 Hz, 2H, Ar-C**H$_2$**-CH$_3$), 1.31 (t, J = 7.6 Hz, 3H, CH$_2$-C**H$_3$**).
^{13}C{^{1}H} NMR (101 MHz, $CDCl_3$) (δ): 164.63, 159.43 (d, J = 250.9 Hz), 151.82 (d, J = 11.1 Hz), 151.61 (ddd, J = 235.7, 9.6, 3.7 Hz), 146.77, 144.90, 139.50 (dt, J = 252.9, 16.0 Hz), 130.82, 130.60 (d, J = 4.0 Hz), 128.61, 127.28, 127.21, 127.13, 123.84 (d, J = 12.9 Hz), 118.35, 118.31, 113.69 – 112.89 (m), 110.79 (d, J = 25.7 Hz), 28.61, 15.55.

^{19}F NMR (376 MHz, $CDCl_3$) (δ): -114.61 (t $_{(apparent)}$, J_{H-F} = 9.7 Hz, 1F, Ar-**F**), -134.21 (dd, J_{F-F} = 20.6, J_{H-F} = 8.9 Hz, 2F, Ar-**F**), -161.28 (tt, J_{F-F} = 20.8, J_{H-F} = 6.6 Hz, 1F, Ar-**F**).

HPLC (C18): 97.81% (254 nm, 6.063 min)

HRMS (ESI -, m/z) = $[m-H]^+$: Calculated for $C_{27}H_{17}F_4O_2$: 449.1170, found 449.1171 (error = -0.1 ppm)

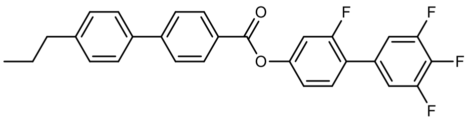

2 (**PPZGU-3-F**): 3-fluoro-4-(3,4,5-trifluorophenyl)phenyl 4-(4-propylphenyl)benzoate

Yield: (white crystals) 357 mg, 77%

R_F (DCM): 0.76

^1H NMR (400 MHz, $CDCl_3$) (δ): 8.25 (d $_{(apparent)}$, J = 8.7 Hz, 2H, Ar-**H**), 7.75 (d $_{(apparent)}$, J = 8.7 Hz, 2H, Ar-**H**), 7.62 – 7.56 (m, 2H, Ar-**H**), 7.44 (t, J = 8.7 Hz, 1H, Ar-**H**), 7.31 (d $_{(apparent)}$, J = 8.3 Hz, 2H, Ar-**H**), 7.20 (d $_{(apparent)}$, J = 8.7 Hz, 2H, Ar-**H**), 7.16 (t, J = 2.2 Hz, 1H, Ar-**H**), 7.15 – 7.12 (m, 1H, Ar-**H**), 2.66 (t, J = 7.5 Hz, 2H, Ar-C**H₂**-CH₂), 1.70 (h, J = 7.4 Hz, 2H, CH_2-C**H₂**-CH_3), 0.99 (t, J = 7.3 Hz, 3H, CH_2-C**H₃**).

$^{13}C\{^1H\}$ NMR (126 MHz, $CDCl_3$) (δ): 164.64, 160.43, 158.43, 151.82 (d, J = 11.0 Hz), 150.18 (ddd, J = 235.8, 9.7, 4.3 Hz), 146.78, 143.36, 139.50 (dt, J = 252.9, 15.2 Hz), 137.00, 130.82, 130.60 (d, J = 3.9 Hz), 129.20, 127.18, 127.12, 123.84 (d, J = 13.0 Hz), 118.35, 118.32, 113.52 – 112.87 (m), 110.79 (d, J = 25.8 Hz), 37.75, 24.54, 13.88.

^{19}F NMR (376 MHz, $CDCl_3$) (δ): -114.62 (t $_{(apparent)}$, J_{H-F} = 9.7 Hz, 1F, Ar-**F**), -134.23 (dd, J_{F-F} = 20.6, J_{H-F}= 8.6 Hz, 2F, Ar-**F**), -161.29 (tt, J_{F-F} = 20.7, J_{H-F} = 6.6 Hz, 1F, Ar-**F**).

HPLC (C18): 99.20% (254 nm, 6.269 min)

HRMS (ESI -, m/z) = $[m-H]^+$: Calculated for $C_{28}H_{19}F_4O_2$: 463.1327, found 463.1345 (error = 4.0 ppm)

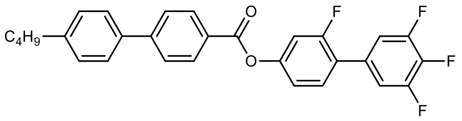

3 (**PPZGU-4-F**): 3-fluoro-4-(3,4,5-trifluorophenyl)phenyl 4-(4-butylphenyl)benzoate

Yield: (white fluffy needles) 391 mg, 82%

R_F (DCM): 0.79

^{1}H NMR (501 MHz, $CDCl_3$) (δ) : 8.26 (d $_{(apparent)}$, J = 8.2 Hz, 2H, Ar-**H**), 7.75 (d $_{(apparent)}$, J = 8.3 Hz, 2H, Ar-**H**), 7.60 (d $_{(apparent)}$, J = 8.1 Hz, 2H, Ar-**H**), 7.43 (t, J = 8.7 Hz, 1H, Ar-**H**), 7.33 (d $_{(apparent)}$, J = 7.9 Hz, 2H, Ar-**H**), 7.23 – 7.12 (m, 4H, Ar-**H**), 2.71 (t, J = 7.8 Hz, 2H, Ar-C**H**$_2$-CH_2), 1.68 (p, J = 7.8 Hz, 2H, CH_2-C**H**$_2$-CH_2), 1.44 (h, J = 7.5 Hz, 2H, CH_2-C**H**$_2$-CH_3), 1.00 (t, J = 7.4 Hz, 3H, CH_2-C**H**$_3$).

^{13}C{^{1}H} NMR (126 MHz, $CDCl_3$) (δ): 164.60, 159.43 (d, J = 250.8 Hz), 151.86 (d, J = 11.0 Hz), 150.20 (ddd, J = 235.3, 9.8, 4.1 Hz), 146.75, 143.61, 139.50 (dt, J = 253.1, 14.8 Hz), 136.92, 130.82, 130.57 (d, J = 3.8 Hz), 129.17, 127.19, 127.09, 123.78 (d, J = 12.9 Hz), 118.36, 118.33, 113.51 – 112.89 (m), 110.79 (d, J = 25.8 Hz), 35.40, 33.62, 22.46, 14.00.

^{19}F NMR (376 MHz, $CDCl_3$) (δ): -114.60 (t $_{(apparent)}$, J_{H-F} = 9.7 Hz, 1F, Ar-**F**), -133.44 – -134.91 (m $_{(apparent)}$, 2F, Ar-**F**), -161.27 (tt, J_{F-F} = 20.3, 6.6 Hz, 1F, Ar-**F**).

HPLC (C18): 99.50% (254 nm, 6.506 min)

HRMS (ESI -, m/z) = $[m-H]^+$: Calculated for $C_{29}H_{21}F_4O$: 477.1483, found 477.1482 (error = 0.2 ppm)

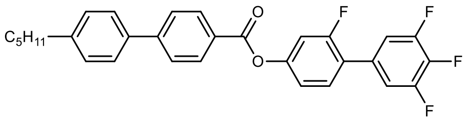

4 (**PPZGU-5-F**): 3-fluoro-4-(3,4,5-trifluorophenyl)phenyl 4-(4-pentylphenyl)benzoate

Yield: (white crystals) 364 mg, 74%

R_F (DCM): 0.80

^{1}H NMR (400 MHz, $CDCl_3$) (δ): 8.25 (d $_{(apparent)}$, J = 8.4 Hz, 2H, Ar-**H**), 7.75 (d $_{(apparent)}$, J = 8.5 Hz, 2H, Ar-**H**), 7.59 (d $_{(apparent)}$, J = 8.0 Hz, 2H, Ar-**H**), 7.44 (t, J = 8.7 Hz, 1H, Ar-**H**), 7.31 (d $_{(apparent)}$, J = 8.1 Hz, 2H, Ar-**H**), 7.23 – 7.12 (m, 3H, Ar-**H**), 2.68 (t, J = 7.3 Hz, 2H, Ar-C**H**$_2$-CH_2), 1.67 (p, J = 7.5 Hz, 2H, CH_2-C**H**$_2$-CH_2), 1.44 – 1.30 (m, 4H, CH_2-(C**H**$_2$)$_2$-CH_2), 0.92 (t, J = 6.7 Hz, 3H, CH_2-C**H**$_3$).

$^{13}C\{^1H\}$ NMR (101 MHz, $CDCl_3$) (δ): 164.64, 159.43 (d, J = 251.0 Hz), 151.82 (d, J = 11.1 Hz), 151.22 (ddd, J = 249.7, 5.6, 3.9 Hz), 146.78, 143.63, 139.50 (dt, J = 253.1, 14.8 Hz), 136.96, 130.81, 130.60 (d, J = 4.0 Hz), 129.14, 127.19, 127.12, 123.78 (d, J = 12.5 Hz), 118.35, 118.31, 113.41 – 113.01 (m), 110.79 (d, J = 25.7 Hz), 35.65, 31.54, 31.14, 22.57, 14.05.

^{19}F NMR (376 MHz, $CDCl_3$) (δ): -114.62 (t $_{(apparent)}$, $J_{H\text{-}F}$ = 10.0 Hz, 1F Ar-**F**), -134.22 (dd, $J_{F\text{-}F}$ = 20.6, $J_{H\text{-}F}$ 8.6 Hz, 2F, Ar-**F**), -161.29 (tt, $J_{F\text{-}F}$ = 20.6, $J_{H\text{-}F}$ = 6.6 Hz, 1F, Ar-**F**).

HPLC (C18): 99.5% (254 nm, 6.796 min)

HRMS (ESI -, m/z) = $[m\text{-}H]^+$: Calculated for $C_{30}H_{23}F_4O_2$: 491.1640, found 491.1634 (error = 1.2 ppm)

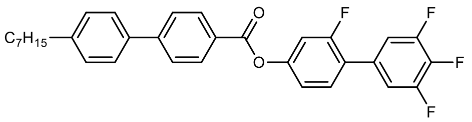

5 (**PPZGU-7-F**): 3-fluoro-4-(3,4,5-trifluorophenyl)phenyl 4-(4-heptylphenyl)benzoate

Yield: (white solid) 406 mg, 78%

R_F (DCM): 0.80

^1H NMR (400 MHz, $CDCl_3$) (δ): 8.25 (d $_{(apparent)}$, J = 8.5 Hz, 2H, Ar-**H**), 7.75 (d $_{(apparent)}$, J = 8.5 Hz, 2H, Ar-**H**), 7.59 (d $_{(apparent)}$, J = 8.0 Hz, 2H, Ar-**H**), 7.44 (t, J = 8.7 Hz, 1H, Ar-**H**), 7.31 (d $_{(apparent)}$, J = 8.2 Hz, 2H, Ar-**H**), 7.24 – 7.11 (m, 4H, Ar-**H**), 2.68 (t, J = 7.5 Hz, 2H, Ar-C$\mathbf{H_2}$-CH_2), 1.67 (p, J = 7.7 Hz, 2H, CH_2-C$\mathbf{H_2}$-CH_2), 1.45 – 1.24 (m, 8H, CH2-(C$\mathbf{H_2}$)$_4$-CH_2), 0.90 (t, J = 6.8 Hz, 3H, CH_2-C$\mathbf{H_3}$).

$^{13}C\{^1H\}$ NMR(101 MHz, $CDCl_3$) (δ): 164.64, 159.43 (d, J = 250.9 Hz), 151.82 (d, J = 11.0 Hz), 149.98 (ddd, J = 243.9, 6.4, 3.9 Hz), 146.78, 143.64, 138.24 (dt, J = 253.7, 15.4 Hz), 136.94, 130.59 (d, J = 4.0 Hz), 130.57, 129.14, 127.19, 127.11, 123.84 (d, J = 13.6 Hz), 118.35, 118.31, 113.56 – 112.93 (m), 110.79 (d, J = 25.8 Hz), 35.69, 31.85, 31.48, 29.33, 29.21, 22.70, 14.13.

^{19}F NMR (376 MHz, $CDCl_3$) (δ): -114.61 (t $_{(apparent)}$, $J_{H\text{-}F}$ = 9.8 Hz, 1F, Ar-**F**), -134.21 (dd, $J_{F\text{-}F}$ = 20.7, $J_{H\text{-}F}$ = 8.6 Hz, 2F, Ar-**F**), -161.28 (tt, $J_{F\text{-}F}$ = 20.6, $J_{H\text{-}F}$ = 6.5 Hz, 1F Ar-**F**).

HPLC (C18): 98.37% (254 nm, 7.680 min)

HRMS (ESI -, m/z) = [m-H]$^+$: Calculated for $C_{32}H_{27}F_4O_2$: 519.1953, found 519.1973 (error = -3.8 ppm)

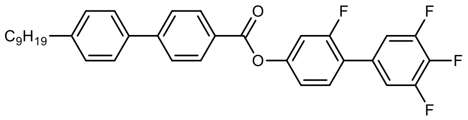

6 (**PPZGU-9-F**): 3-fluoro-4-(3,4,5-trifluorophenyl)phenyl 4-(4-nonylphenyl)benzoate

Yield: (white solid) 384 mg, 70%

R_F (DCM): 0.81

^{1}H NMR (400 MHz, $CDCl_3$) (δ): 8.26 (d (apparent), J = 8.2 Hz, 2H, Ar-**H**), 7.75 (d (apparent), J = 8.1 Hz, 2H, Ar-**H**), 7.59 (d (apparent), J = 7.8 Hz, 2H, Ar-**H**), 7.44 (t, J = 8.7 Hz, 1H, Ar-**H**), 7.32 (d (apparent), J = 7.8 Hz, 2H, Ar-**H**), 7.24 – 7.12 (m, 4H, Ar-**H**), 2.69 (t, J = 7.7 Hz, 2H, Ar-C**H₂**-CH₂), 1.68 (p, J = 7.4 Hz, 2H, CH₂-C**H₂**-CH₂), 1.46 – 1.21 (m, 14H, CH₂-(C**H₂**)₇-CH₂), 0.91 (t, J = 6.6 Hz, 3H, CH₂-C**H₃**).

^{13}C{^{1}H} NMR (101 MHz, $CDCl_3$) (δ): 164.62, 159.43 (d, J = 251.0 Hz), 151.84 (d, J = 11.0 Hz), 149.96 (ddd, J = 235.9, 10.0, 4.2 Hz), 146.78, 143.65, 138.24 (dt, J = 252.8, 15.2 Hz), 136.94, 130.81, 130.59 (d, J = 4.0 Hz), 129.14, 127.19, 127.11, 123.82 (d, J = 13.0 Hz), 118.35, 118.31, 113.52 – 112.85 (m), 110.79 (d, J = 25.8 Hz), 35.70, 31.94, 31.48, 29.60, 29.57, 29.38*, 22.72, 14.15.

*overlapping aliphatic signal, corresponds to two aliphatic carbon atoms.

^{19}F NMR (376 MHz, $CDCl_3$) (δ): -114.59 (t (apparent), J_{H-F} = 9.8 Hz, 1F, Ar-**F**), -134.18 (dd, J_{F-F} = 20.6, J_{H-F} = 8.9 Hz, 2F, Ar-**F**), -161.25 (tt, J_{F-F} = 20.9, J_{H-F} = 6.6 Hz, 1F Ar-**F**).

HPLC (C18): 99.00% (254 nm, 9.174 min)

HRMS (ESI -, m/z) = [m-H]$^+$: Calculated for $C_{34}H_{31}F_4O_2$: 547.2266, found 547.2282 (error = -3.0 ppm)

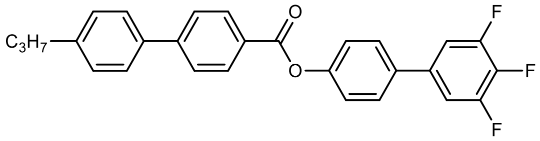

7 (PPZPU-3-F): 4-(3,4,5-trifluorophenyl)phenyl 4-(4-propylphenyl)benzoate

Yield: (white solid) 336 mg, 75%

R_F (DCM): 0.77

^{1}H NMR (501 MHz, $CDCl_3$) (δ): 8.27 (d $_{(apparent)}$, J = 8.4 Hz, 2H, Ar-**H**), 7.75 (d $_{(apparent)}$, J = 8.4 Hz, 2H, Ar-**H**), 7.60 (d $_{(apparent)}$, J = 8.4 Hz, 1H, Ar-**H**), 7.56 (d $_{(apparent)}$, J = 8.7 Hz, 2H, Ar-**H**), 7.36 – 7.29 (m, 4H, Ar-**H**), 7.24 – 7.16 (m, 2H, Ar-**H**), 2.67 (t, J = 6.8 Hz, 2H, Ar-C**H₂**-CH₂), 1.71 (h, J = 7.5 Hz, 2H, CH_2-C$\mathbf{H_2}$-CH_3), 1.00 (t, J = 7.3 Hz, 3H, CH_2-C$\mathbf{H_3}$).

^{13}C{^{1}H} NMR (126 MHz, $CDCl_3$) (δ): 165.05, 152.22 (ddd, J = 235.4, 5.6, 4.2 Hz), 152.41, 146.54, 143.28, 139.34 (td, J = 252.1, 15.5 Hz), 137.09, 136.81 – 136.29 (m), 135.95, 132.55, 130.77, 129.19, 128.05, 127.62, 127.18, 127.07, 122.50, 111.12 (dd, J = 11.2, 5.4 Hz), 37.75, 24.55, 13.89.

19F NMR (376 MHz, $CDCl_3$) δ -133.93 (dd, J_{F-F} = 20.6, J_{F-H} = 8.7 Hz, 2F, Ar-**F**), -162.47 (tt, J_{F-F} = 20.6, J_{H-F} = 6.5 Hz, 1F, Ar-**F**).

HPLC (C18): 99.22% (254 nm, 6.182 min)

HRMS (ESI -, m/z) = $[m-H]^+$: Calculated for $C_{28}H_{20}F_3O_2$: 445.1421, found 445.1434 (error = -2.9 ppm)

3.2 Example NMR Spectra, HPLC Chromatograms, and HRMS Spectra.

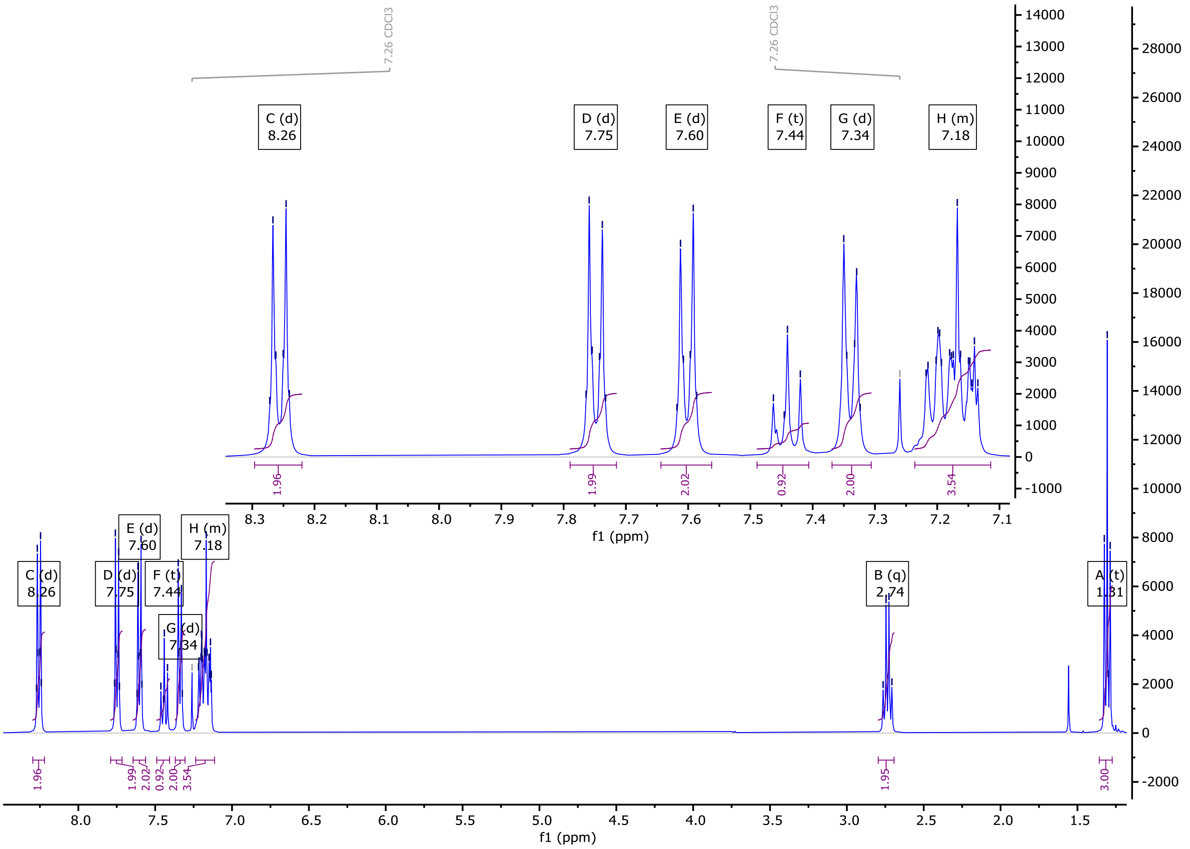

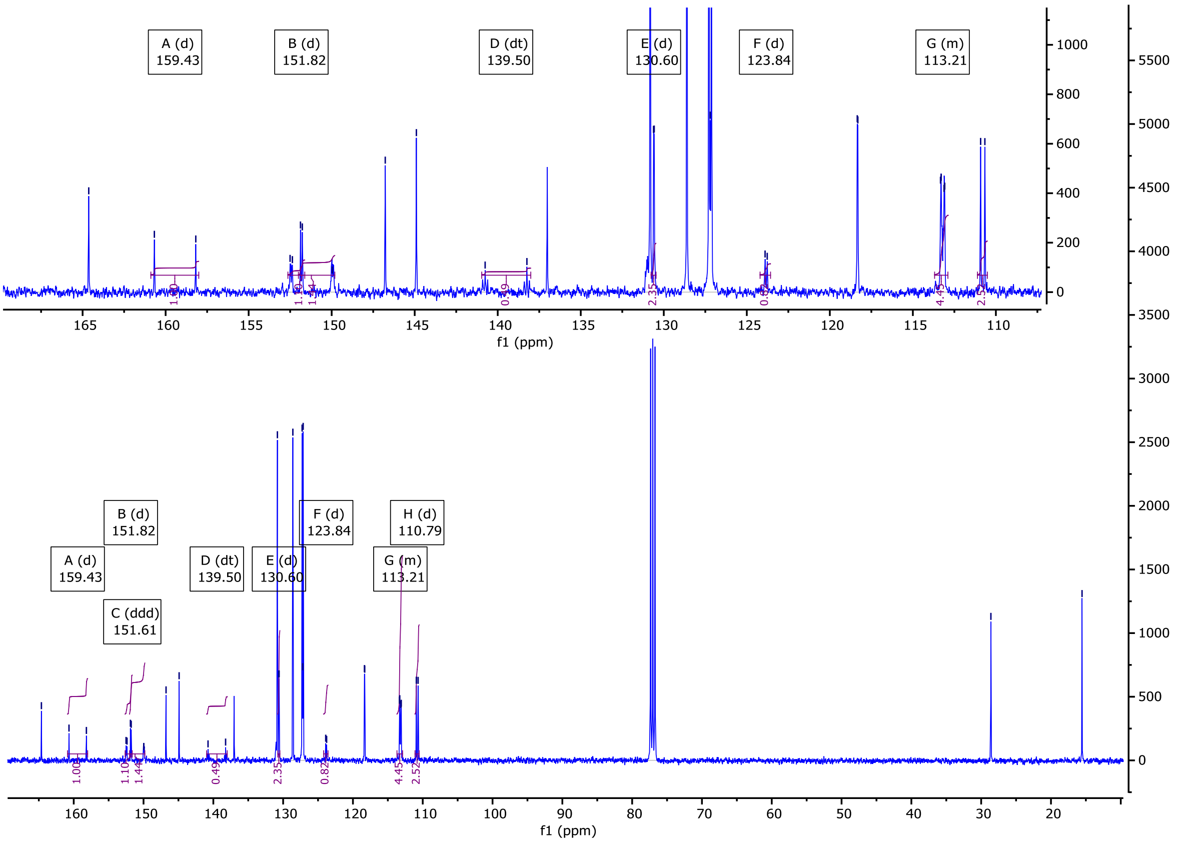

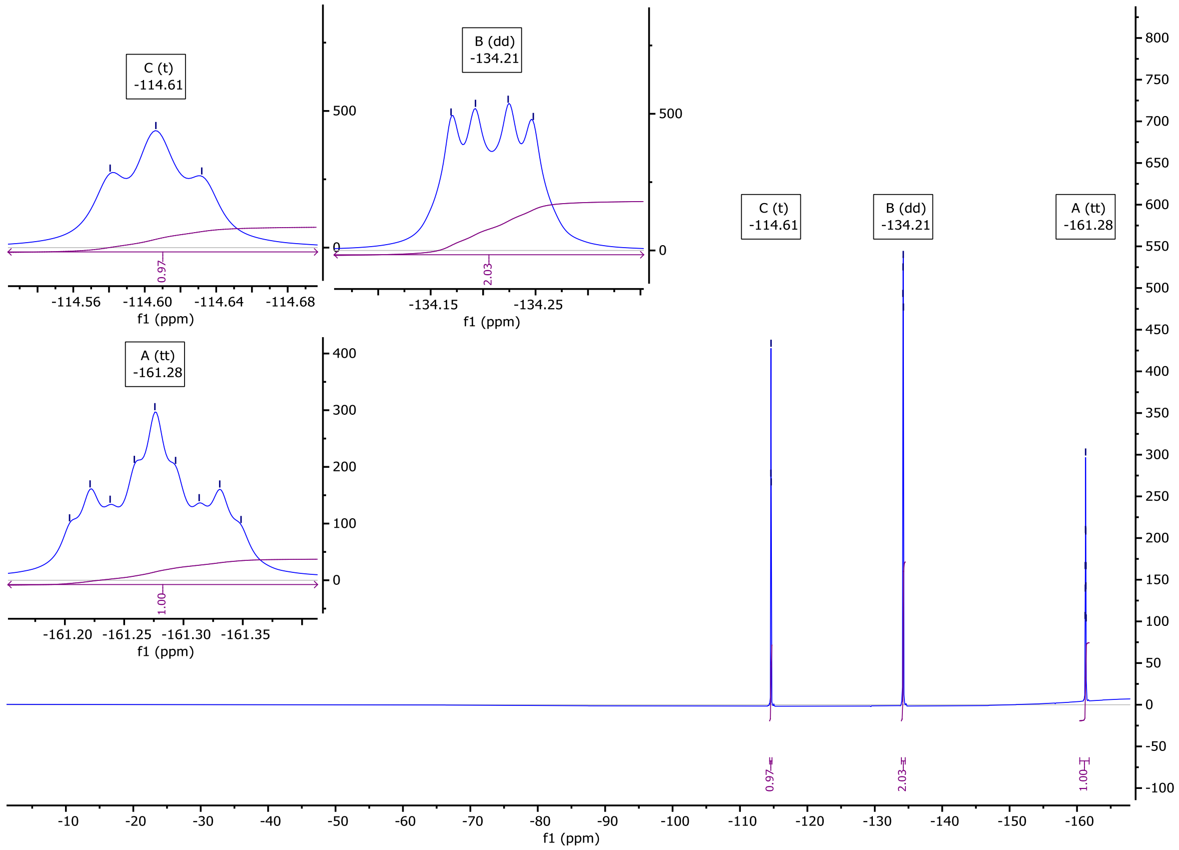
C (t)
-114.61
B (dd)
-134.21
A (tt)
-161.28
f1 (ppm)

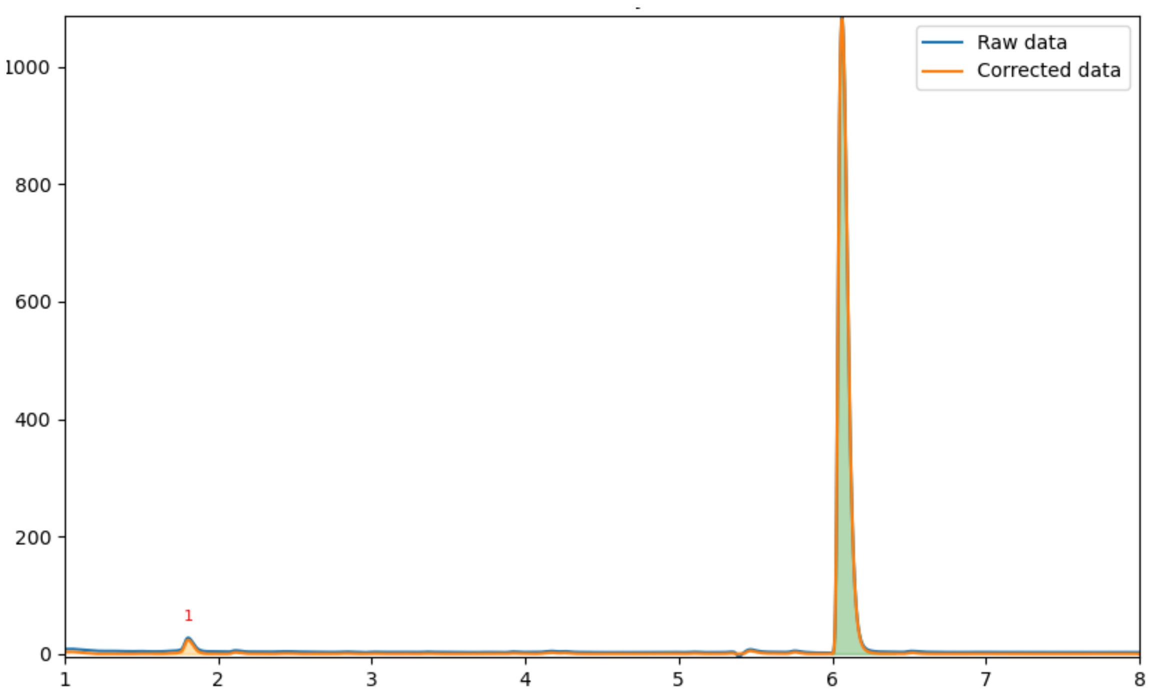
Raw data
Corrected data
1

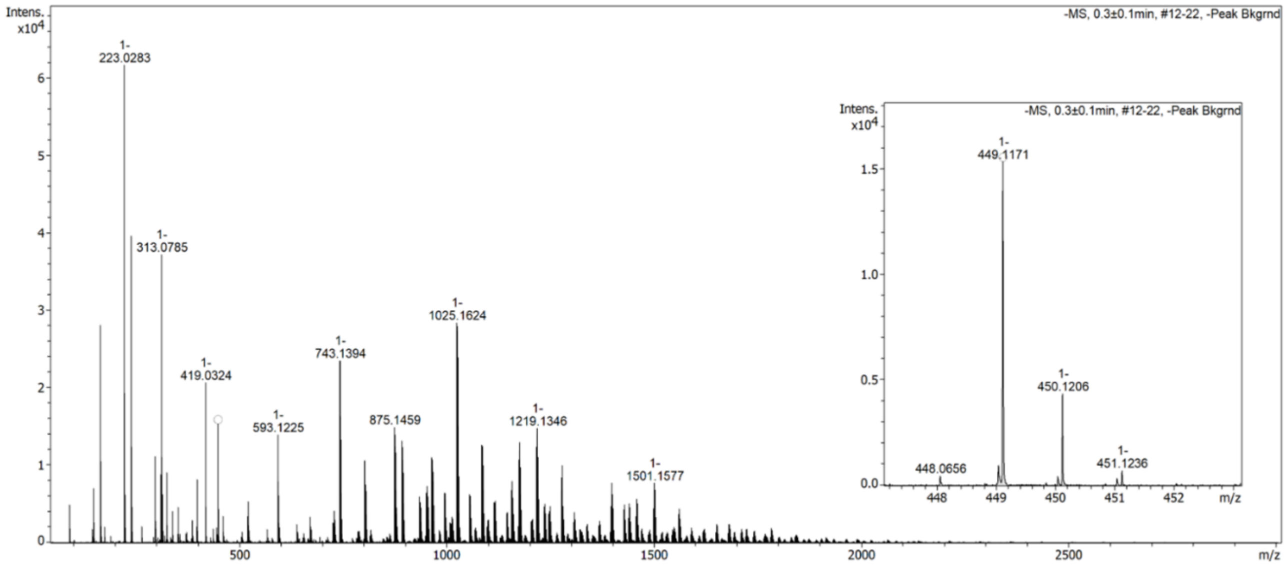

Fig. S14. ^{1}H [top], $^{13}C\{^{1}H\}$ [middle], and ^{19}F [bottom] NMR spectra, HPLC chromatogram, and HRMS Spectra for **1** (**PPZGU-2-F**).

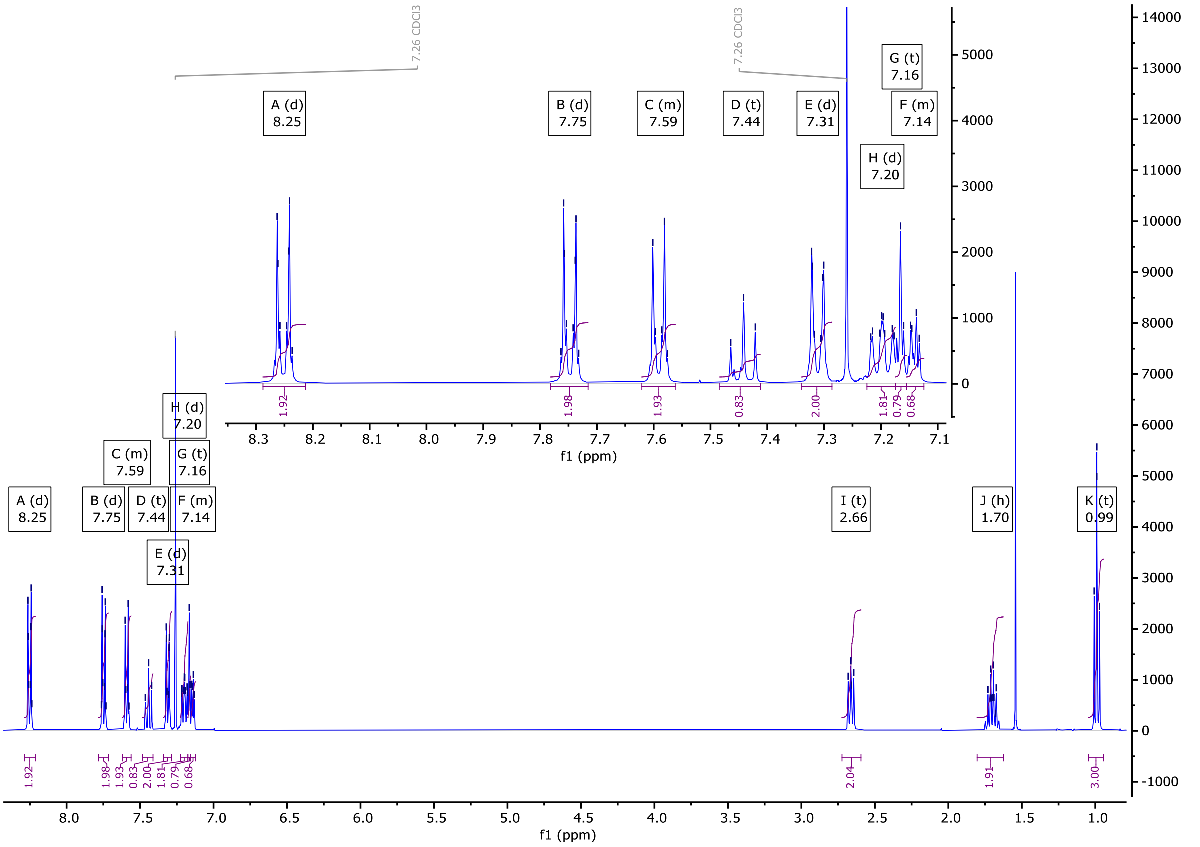
A (d) 8.25
B (d) 7.75
C (m) 7.59
D (t) 7.44
E (d) 7.31
H (d) 7.20
G (t) 7.16
F (m) 7.14
I (t) 2.66
J (h) 1.70
K (t) 0.99
7.26 CDCl3
f1 (ppm)

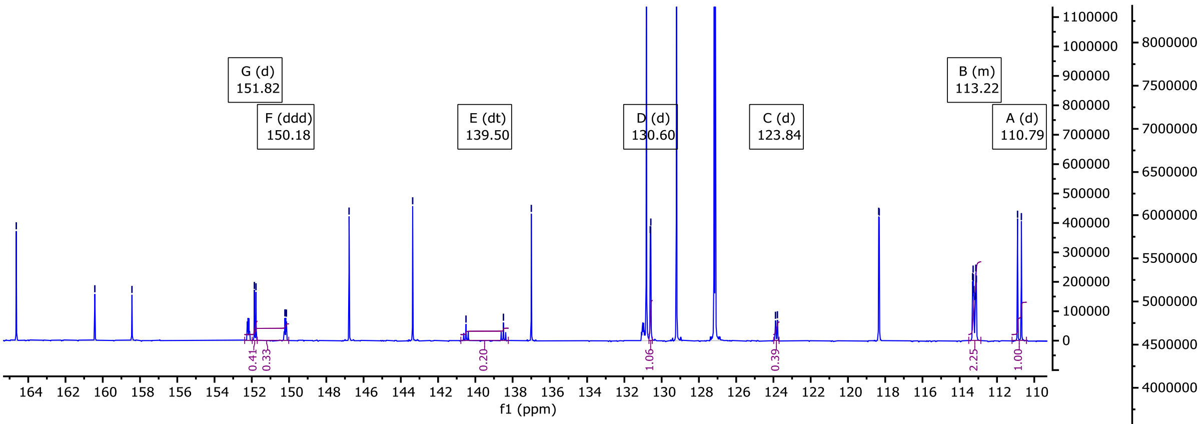
G (d) 151.82
F (ddd) 150.18
E (dt) 139.50
D (d) 130.60
C (d) 123.84
B (m) 113.22
A (d) 110.79
f1 (ppm)

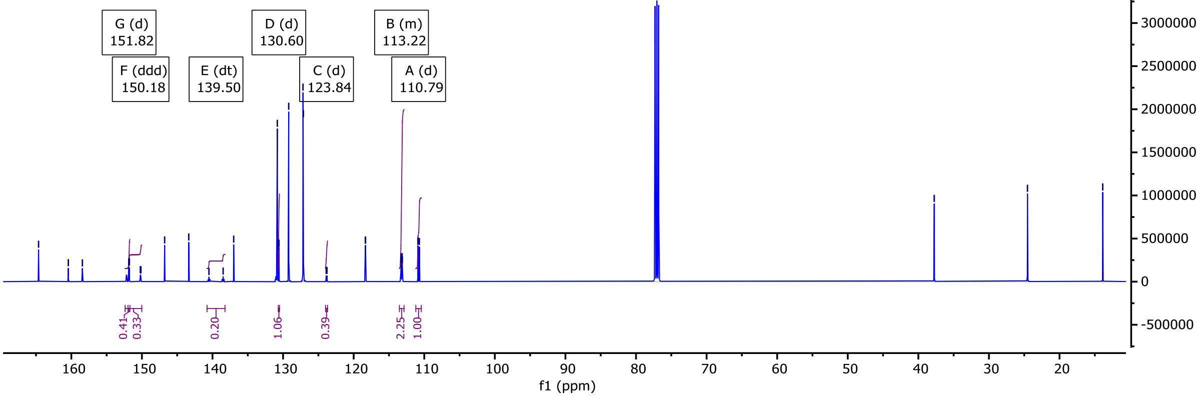
G (d) 151.82
F (ddd) 150.18
E (dt) 139.50
D (d) 130.60
C (d) 123.84
B (m) 113.22
A (d) 110.79
f1 (ppm)

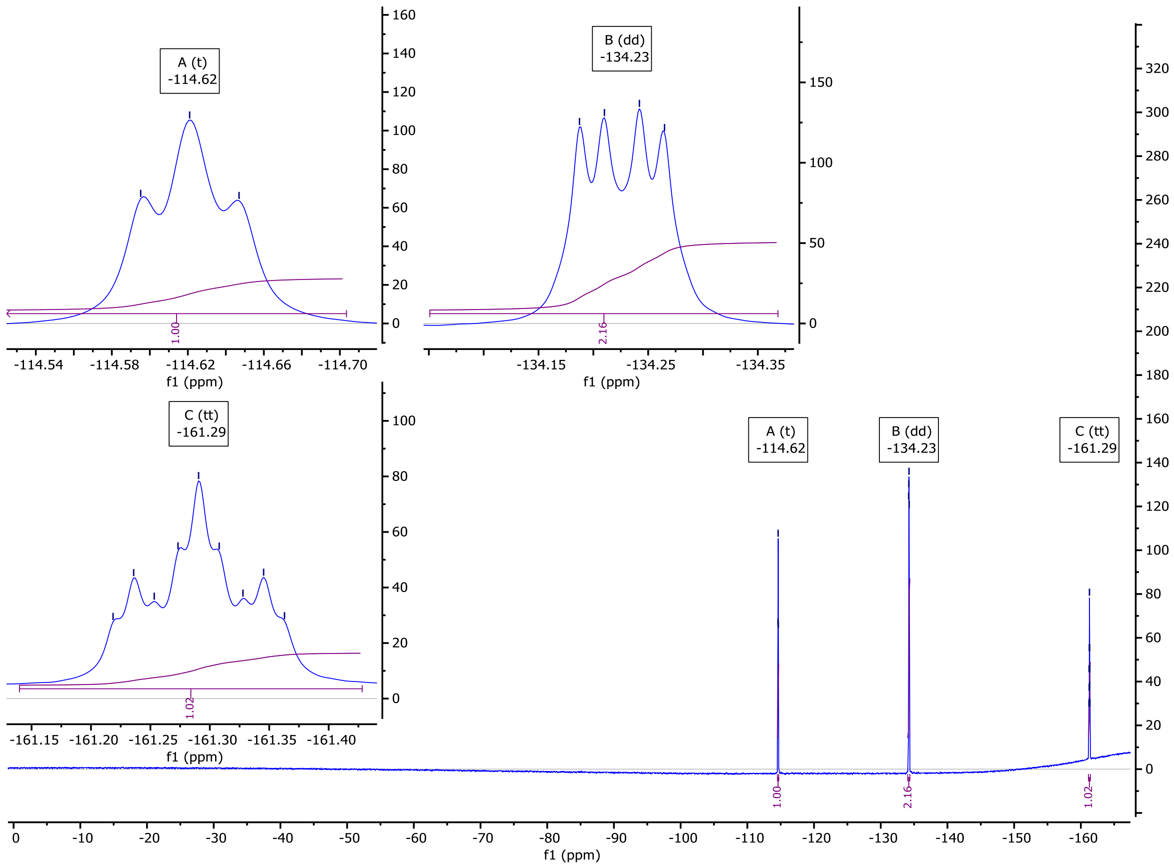
A (t)
-114.62
B (dd)
-134.23
C (tt)
-161.29
1.00
2.16
1.02
f1 (ppm)

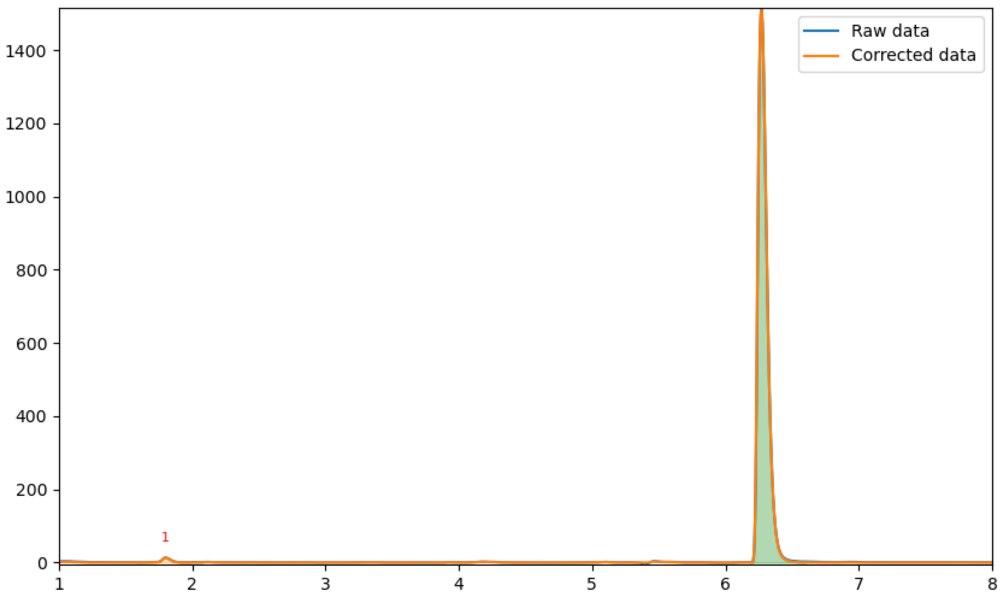
Raw data
Corrected data
1

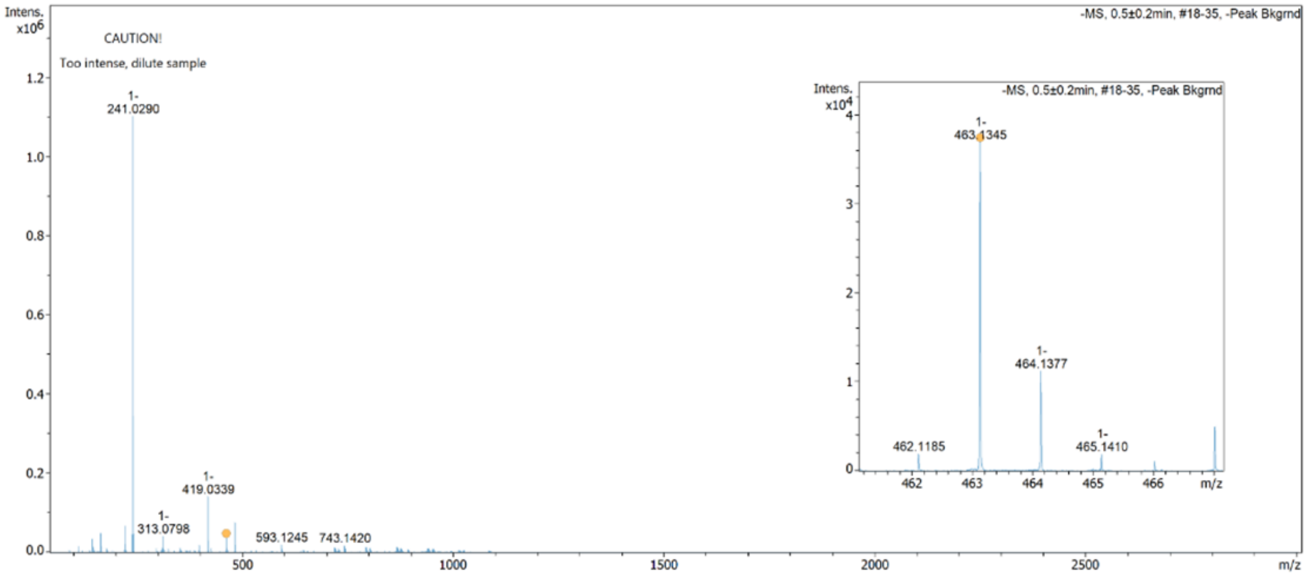

Fig. S15. ^{1}H [top], $^{13}C\{^{1}H\}$ [middle], and ^{19}F NMR spectra, HPLC chromatogram, and HRMS Spectra for **2** (**PPZGU-3-F**).

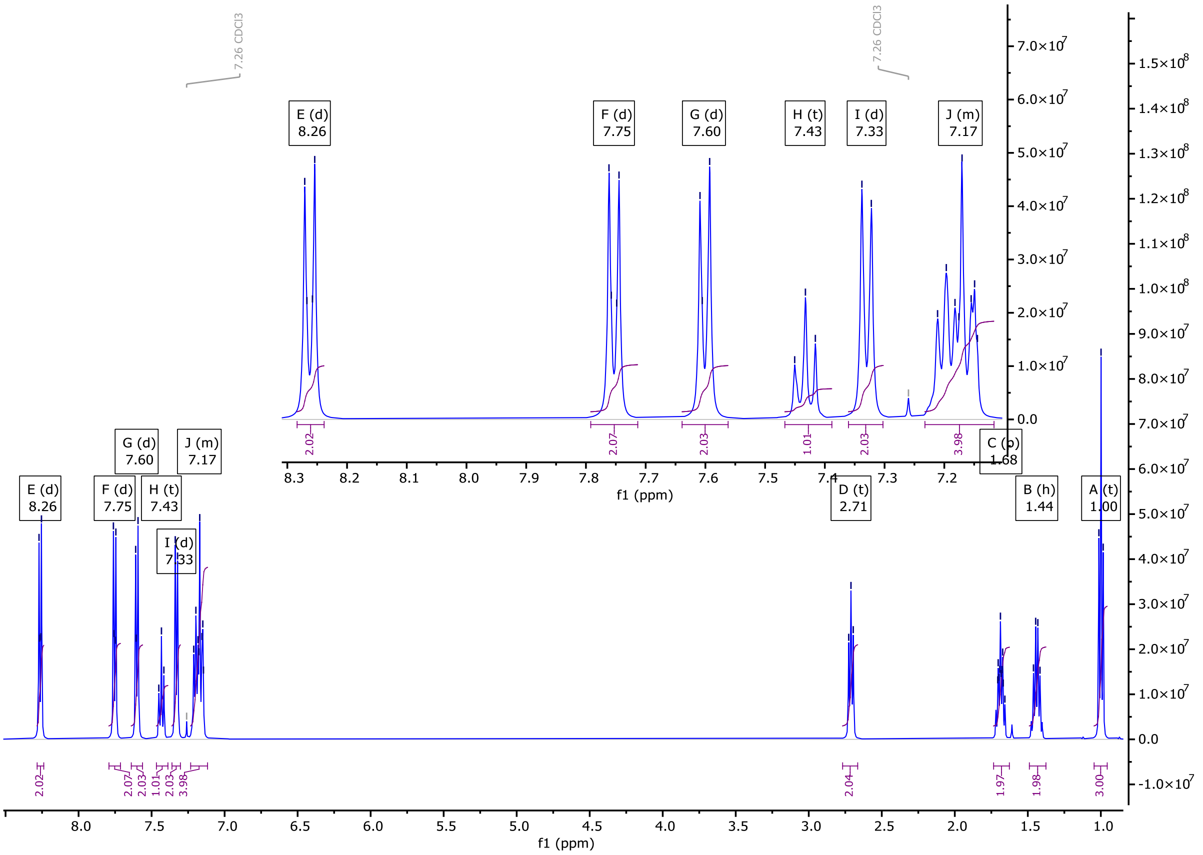
7.26 CDCl3
E (d) 8.26
F (d) 7.75
G (d) 7.60
H (t) 7.43
I (d) 7.33
J (m) 7.17
C (p) 1.68
D (t) 2.71
B (h) 1.44
A (t) 1.00
f1 (ppm)

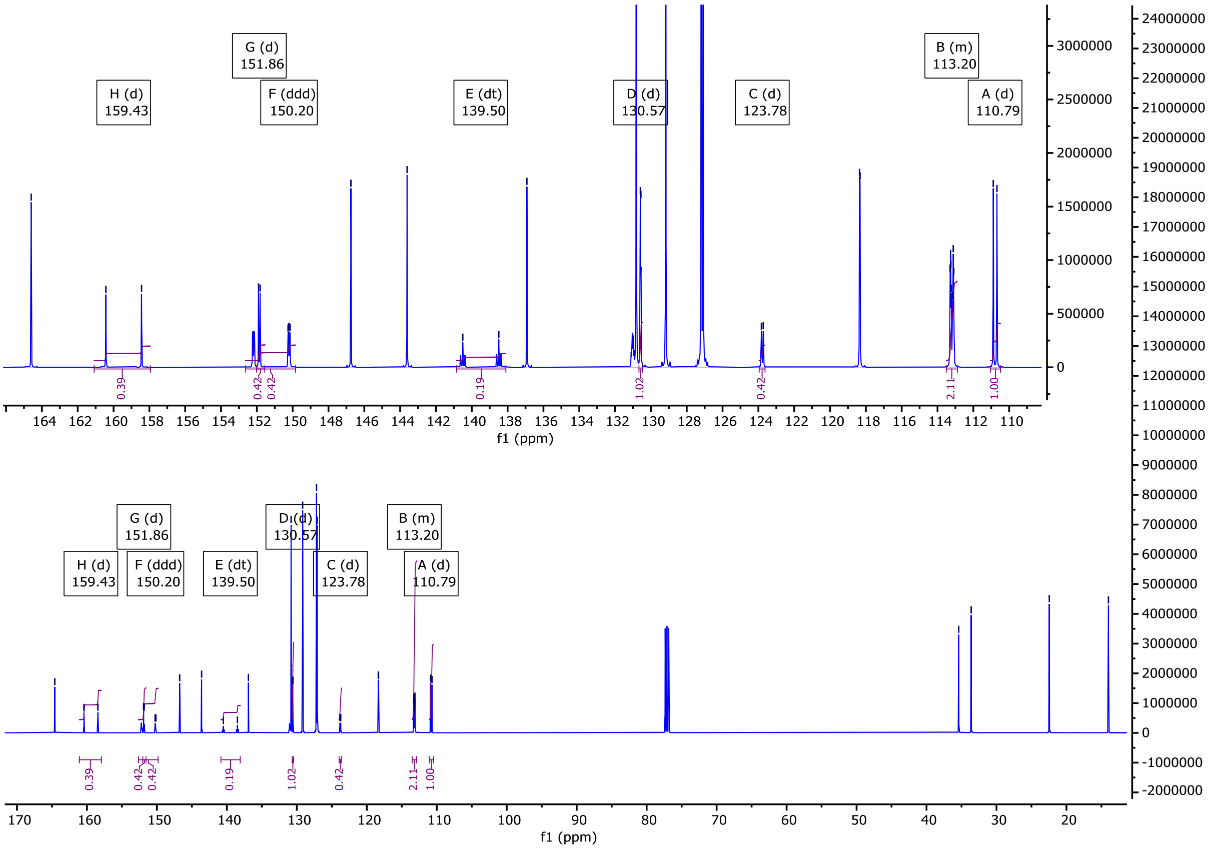
H (d) 159.43
G (d) 151.86
F (ddd) 150.20
E (dt) 139.50
D (d) 130.57
C (d) 123.78
B (m) 113.20
A (d) 110.79
f1 (ppm)

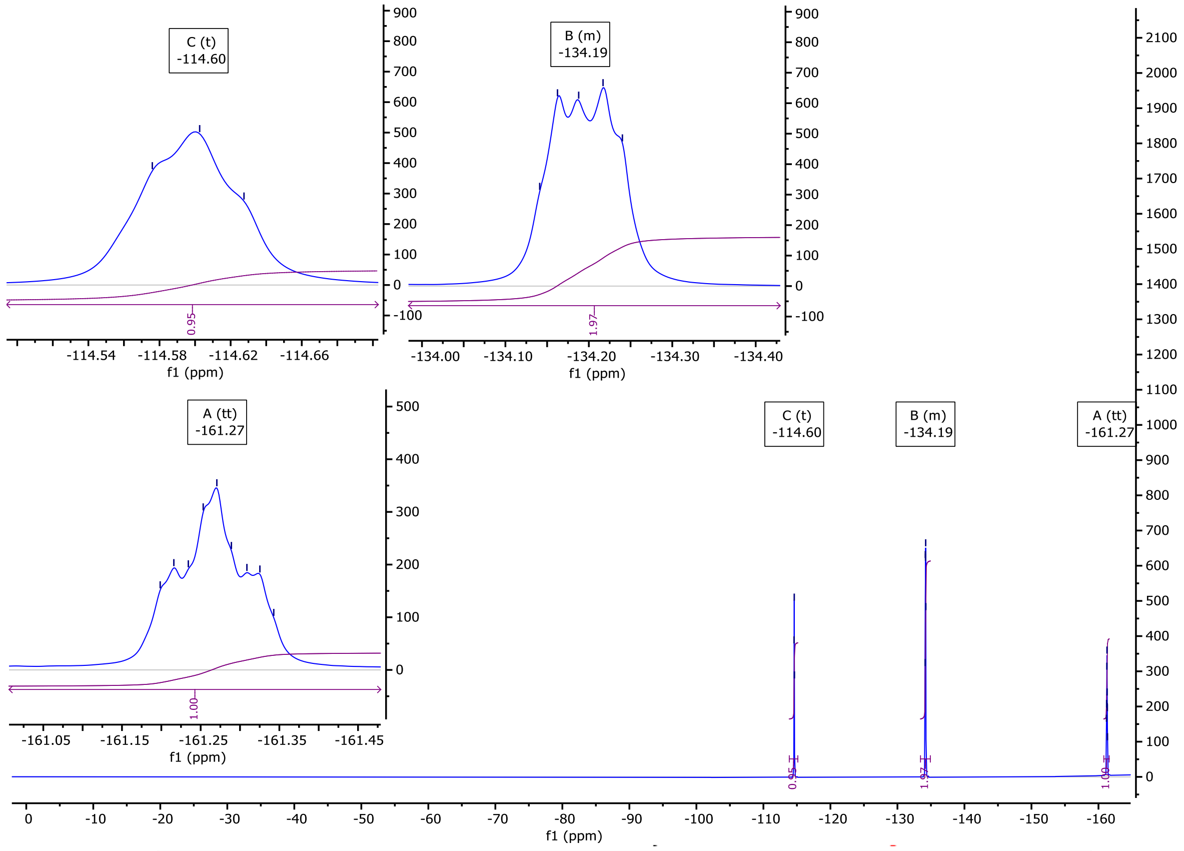

C (t)
-114.60
B (m)
-134.19
A (tt)
-161.27
f1 (ppm)

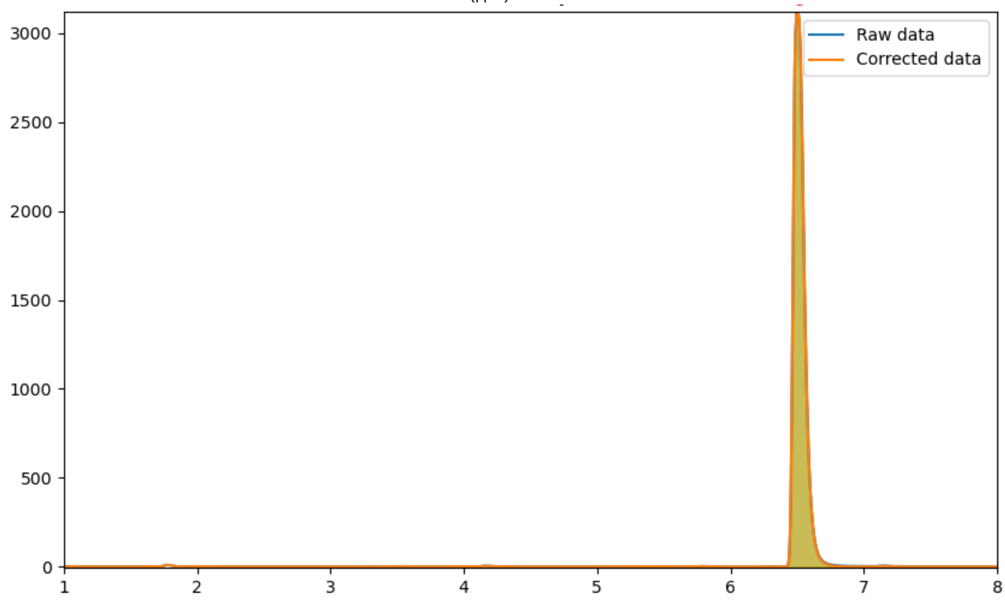

Raw data
Corrected data

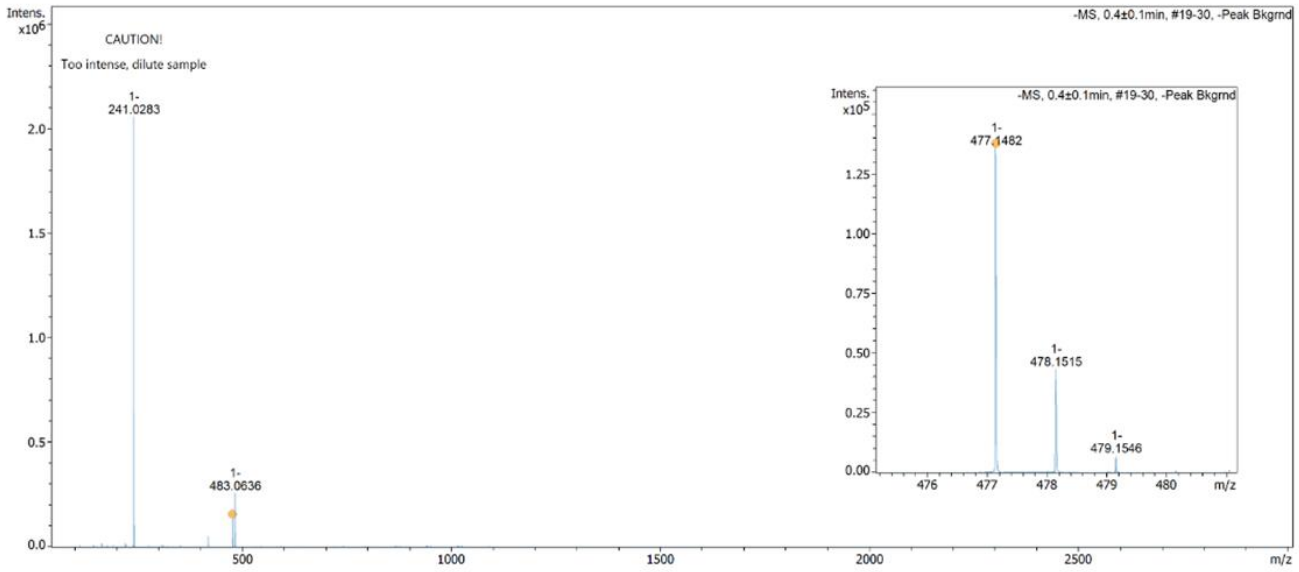

Fig. S16. ^{1}H [top], $^{13}C\{^{1}H\}$ [middle], and ^{19}F NMR spectra, HPLC chromatogram, and HRMS Spectra for **3** (**PPZGU-4-F**).

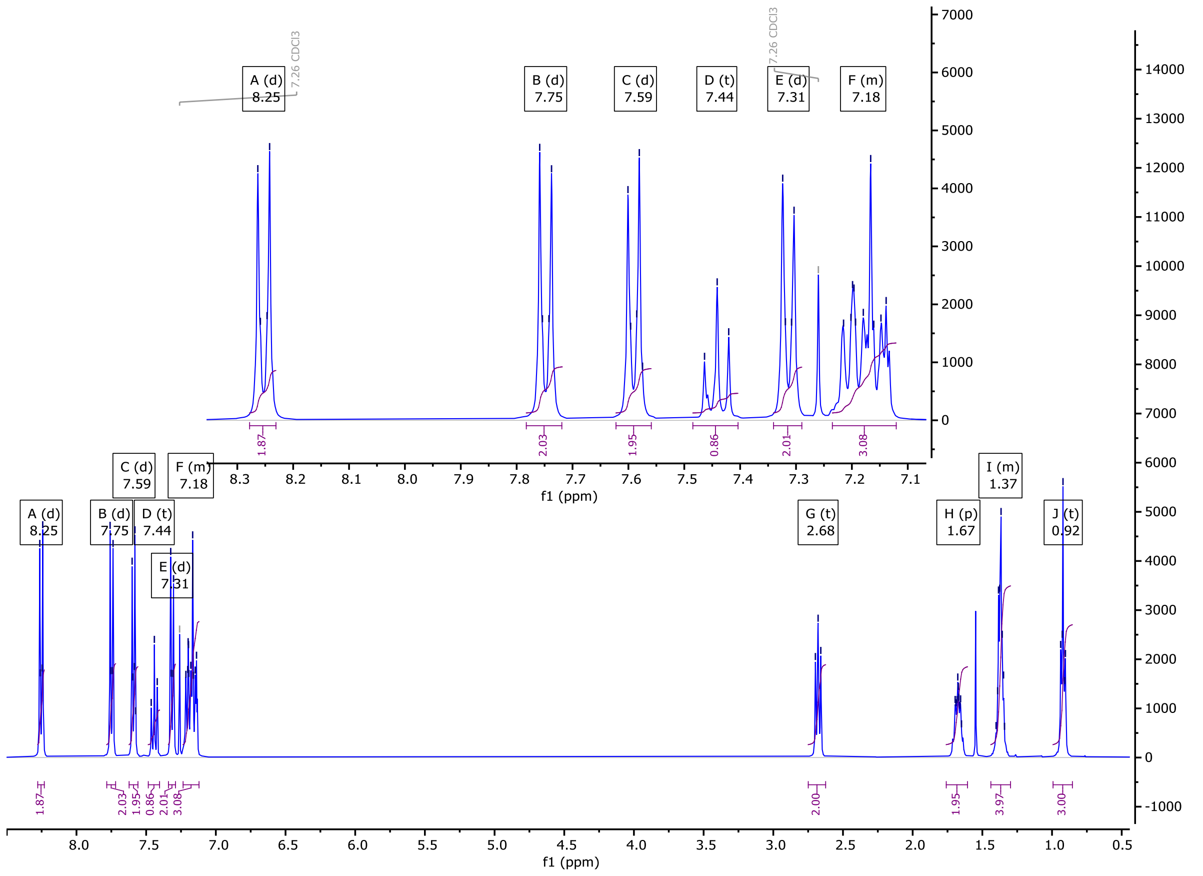

A (d)
8.25
B (d)
7.75
C (d)
7.59
D (t)
7.44
E (d)
7.31
F (m)
7.18
G (t)
2.68
H (p)
1.67
I (m)
1.37
J (t)
0.92
7.26 CDCl3
f1 (ppm)

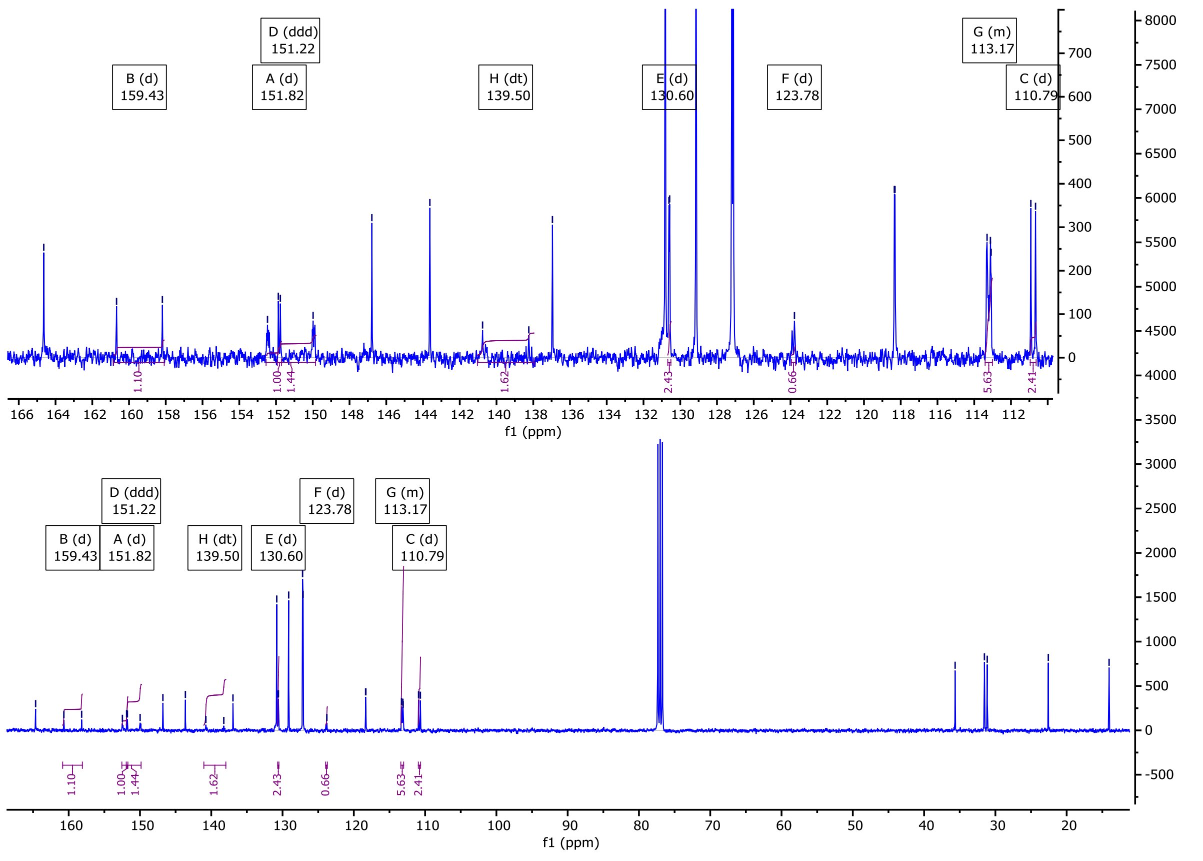

B (d)
159.43
A (d)
151.82
D (ddd)
151.22
H (dt)
139.50
E (d)
130.60
F (d)
123.78
G (m)
113.17
C (d)
110.79
f1 (ppm)

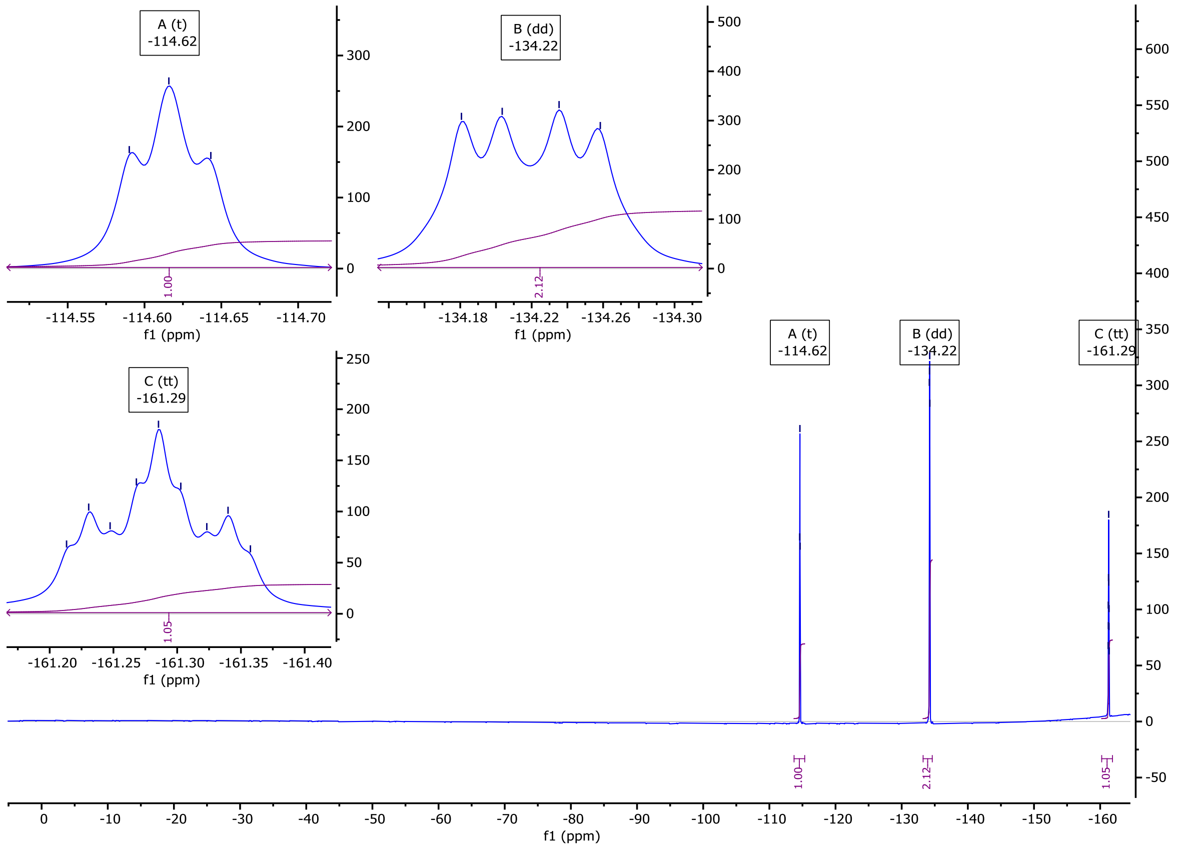

A (t)
-114.62
B (dd)
-134.22
C (tt)
-161.29
f1 (ppm)

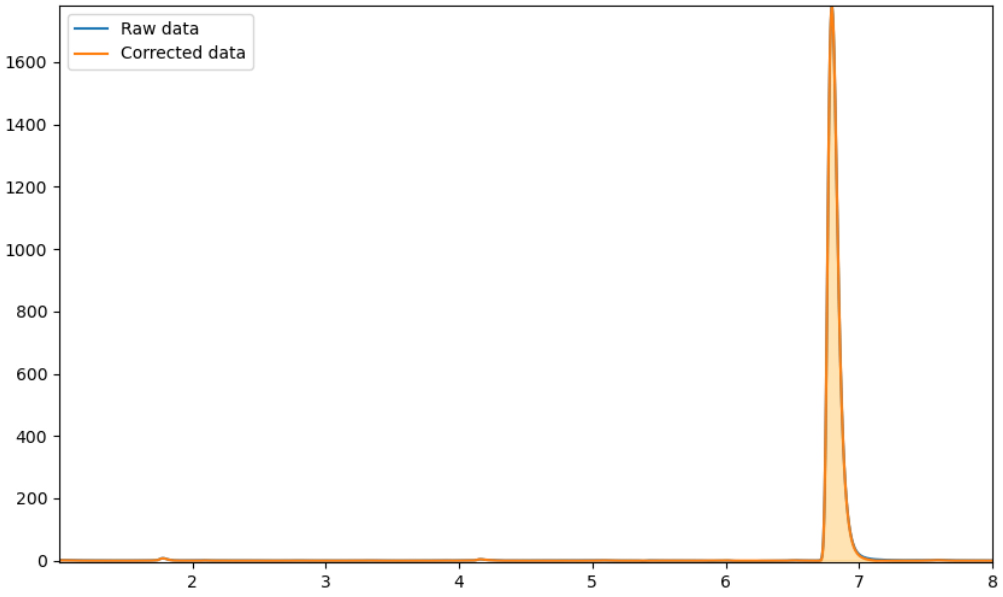

Raw data
Corrected data

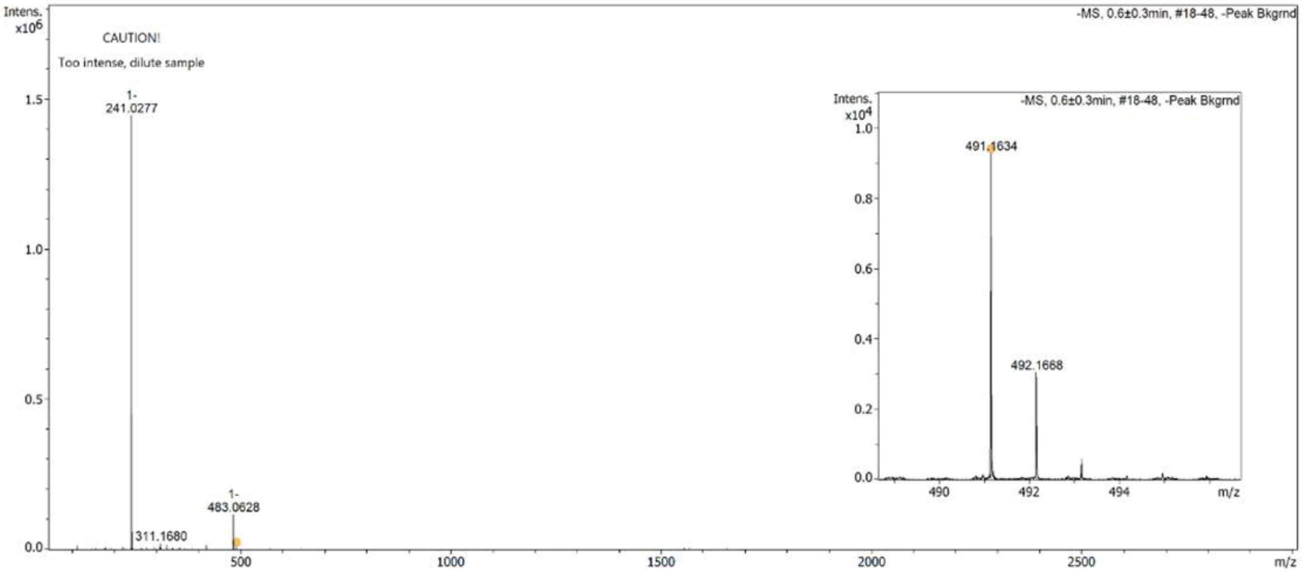

Fig. S17. ^{1}H [top], $^{13}C\{^{1}H\}$ [middle], and ^{19}F NMR spectra, HPLC chromatogram, and HRMS Spectra for **4** (**PPZGU-5-F**).

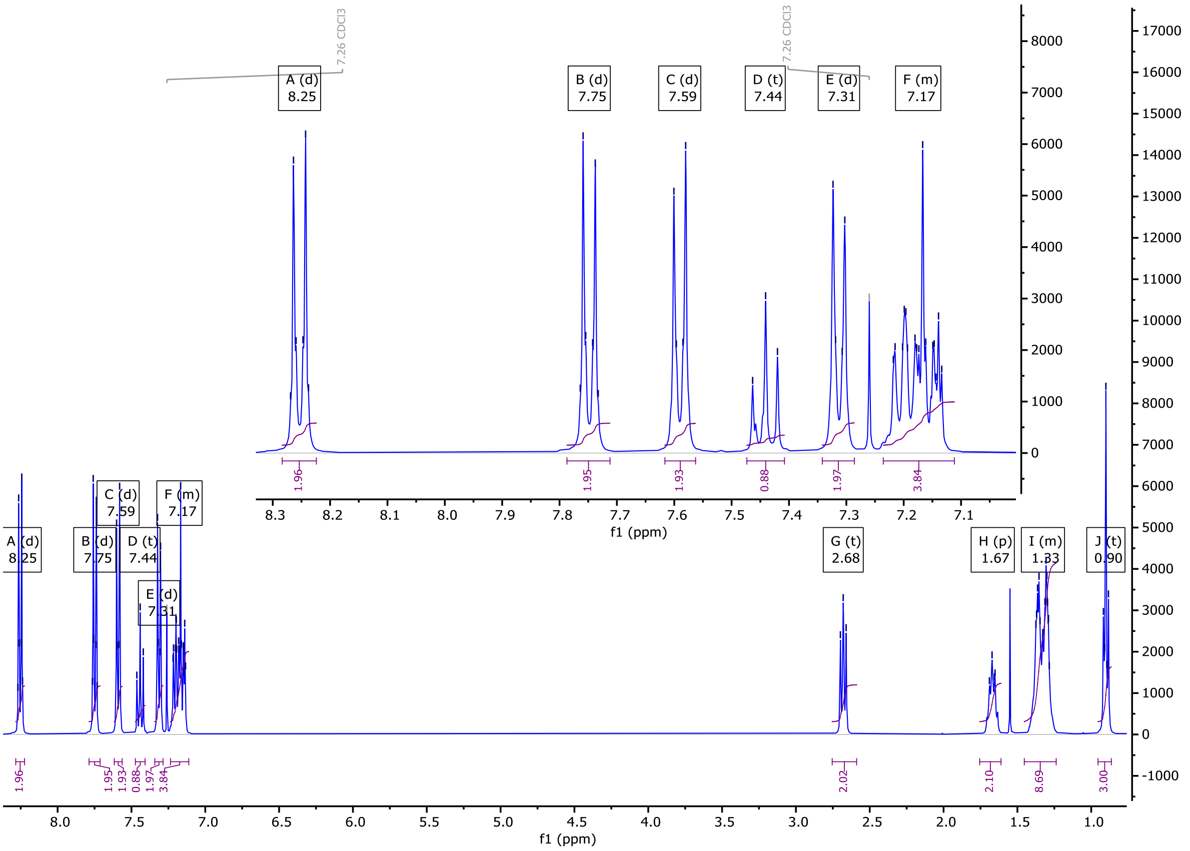
A (d) 8.25
B (d) 7.75
C (d) 7.59
D (t) 7.44
E (d) 7.31
F (m) 7.17
G (t) 2.68
H (p) 1.67
I (m) 1.33
J (t) 0.90
7.26 CDCl3
f1 (ppm)

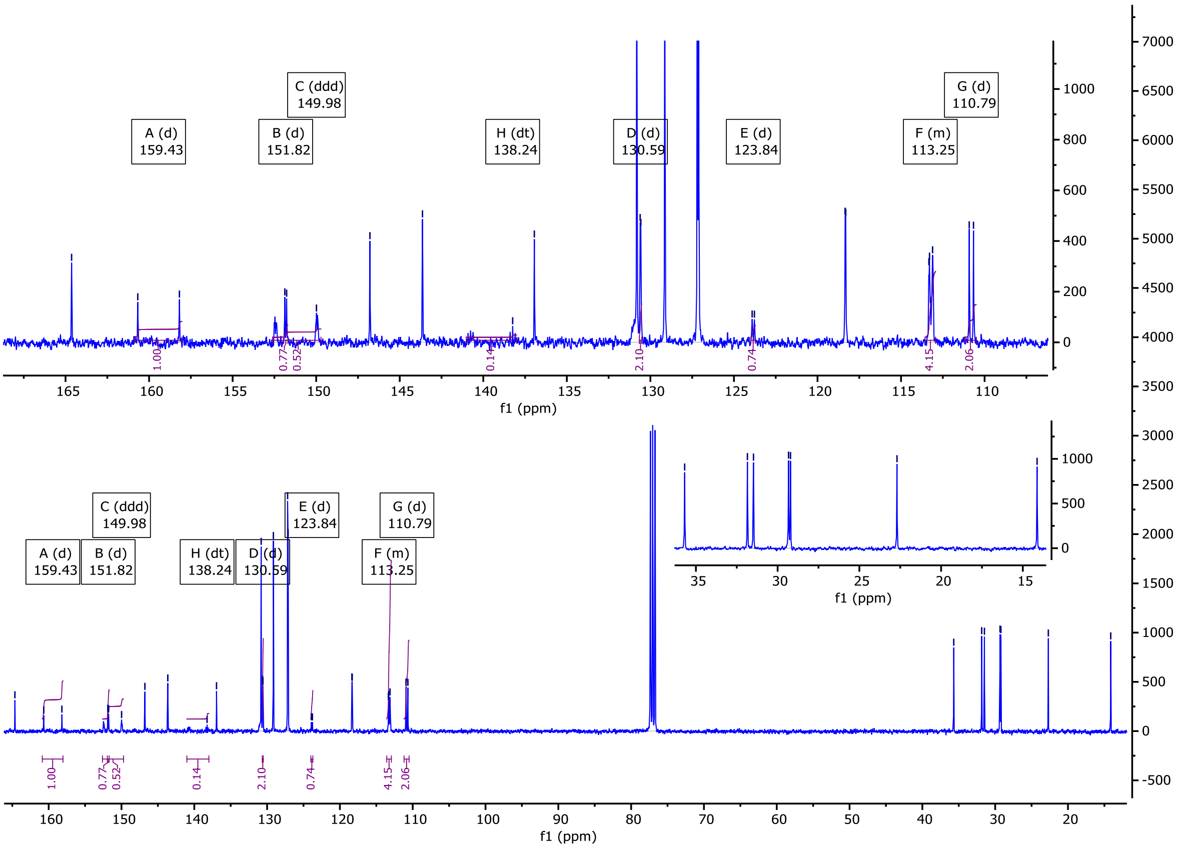
A (d) 159.43
B (d) 151.82
C (ddd) 149.98
H (dt) 138.24
D (d) 130.59
E (d) 123.84
F (m) 113.25
G (d) 110.79
f1 (ppm)

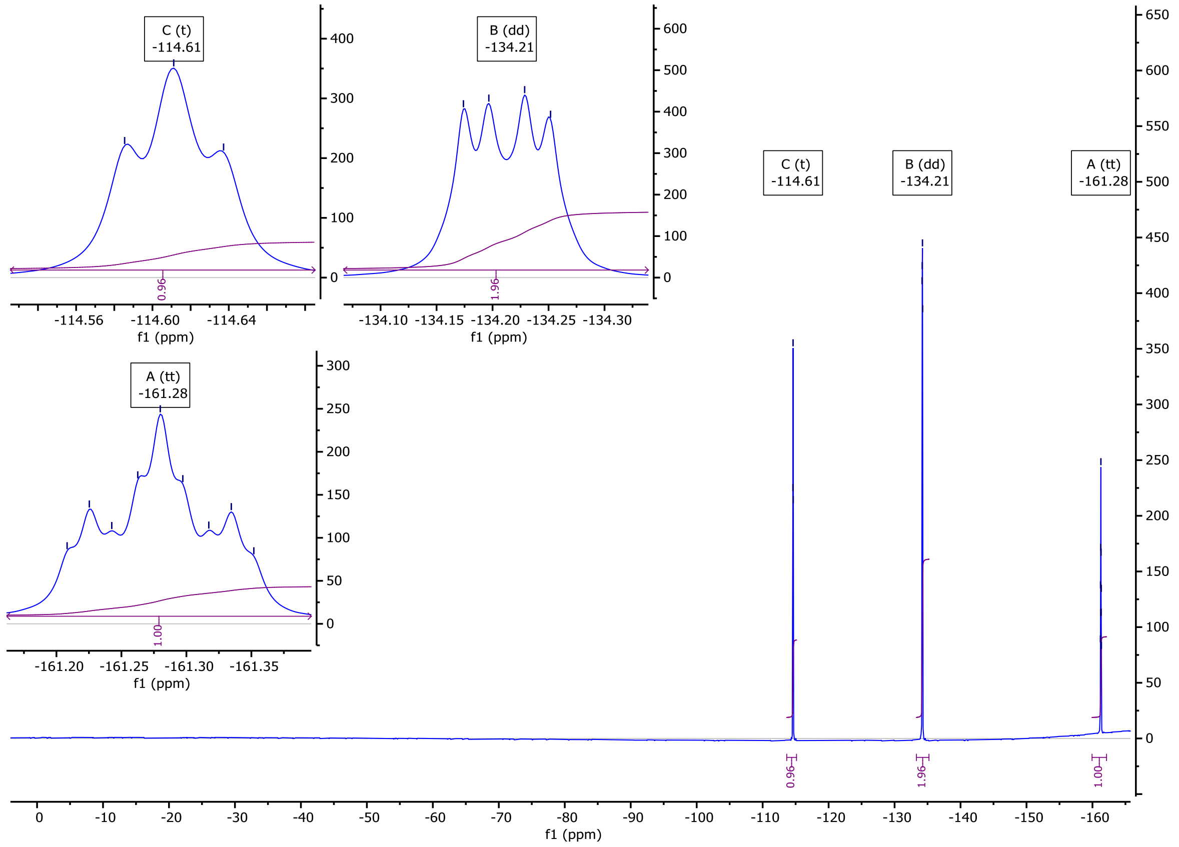
C (t)
-114.61
B (dd)
-134.21
A (tt)
-161.28
f1 (ppm)

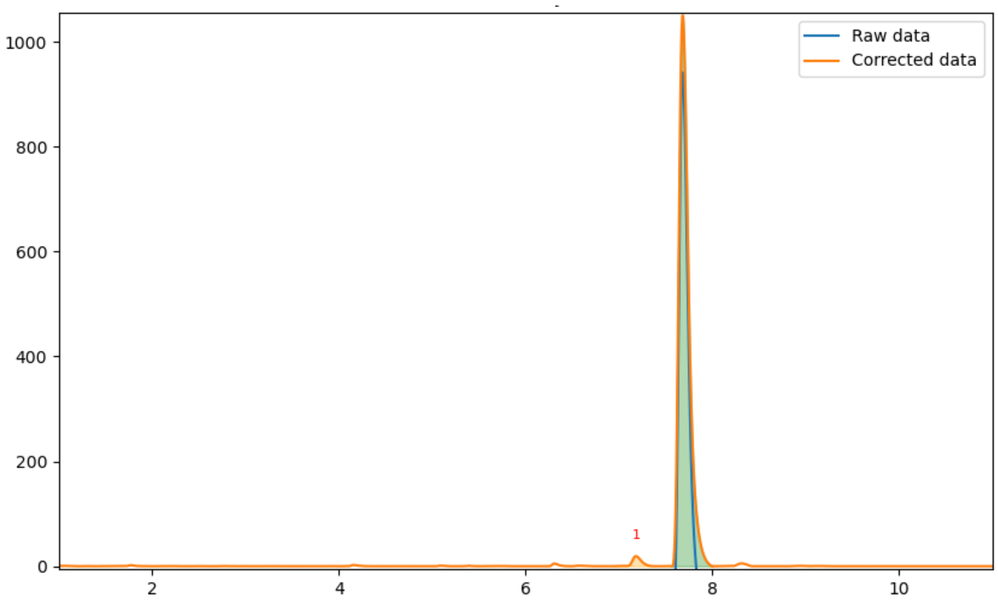
Raw data
Corrected data

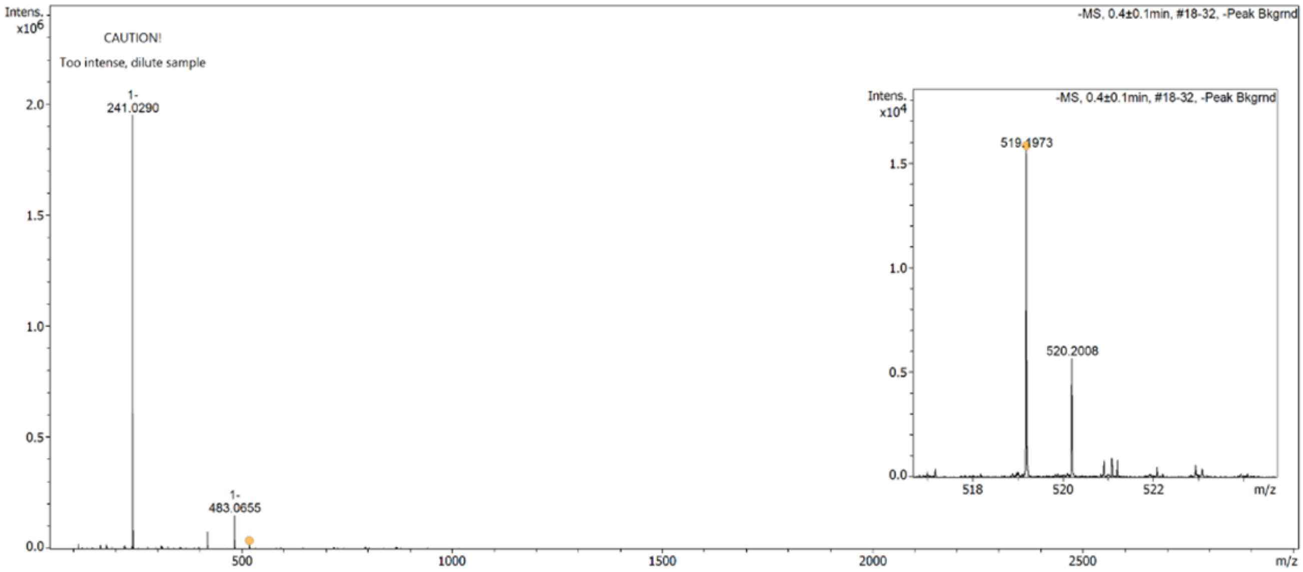

Fig. S18. ^{1}H [top], ^{13}C{^{1}H} [middle], and ^{19}F NMR spectra, HPLC chromatogram, and HRMS Spectra for **5** (**PPZGU-7-F**).

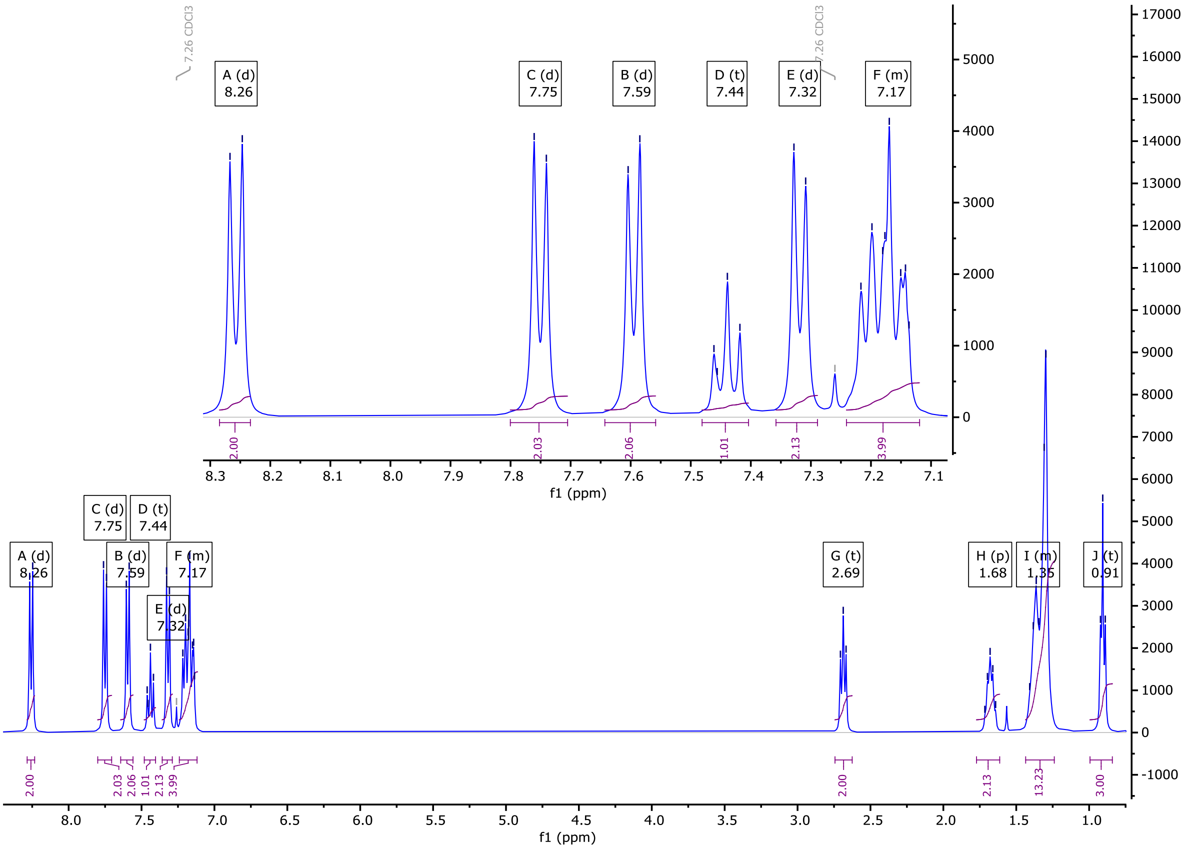
7.26 CDCl3
A (d) 8.26
B (d) 7.59
C (d) 7.75
D (t) 7.44
E (d) 7.32
F (m) 7.17
G (t) 2.69
H (p) 1.68
I (m) 1.35
J (t) 0.91
f1 (ppm)

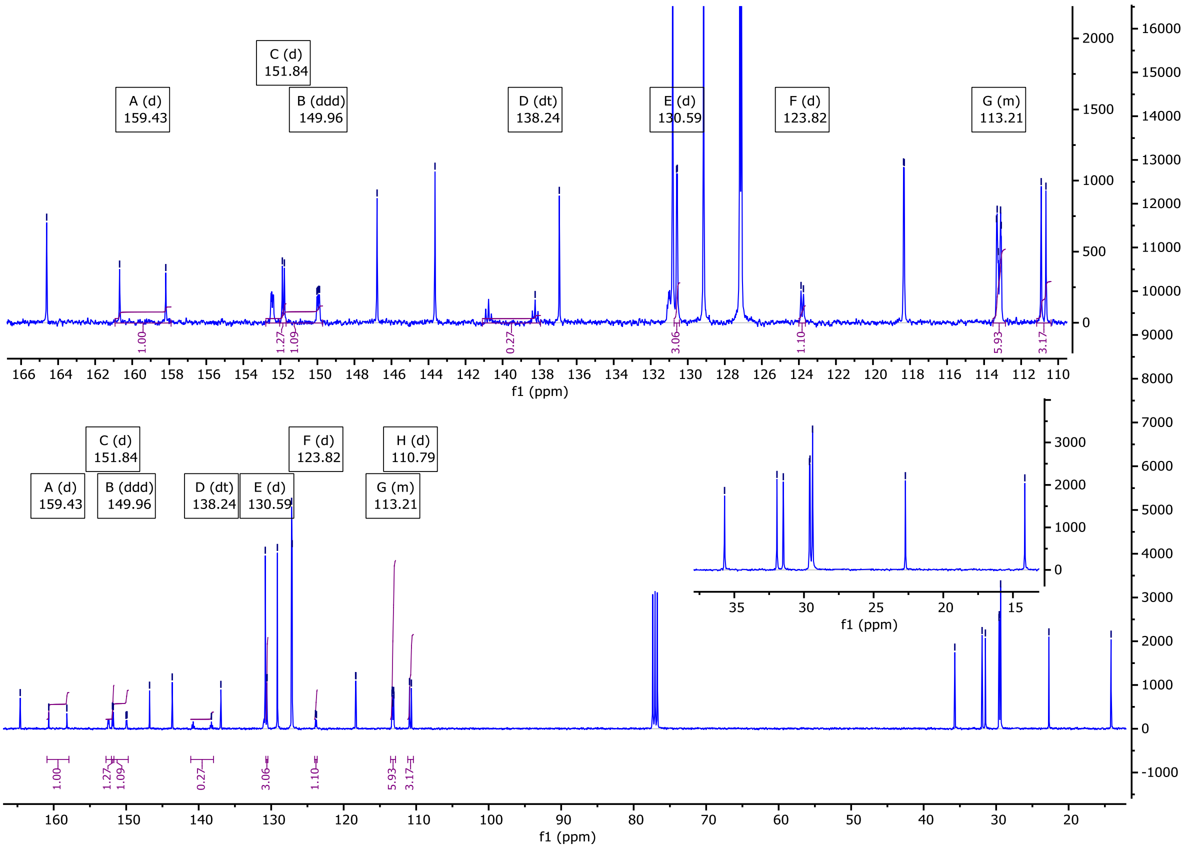
A (d) 159.43
B (ddd) 149.96
C (d) 151.84
D (dt) 138.24
E (d) 130.59
F (d) 123.82
G (m) 113.21
H (d) 110.79
f1 (ppm)

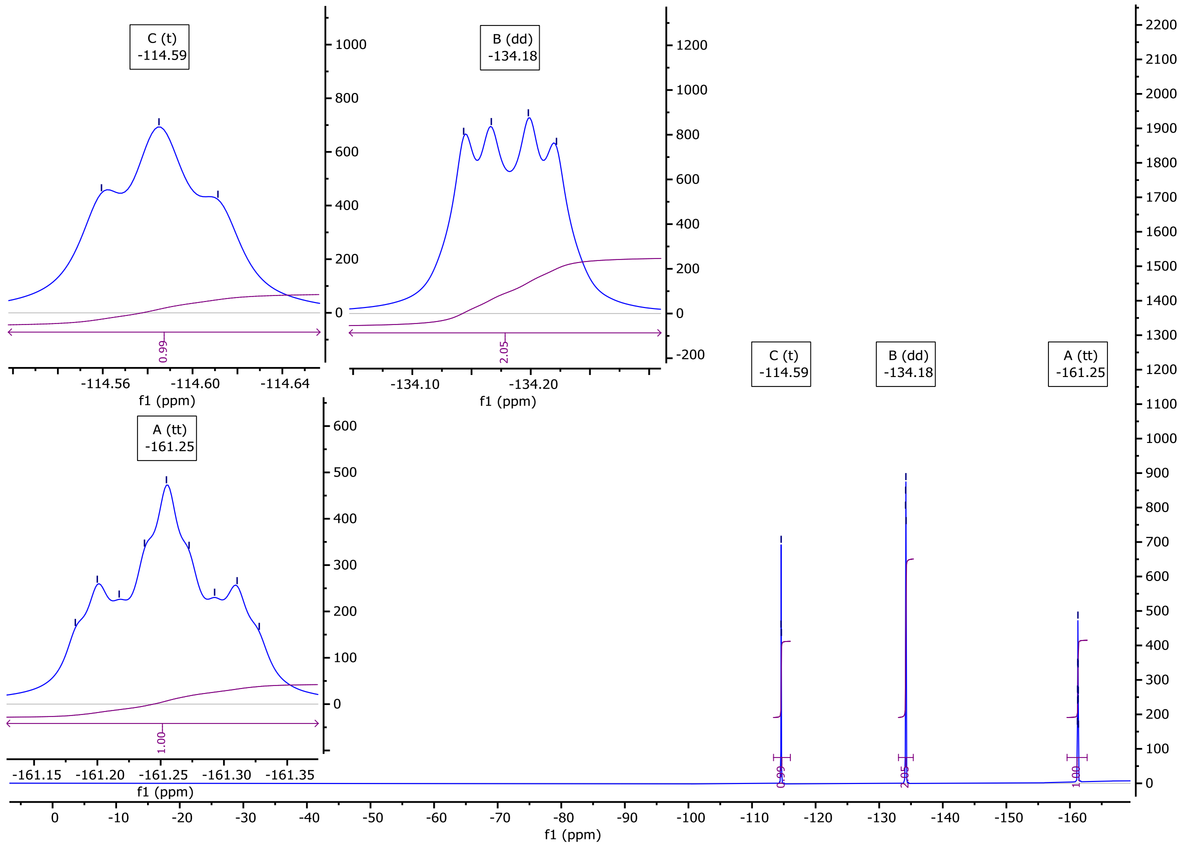
C (t)
-114.59
B (dd)
-134.18
A (tt)
-161.25
0.99
2.05
1.00
f1 (ppm)

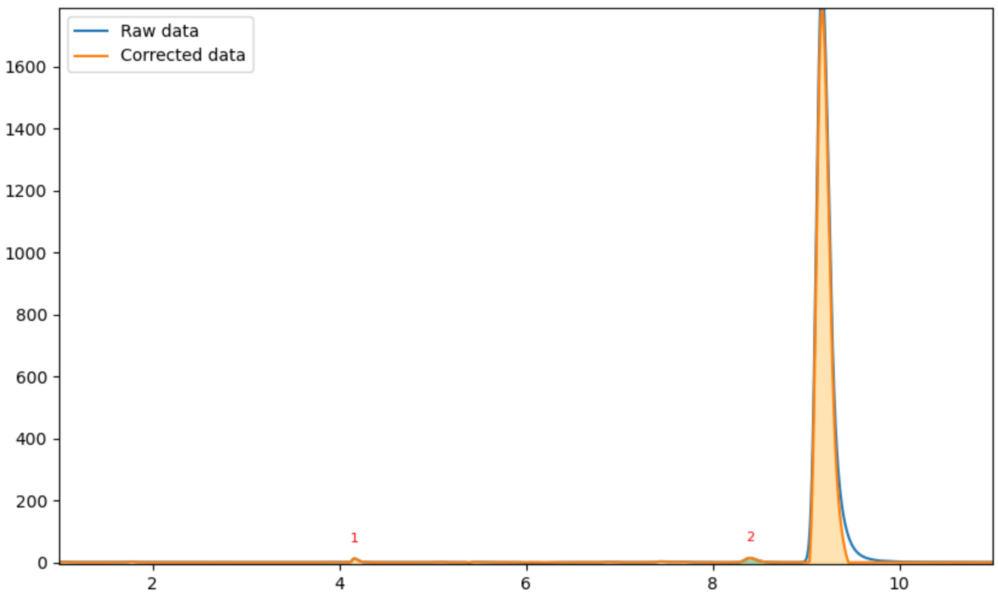
Raw data
Corrected data
1
2

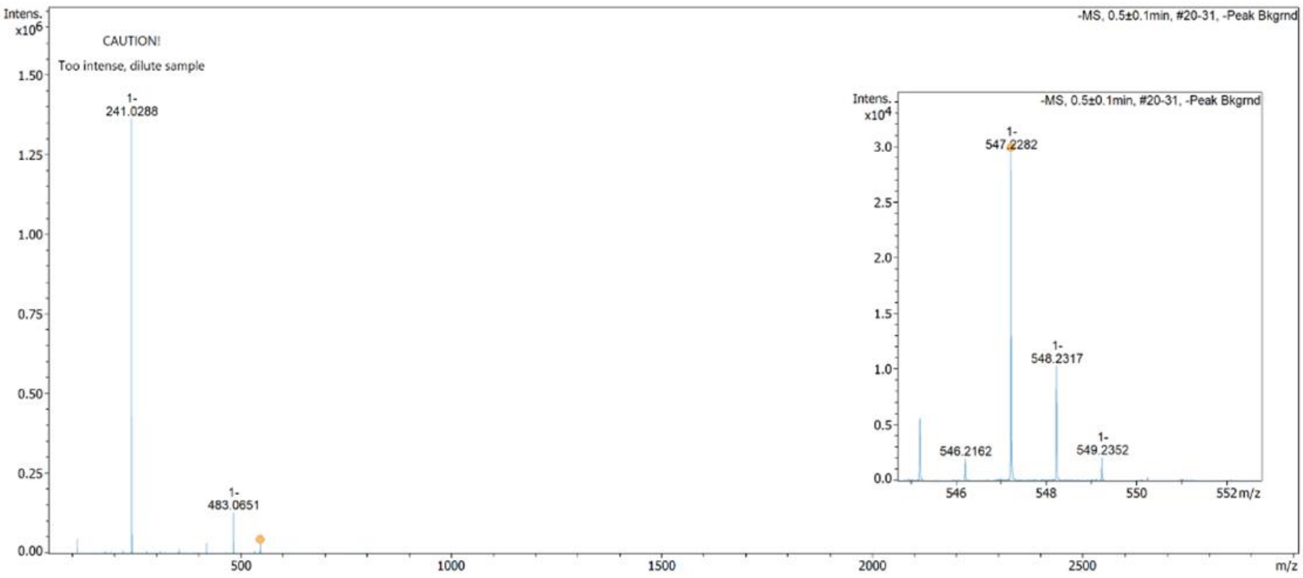

Fig. S19. ^{1}H [top], ^{13}C{^{1}H} [middle], and ^{19}F NMR spectra, HPLC chromatogram, and HRMS Spectra for **6** (**PPZGU-9-F**).

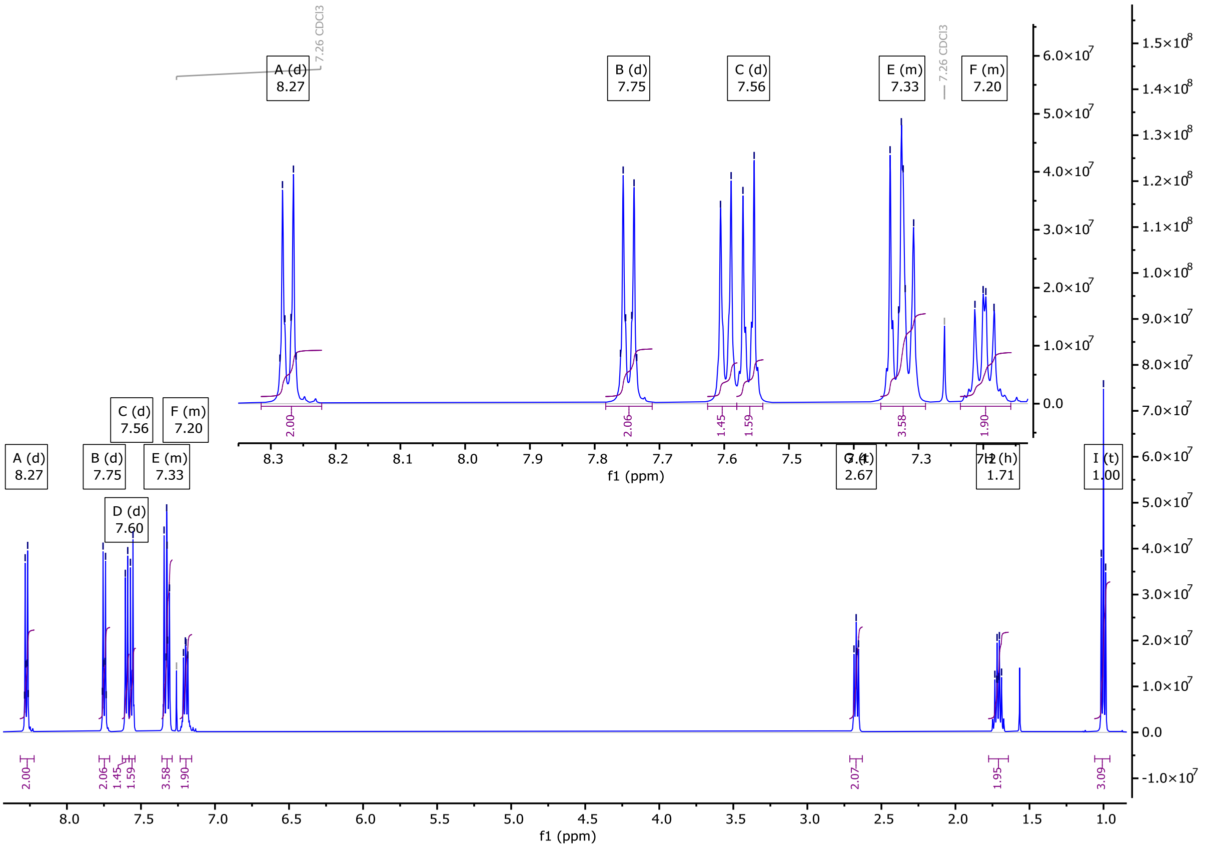
A (d) 8.27
B (d) 7.75
C (d) 7.56
E (m) 7.33
F (m) 7.20
7.26 CDCl3
D (d) 7.60
G (t) 2.67
H (h) 1.71
I (t) 1.00
f1 (ppm)

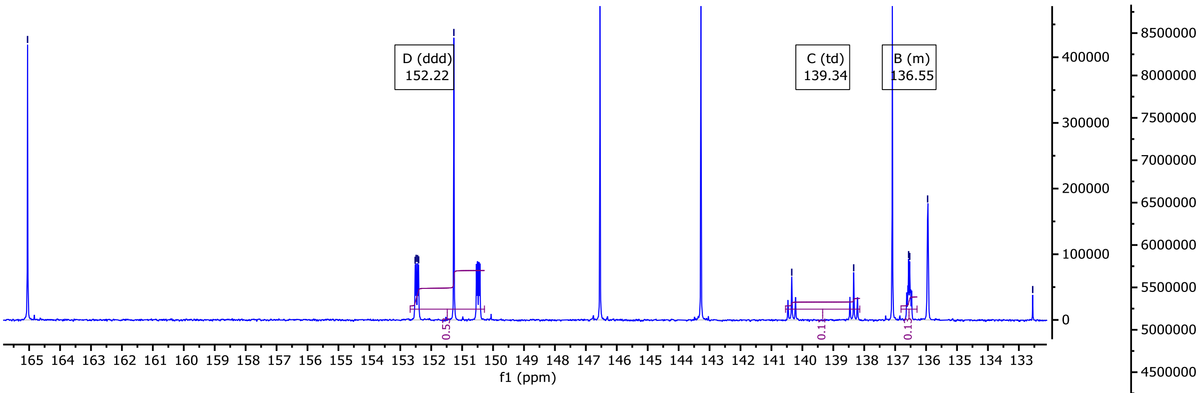
D (ddd) 152.22
C (td) 139.34
B (m) 136.55
f1 (ppm)

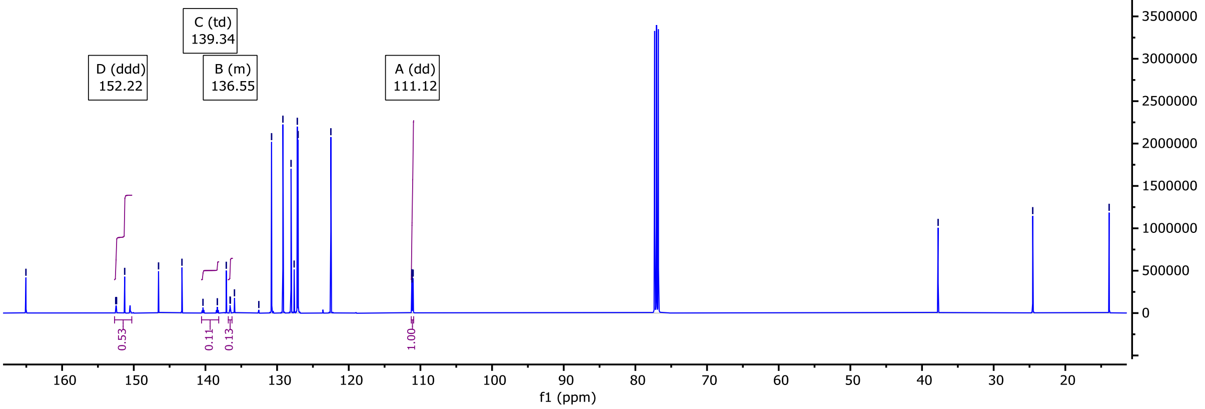
D (ddd) 152.22
C (td) 139.34
B (m) 136.55
A (dd) 111.12
f1 (ppm)

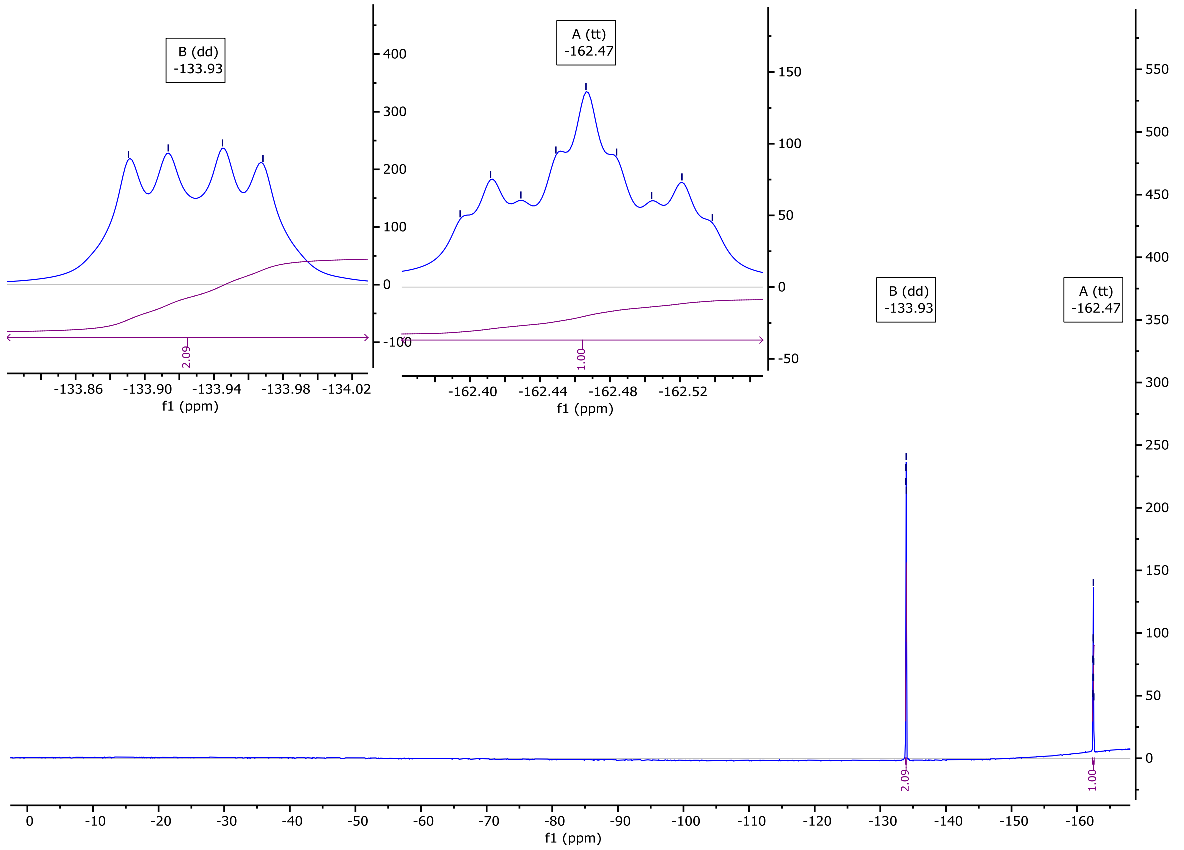
B (dd)
-133.93
A (tt)
-162.47
2.09
1.00
f1 (ppm)

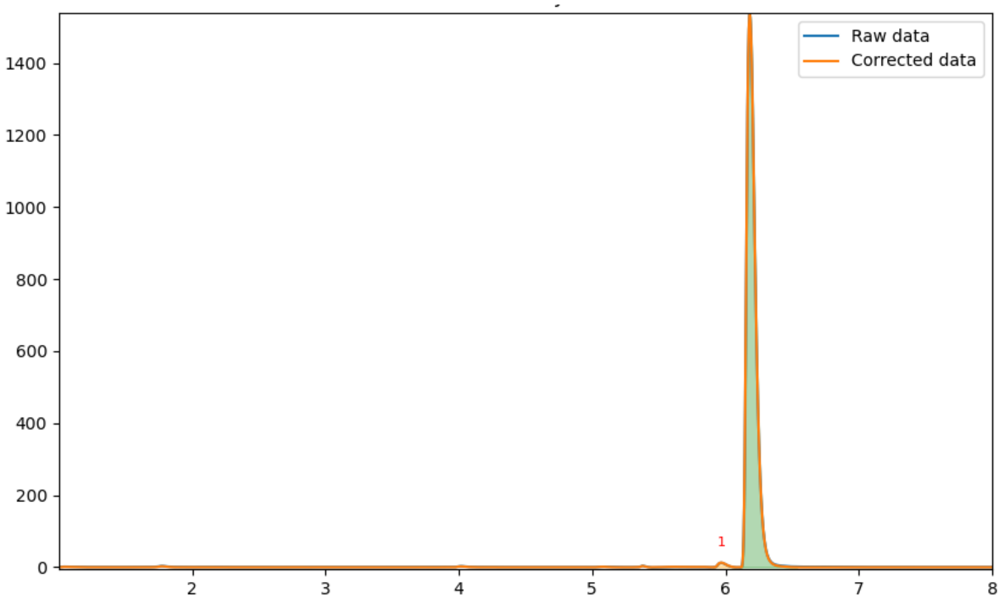
Raw data
Corrected data
1

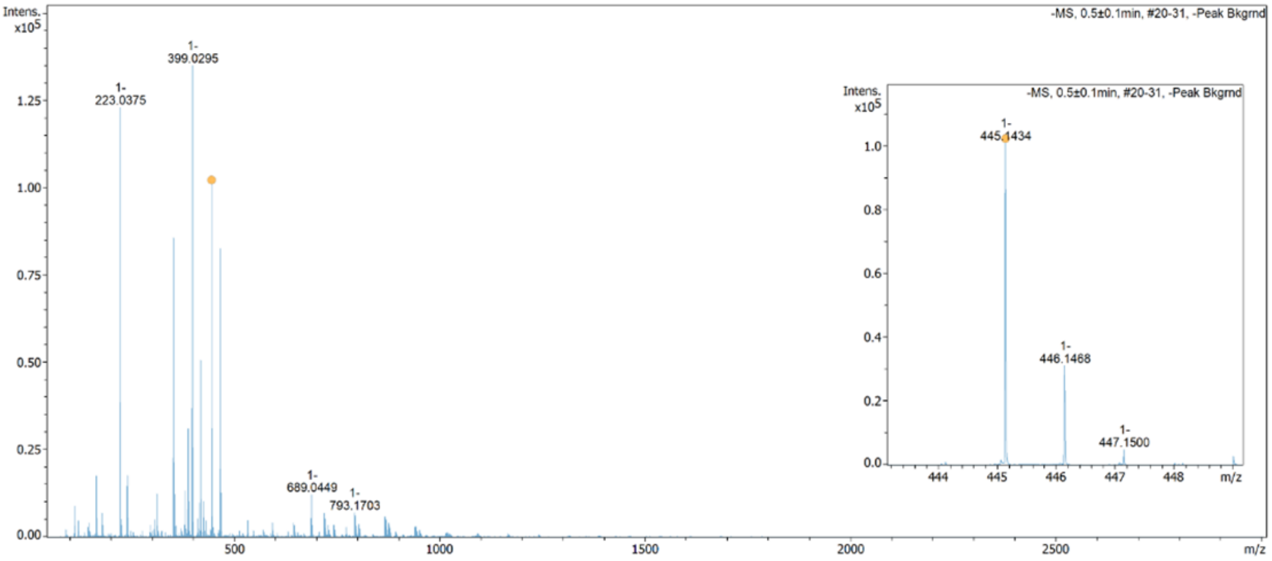

Fig. S20. ^{1}H [top], $^{13}C\{^{1}H\}$ [middle], and ^{19}F NMR spectra, HPLC chromatogram, and HRMS Spectra for **7** (**PPZPU-3-F**).

4 Supplementary References

[44] Martinot-Lagarde Ph., Direct electrical measurement of the permanent polarization of a ferroelectric chiral smectic C liquid crystal, J. Physique Lett. 38, 17 (1977).

[45] K. Miyasato, S. Abe, H. Takezoe, A. Fukuda, and E. Kuze, Direct Method with Triangular Waves for Measuring Spontaneous Polarization in Ferroelectric Liquid Crystals, Jpn J Appl Phys 22, L661 (1983).

[46] K. S. Cole and R. H. Cole, Dispersion and Absorption in Dielectrics I. Alternating Current Characteristics, J Chem Phys 9, 341 (1941).

[47] S. Havriliak and S. Negami, A complex plane representation of dielectric and mechanical relaxation processes in some polymers, Polymer (Guildf) 8, 161 (1967).

[48] M. J. Frisch, G. W. Trucks, H. B. Schlegel, G .E. Scuseria, M. A. Robb, J. R. Cheeseman, G. Scalmani, V. Barone, B. Mennucci, G. A. Petersson, H. Nakatsuji, M. Caricato, X. Li, H. P. Hratchian, A. F. Izmaylov, J. Bloino, G. Zheng, J. L. Sonnenberg, M. Hada, M. Ehara, K. Toyota, R. Fukuda, J. Hasegawa, M. Ishida, T. Nakajima, Y. Honda, O. Kitao, H. Nakai, T. Vreven, J. A. Montgomery Jr., J. E. Peralta, F. Ogliaro, M. J. Bearpark, J. Heyd, E. N. Brothers, K. N. Kudin, V. N. Staroverov, R. Kobayashi, J. Normand, K. Raghavachari, A. P. Rendell, J. C. Burant, S. S. Iyengar, J. Tomasi, M. Cossi, N. Rega, N. J. Millam, M. Klene, J. E. Knox, J. B. Cross, V. Bakken, C. Adamo, J. Jaramillo, R. Gomperts, R. E. Stratmann, O. Yazyev, A. J. Austin,R. Cammi, C. Pomelli, J. W. Ochterski, R. L. Martin, K. Morokuma, V. G. Zakrzewski, G. A. Voth, P. Salvador, J. J. Dannenberg, S. Dapprich, A. D. Daniels, O ̈. Farkas, J. B. Foresman ,J. V. Ortiz, J. Cioslowskiand, D. J. Fox, Gaussian 016, Revision E.01, Gaussian, Inc., Wallingford CT, 2016.

[49] A. D. Becke, Density-functional thermochemistry. III. The role of exact exchange, J Chem Phys 98, 5648 (1993).

[50] C. Lee, W. Yang, and R. G. Parr, Development of the Colle-Salvetti correlation-energy formula into a functional of the electron density, Phys Rev B 37, 785 (1988).

[51] T. H. Dunning, Gaussian basis sets for use in correlated molecular calculations. I. The atoms boron through neon and hydrogen, J Chem Phys 90, 1007 (1989).

[52] S. Grimme, S. Ehrlich, and L. Goerigk, Effect of the damping function in dispersion corrected density functional theory, J Comput Chem 32, 1456 (2011).

[53] J. Wang, P. Cieplak, and P. A. Kollman, How well does a restrained electrostatic potential (RESP) model perform in calculating conformational energies of organic and biological molecules?, J Comput Chem 21, 1049 (2000).

[54] D. A. Case et al., AmberTools, J Chem Inf Model 63, 6183 (2023).

[55] A. W. Sousa da Silva and W. F. Vranken, ACPYPE - AnteChamber PYthon Parser interfacE, BMC Res Notes 5, 367 (2012).

[56] B. Hess, H. Bekker, H. J. C. Berendsen, and J. G. E. M. Fraaije, LINCS: A Linear Constraint Solver for molecular simulations, J Comput Chem 18, 1463 (1997).

[57] R. T. McGibbon, K. A. Beauchamp, M. P. Harrigan, C. Klein, J. M. Swails, C. X. Hernández, C. R. Schwantes, L. P. Wang, T. J. Lane, and V. S. Pande, MDTraj: A Modern Open Library for the Analysis of Molecular Dynamics Trajectories, Biophys J 109, 1528 (2015).

[58] A. Bondi, van der Waals Volumes and Radii, J Phys Chem 68, 441 (1964).
